# Abnormal Brain Iron Metabolism in *Irp2* Deficient Mice Is Associated with Mild Neurological and Behavioral Impairments

**DOI:** 10.1371/journal.pone.0098072

**Published:** 2014-06-04

**Authors:** Kimberly B. Zumbrennen-Bullough, Lore Becker, Lillian Garrett, Sabine M. Hölter, Julia Calzada-Wack, Ilona Mossbrugger, Leticia Quintanilla-Fend, Ildiko Racz, Birgit Rathkolb, Thomas Klopstock, Wolfgang Wurst, Andreas Zimmer, Eckhard Wolf, Helmut Fuchs, Valerie Gailus-Durner, Martin Hrabě de Angelis, Steven J. Romney, Elizabeth A. Leibold

**Affiliations:** 1 Program in Anemia Signaling Research, Division of Nephrology, Program in Membrane Biology, Center for Systems Biology, Massachusetts General Hospital, Harvard Medical School, Boston, Massachusetts, United States of America; 2 German Mouse Clinic, Helmholtz-Zentrum München, German Research Center for Environmental Health, Neuherberg, Germany; 3 Institute of Experimental Genetics, Helmholtz-Zentrum München, German Research Center for Environmental Health, Neuherberg, Germany; 4 Institute of Development Genetics, Helmholtz-Zentrum München, German Research Center for Environmental Health, Neuherberg, Germany; 5 Institute of Pathology, Helmholtz-Zentrum München, German Research Center for Environmental Health, Neuherberg, Germany; 6 Institute of Molecular Psychiatry, Life & Brain Center, University of Bonn, Bonn, Germany; 7 Institute of Molecular Animal Breeding and Biotechnology, Gene Center, Ludwig-Maximilians-Universitat, Munich, Germany; 8 Department of Neurology, Friedrich-Baur-Institute, Klinikum der Ludwig-Maximilians-Universitat, Munich, Germany; 9 Chair of Experimental Genetics, Center of Life and Food Sciences Weihenstephan, Technische Universitat München, Freising, Germany; 10 German Center for Diabetes Research, Neuherberg, Germany; 11 Chair of Developmental Genetics, Technische Universitat München, Freising-Weihenstephan, Germany; 12 Max Planck Institute of Psychiatry, Munich, Germany; 13 Deutsches Zentrum für Neurodegenerative Erkrankungen, Munich, Germany; 14 Munich Cluster for Systems Neurology, Munich, Germany; 15 University of Utah, Department of Medicine, Division of Hematology and Hematological Malignancies, Salt Lake City, Utah, United States of America; Lady Davis Institute for Medical Research/McGill University, Canada

## Abstract

Iron Regulatory Protein 2 (Irp2, Ireb2) is a central regulator of cellular iron homeostasis in vertebrates. Two global knockout mouse models have been generated to explore the role of Irp2 in regulating iron metabolism. While both mouse models show that loss of Irp2 results in microcytic anemia and altered body iron distribution, discrepant results have drawn into question the role of Irp2 in regulating brain iron metabolism. One model shows that aged Irp2 deficient mice develop adult-onset progressive neurodegeneration that is associated with axonal degeneration and loss of Purkinje cells in the central nervous system. These mice show iron deposition in white matter tracts and oligodendrocyte soma throughout the brain. A contrasting model of global Irp2 deficiency shows no overt or pathological signs of neurodegeneration or brain iron accumulation, and display only mild motor coordination and balance deficits when challenged by specific tests. Explanations for conflicting findings in the severity of the clinical phenotype, brain iron accumulation and neuronal degeneration remain unclear. Here, we describe an additional mouse model of global Irp2 deficiency. Our aged *Irp2^−/−^* mice show marked iron deposition in white matter and in oligodendrocytes while iron content is significantly reduced in neurons. Ferritin and transferrin receptor 1 (TfR1, Tfrc), expression are increased and decreased, respectively, in the brain from *Irp2^−/−^* mice. These mice show impairments in locomotion, exploration, motor coordination/balance and nociception when assessed by neurological and behavioral tests, but lack overt signs of neurodegenerative disease. Ultrastructural studies of specific brain regions show no evidence of neurodegeneration. Our data suggest that Irp2 deficiency dysregulates brain iron metabolism causing cellular dysfunction that ultimately leads to mild neurological, behavioral and nociceptive impairments.

## Introduction

Iron is essential for growth and proliferation of mammalian cells due to its role as a protein cofactor for hemoglobin synthesis, DNA synthesis and mitochondrial respiration. Regulation of cellular iron content is crucial since excess cellular iron catalyzes the generation of reactive oxygen species that damage DNA and proteins, while cellular iron deficiency causes cell cycle arrest and cell death. Dysregulation of iron homeostasis caused by iron excess or iron deficiency leads to hematological, metabolic and neurodegenerative diseases [1]–[3].

The central nervous system is particularly vulnerable to altered iron metabolism. Iron deficiency perinatally or postnatally can cause permanent neurocognitive and motor impairments in humans [4]–[6] and in rodent models [7]. Abnormally high brain iron is associated with common neurodegenerative disorders, including Parkinson's and Alzheimer's diseases, as well as rare inherited diseases known as Neurodegeneration with Brain Iron Accumulation (NBIA) [3], [8], which manifest as movement disorders. Whether brain iron accumulation is the primary pathologic event causing neurodegeneration or whether iron accumulation is a secondary event caused by neuronal death is unclear. However, two NBIA diseases, hereditary ferritinopathy and aceruloplasminemia, caused by mutations in the ferritin-L subunit gene (*FTL*) [9] and in the ceruloplasmin (*CP*) gene [10], respectively, suggest that abnormal iron metabolism is the pathologic event leading to neurodegeneration in these disorders. These studies highlight the importance of maintaining brain iron within a physiological range to avoid the adverse consequences of iron depletion or excess.

Vertebrate cellular iron metabolism is regulated post-transcriptionally by iron regulatory protein 1 (Irp1, also known as Aco1) and Irp2 [11], [12]. Irps are cytosolic RNA-binding proteins that bind to iron-responsive elements (IREs) located in the 5′ or 3′ untranslated regions of mRNAs encoding proteins involved in iron sequestration (ferritin) and iron uptake (TfR1), respectively. When cells are iron-deficient, Irps bind IREs with high affinity inhibiting ferritin translation while stabilizing TfR1 mRNA. When cells are iron-sufficient, Irp1 is converted to an [4Fe-4S]-containing aconitase and Irp2 is degraded by iron-mediated proteasomal degradation [13]–[15], increasing ferritin translation and promoting TfR1 mRNA degradation. Irps thus regulate the amount of iron sequestered by ferritin and acquired by TfR1 ensuring that cells acquire adequate iron for their needs without it reaching toxic levels. Ferritin and TfR1 are the primary Irp-regulated target mRNAs; however, Irps regulate other IRE-containing mRNAs that encode proteins involved in the tricarboxylic acid cycle, heme biosynthesis, iron export, hypoxia and the cell cycle [12], [16].

Mouse models of Irp1 and Irp2 deficiency have been generated [17]–[19]. *Irp1^−/−^* mice display polycythemia due to derepression of the Irp1-specific target mRNA hypoxia-inducible factor 2α (Hif-2α; also known as Epas1) [20]–[22]. Two mouse models of global Irp2 deficiency display dysregulation of ferritin and TfR1and abnormal iron content in several tissues, and develop microcytic anemia and erythropoietic protoporphyria [17], [19], [23], [24]. *Irp2^−/−^* mice generated by LaVaute et al. [17] developed a progressive late-onset neurodegenerative disorder (>6 months old) characterized by tremors, abnormal gait, subtle kyphosis and hind-limb weakness. Neurological tests showed impaired neuromuscular performance and grooming activity in these mice and histochemical studies showed evidence of axonpathy in white matter that is associated with increased ferric iron and ferritin expression [17]. The severity of neurodegeneration and microcytic anemia is worse in *Irp2^−/−^* mice lacking one copy of Irp1 (*Irp2^−/−^;Irp1^+/−^*), indicating a dosage effect [25]. In contrast, fourteen month old *Irp2 ^−/−^* mice generated by Galy et al. [26] showed no overt signs of neurodegeneration, abnormal brain iron accumulation or evidence of neuronal degeneration, and displayed only mild motor coordination/balance impairments and reduced grooming activity when assessed by neurological and behavioral tests [26]. The cause of the differences in the severity of clinical phenotypes and neuropathology in these two *Irp2^−/−^* mouse models remains unclear.

Here, we describe neurological, behavioral and brain iron phenotypes in an additional global Irp2 deficient mouse model. We find that our *Irp2 ^−/−^* mice recapitulate the salient features of other *Irp2^−/−^* models, including microcytic anemia, erythropoietic protoporphyria, altered body iron distribution and dysregulation of ferritin and TfR1 in several tissues. Our aged *Irp2^−/−^* mice do not display overt signs of neurodegeneration, but show mild impairments in motor coordination/balance, locomotion and nociception. *Irp2^−/−^* mice have marked iron deposition in white matter and in oligodendrocytes, and iron deficiency in neurons without pathological evidence of neurodegeneration. We conclude that alterations in brain iron caused by Irp2 deficiency likely disrupts cellular function, and causes neurological, behavioral and nociception impairments.

## Materials and Methods

### Mice

The generation of *Irp2^−/−^* mice is described in Figure S1. *Irp2^−/−^* mice were generated on a C57BL/6J and 129/Sv background and backcrossed with C57BL/6J for five generations. *Irp2^−/−^* and WT littermates were obtained from intercrosses from *Irp2^+/−^* parents. Only male mice were used for this study. Mice used for neurological and behavioral experiments were 57–63 weeks (WT) and 38–45 weeks (*Irp2^−/−^)* and mice used for hematological/clinical analysis were 64–75 weeks (WT) and 49–71 weeks (*Irp2^−/−^*). Mice were kept in accordance with the recommendations in the Guide for the Care and Use of Laboratory Animals of the National Institutes of Health. The protocol was approved by the Institutional Animal Care and Use Committee (IACUC) of the University of Utah (Protocol Number: 13-02012). All mice were housed in a pathogen free environment with water and fed mouse breeder diet containing 270 mg Fe/kg (Teklad 8626) provided ad libitum. Mice were euthanized according to AVMA Guidelines for the Euthanasia of Animals. At the German Mouse Clinic, mice were maintained in IVC cages with free access to water and standard mouse chow containing 183 mg Fe/kg (Altromin no.1324) according to the GMC housing conditions and German laws. All tests performed at the GMC were approved by the responsible authority of the Regierung von Oberbayern.

### Immunoblot analysis and RNA electrophoretic mobility shift assay (RNA-EMSA)

Tissues were homogenized in lysis buffer (150 mM NaCl, 10 mM EDTA, 10 mM Tris-HCl pH 7.4, 1% Triton X-100, 1 mM DTT and Cocktail Protease Inhibitor (Roche)) and whole-cell lysates were prepared. Protein concentration was determined using Coomassie Plus Protein Assay Reagent (Thermo Scientific). Lysates were analyzed by NuPAGE 4–12% Bis-Tris Gel with MES SDS-running buffer (Invitrogen). Proteins were transferred to a Hybond-ECL nitrocellulose membrane (Amersham) and probed with the following antibodies: chicken anti-Irp1 polyclonal antibody [27]; rabbit anti-Irp2 antibody [28]; rabbit anti-ferritin-L (Ftl1) antibody (Santa Cruz); TfR1 monoclonal antibody (Zymed); β-actin monoclonal antibody (Calbiochem). Horseradish peroxidase-conjugated secondary antibodies were bound and proteins were visualized using Western Lighting Chemiluminescence Reagent Plus (PerkinElmer Life Sciences). RNA-EMSA was performed by incubating tissue lysate (12 ug) isolated from *Irp2^−/−^* and WT mice with a ^32^P-labeled ferritin-L IRE probe (pGL-66 linearized with *Sma*1) followed by analysis of the RNA-protein complexes on nondenaturing 5% polyacrylamide gels according to Leibold and Munro [29]. The gels were dried and exposed to a PhosphorImager screen for analysis.

### Tissue iron content

Tissue iron content was determined by digesting tissue (20–30 mg) in 40% metal-free nitric acid at 95°C. Samples were diluted in water and analyzed by PerkinElmer Optima 3100XL ICP-OES Spectrometer.

### Immunohistochemistry

Male WT mice at 68–78 weeks old (n = 8) and *Irp2^−/−^* mice at 52–73 weeks old (n = 10) were perfused with Wash Buffer (0.8% NaCl, 0.4% dextrose, 0.8% sucrose, 0.023% CaCl_2_, and 0.034% sodium cacodylate) until liver was cleared, and then fixed with Fix Buffer (4% paraformaldehyde, 4% sucrose, and 1.4% sodium cacodylate, pH 7.4) by cardiac perfusion. Heads were amputated and stored overnight in Fix buffer. Brains were removed and stored in Cacodylate buffer (14.3% sodium cacodylate, pH 7.4) before all 18 brains were embedded together in a solid block matrix using Multi-Brain Technology (NeuroScience Associates (NSA), Knoxville, TN). Cryostat sections (35 um) were stained with 3,3′-diaminobenzidine (DAB)-enhanced Perls' iron stain to detect ferric iron. Whole brain images were viewed with a Zeiss Stemi SV6 microscope and captured with a MTI 3CCD camera. Perls' stained brain sections were imaged with the EVOS-FL imaging system (Life Technologies). Quantification of Perls' stain in CA1 pyramidal cells and Purkinje cells was carried out by measuring staining intensity in a defined subsection of brain sections from *Irp2^−/−^* (n = 10) and WT (n = 8) mice using Image J software.

### Immunofluorescence

For immunofluorescence, cryostat sections were mounted on slides and incubated in a boiled citrate-based antigen retrieval solution (10 mM citrate pH 6.0, 0.05% Tween-20) until cooled (20 min). Sections were blocked using 5% goat serum in Tris-buffered saline plus 0.3% Triton X-100 for 1 h. Double immunofluorescence staining was performed by costaining sections with rabbit anti-rat ferritin antibody (UT106, generated in our laboratory against rat-H- and -L liver ferritin) overnight at 4°C and the following antibodies: mouse anti-NeuN for neuronal nuclei (1∶500, MAB377, Millipore) and mouse calbindin for Purkinje cells (1∶1000, Sigma). Sections were incubated with secondary antibodies Alexa Fluor 488 goat anti-rabbit IgG and Alexa Fluor 594 goat anti-mouse for 1 h at room temperature. Images were viewed with an Olympus IX81 microscope and captured with a DP71 camera.

### Quantitative RT-PCR (qRT-PCR)

Total RNA was extracted from 9.5–10.5 day embryos using TRIzol reagent (Invitrogen). cDNA synthesis was performed with total RNA (200 ng) using Super SuperScript III First -Strand synthesis SuperMix for qRT-PCR (Invitrogen). qRT-PCR was performed using TaqMan assay probes (Table S6) on an Applied Biosystems 7900HT Sequence Detection System. All experiments were performed using 8–10 mice/genotype with each sample assayed in triplicate. The fold change for each gene was calculated using the ▵▵Ct method normalized to Actin with data represented as average ± SEM. Statistical significance was determined using a Student's *t*-test.

### Hematology and clinical chemistry

Blood samples were collected under isoflurane anesthesia by retrobulbar puncture. Blood samples were divided in two portions collected Li-heparin-coated sample tubes (Kabe, Nümbrecht-Elsenroth, Germany) for clinical chemistry of plasma samples and EDTA-coated sample tubes (Kabe, Nümbrecht-Elsenroth, Germany) for hematological analyses, respectively.

EDTA-blood samples were analyzed for the complete blood count using an abc-animal blood counter (Scil animals care company, Viernheim, Germany) using predefined settings for C57BL/6 mice. Number and size of red blood cells, white blood cells, and platelets were measured by electrical impedance and hemoglobin by spectrophotometry. Mean corpuscular volume (MCV), mean platelet volume (MPV) and red blood cell distribution width (RDW) were calculated directly from the cell volume measurements. The hematocrit (HCT) was assessed by multiplying the MCV with the red blood cell count. Mean corpuscular hemoglobin (MCH) and mean corpuscular hemoglobin concentrations (MCHC) were calculated from hemoglobin/red blood cell count (MCH) and hemoglobin/hematocrit (MCHC), respectively. Li-Heparin plasma was separated from cells by centrifugation within two hours after collection. Samples were diluted 1∶2 with deionized water and analyzed for 24 clinical-chemical parameters using an AU400 autoanalyzer (Olympus Germany, Hamburg, Germany) and reagents for human samples provided Olympus Germany (Hamburg, Germany) or Wako Chemicals GmbH (Neuss, Germany) in case of non-esterified fatty acid concentrations (NEFA). Parameters measured included plasma iron concentration, ferritin levels, transferrin concentration and unsaturated iron binding capacity (UIBC). Transferrin saturation was calculated from iron and UIBC values as percentage of iron level on total iron binding capacity (TIBC  =  iron+UIBC). Data were statistically analyzed with the level of significance set at *p*<0.05 by pair-wise comparisons of the means by Welsh-Student's *t*-test.

### Protoporphyrin IX (PPIX) analysis

Tissue samples were collected in 150 µL water, sonicated, and then an equal volume of 3 M HCl was added for a final concentration of 1.5 M HCl. Acidified samples were then incubated for 1 h at 37°C, spun at 13,000 RPM for 10 min, and the supernatant quantified by HPLC using the method for regular porphyrins [30].

### Modified Hole Board (mHB)

The mHB experiments were carried out as previously described [31], [32]. In brief, for each trial an unfamiliar (a blue plastic tube lid, diameter 2 cm, height 1 cm) and a copy of a familiar object (metal cube, diameter 2 cm, height 1.5 cm; remaining for 48 h in the home cage, removed 24 h before testing) were placed into the same corner of the arena at a distance of 2 cm. Each mouse was placed at the start position in the same corner diametrical to the corner where the two objects were placed, facing the board diagonally and was tested for 5 min in moderate light conditions (150 lux in the corners to 200 lux in the middle of the test arena). After each trial, the arena was cleaned and disinfected. All trials were videotaped and tracked by Ethovision 2.3 (Noldus, Wageningen, NL) for calculation of horizontal locomotor activity parameters. The movement detection threshold was set at a shift of the center of gravity of the animal for at least 1 cm in a horizontal direction. A hand-held computer was used by a trained observer to assess line crossings, board entries, rearings on board, rearings in the box, hole exploration, familiar and unfamiliar object exploration, immobility, stretched attends (i.e. risk assessment behaviour), defecation and grooming. Data were analysed by use of the Observer software 4.1 (Noldus, Wageningen, NL) with respect to frequency, latency of first occurrence and duration in % of total observation time. Any behaviour that did not occur within the 5 min observation time was given the maximal latency of 300 s. The object index was calculated as total investigation time (s) at the unfamiliar object divided by the sum of the total investigation time (s) at both objects.

### Neurological testing

Neurological analysis was performed as previously described [33]. Grip strength was measured by measuring maximal force the mice applied to grid attached a force-meter (Bioseb, France) in three consecutive trials. Fore paws and combined fore and hind paw measurements were performed by allowing the mice to grasp the grid before being slowly pulled away. Motor coordination was evaluated by the latencies the animals stayed on a rotating rod, acceleration from 4 to 40 rpm in five minutes. Mice were tested four times with 15 minutes in between. For statistical analysis linear models were used also considering body mass variations.

### Nociception methods

In nociception, 18 *Irp2^−/−^* mice (9 males, 9 females) and 20 WT animals (10 males and 10 females) were screened using a hot-plate assay. A mouse was placed on a 28 cm diameter metal surface maintained at 52+0.2°C surrounded by a 20 cm high Plexiglass wall (TSE, Bad Homburg, Germany). Mice remained for 30 seconds on the plate or until they performed one of three behaviors regarded as indicative of nociception: hind paw licking, hind paw shake/flutter or jumping [34]. The latency of the first sign of pain was compared for sex and genotype using a factorial ANOVA.

### Transmission electron microscopy

Animals (a subset of 5 *Irp2KO* and 4 WT mice) were transcardially perfused with 50 ml ice cold PBS, prior to perfusion with 70 ml of fixing solution (2.5% PFA, 2.5% glutaraldehyde in PBS). Different brain regions were removed and cut into 1 mm^3^ cubes, which were postfixed in 2.5% glutaraldehyde containing 0.1 M sodium cacodylate buffer (pH 7.4) at 4°C overnight. Tissues were fixed in 2.5% electron microscopy grade glutaraldehyde in 0.1 M sodium cacodylate buffer pH 7.4 (Science Services, Munich, Germany), postfixed in 2% aqueous osmium tetraoxide, dehydrated in gradual ethanol (30–100%) and propylene oxide, embedded in Epon (Merck, Darmstadt, Germany) and baked for 24 hours at 60°C. Semi thin sections were cut and stained with toluidine blue. Ultrathin sections of 50 nm were collected onto 200 mesh copper grids, contrasted with uranyl acetate and lead citrate before examination by transmission electron microscopy (Zeiss EM 10 CR electron microscope, Carl Zeiss NTS GmbH, Oberkochen, Germany).

## Results and Discussion

### Phenotypic characterization of *Irp2^−/−^* mice

*Irp2^−/−^* mice were generated by inserting a self-excision cassette containing neomycin (*Neo^r^*) linked to Cre-recombinase (*Cre*) into exon 3 of the mouse *Irp2* gene (Figure S1). *Irp2^−/−^* mice were fertile and indistinguishable from wildtype (WT) littermates in weight or appearance (data not shown). *Irp2^−/−^* mice showed mild microcytic anemia characterized by reduced hemoglobin, hematocrit and mean corpuscular volume (Table S1). Transferrin saturation, serum iron levels and total iron binding capacity were unchanged in *Irp2^−/−^* and WT mice while serum ferritin levels were markedly increased in *Irp2^−/−^* mice (WT, 58.1±12.5 ng/ml (n = 9); *Irp2^−/−^*, 218.6±7.3 ng/mL (n = 10), *p*<0.001) (Table S2). *Irp2^−/−^* mice also showed elevated levels of serum and liver protoporphyrin IX (PPIX) and PPIX-containing aggregates in the cystic, hepatic, and common bile ducts (Table S3 and Figure S2). *Irp2^−/−^* mice displayed altered body iron distribution with total iron content increased in duodenum, kidney and liver and decreased in heart while total brain iron content is unchanged (Table 1).

**Table 1:** Tissue iron content of aged male *Irp2^−/−^* and WT mice.

Tissue	*WT* (n = 8)	*Irp2^−/−^* (n = 10)
Brain	1.98±0.08	1.84±0.07
Duodenum	6.01±1.68	15.41±1.76*
Liver	2.77±0.21	5.81±0.39***
Spleen	15.39±2.44	12.54±1.09
Heart	5.49±0.05	4.92 ±0.13*
Kidney	2.25±0.10	3.37±0.15***
Lung	3.01±0.40	2.80±0.35

Total iron content was determined by inductively-coupled plasma optical emission spectroscopy (ICP-OES). Statistical analysis performed by Student's paired t-test (*p<0.05; ***p<0.001, mean (µg Fe/mg wet tissue weight) ×100± SEM). Ages of mice: WT, 64–75 weeks; *Irp2^−/−^*, 49–71 weeks.

**Figure 1:**
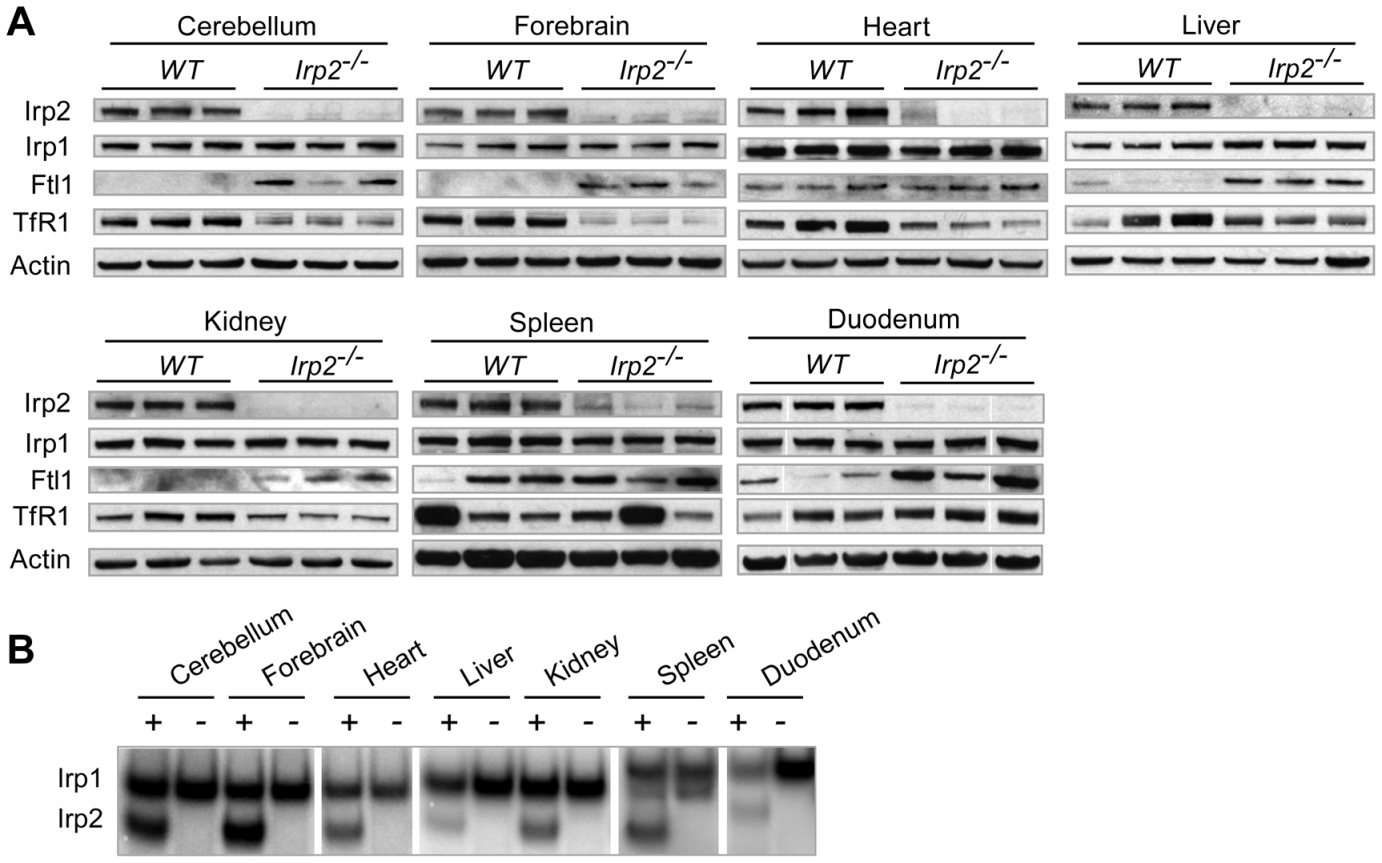
Expression of iron homeostasis proteins in *Irp2^−/−^* and WT mice. *A)* Western blot analysis of tissue extracts from male *Irp2^−/−^* and WT mice (n = 3 mice/genotype) using antibodies to detect Irp2, Irp1, ferritin (Ftl1), TfR1 and β-actin (loading control). A non-specific band migrating near Irp2 is observed in some *Irp2^−/−^* lysates. *B)* Irp1 and *Irp2* RNA-binding activity in lysates was assayed by RNA electrophoretic mobility shift assay using a ^32^P-labeled ferritin IRE as a probe. 0.5% β-mercaptoethanol was added to samples to assay total RNA-binding activity.

Ferritin and TfR1 have been shown to be dysregulated in liver, duodenum and brain in two other distinct *Irp2^−/−^* mouse strains [17], [23], [24]. Similarly, we find increased ferritin-L chain (Ftl1) levels in brain (forebrain and cerebellum), liver, kidney and duodenum and decreased TfR1 levels in brain, heart, liver and kidney in *Irp2^−/−^* mice compared to WT mice (Figure 1A). Ferritin-L and TfR1 levels are similar in *Irp2^−/−^* and WT spleen. Irp2 RNA-binding activity and protein are not detected in *Irp2^−/−^* tissue lysates (Figure 1 A and B). A non-specific band is seen by Western blot analysis that migrates close to Irp2 in some tissue lysates (Figure 1A). Taken together, these phenotypes are in agreement with those described for other *Irp2^−/−^* mouse strains in which Irp2 deficiency causes microcytic anemia, erythropoietic protoporphyria, and altered body iron distribution and expression of ferritin and TfR1 [18], [19], [23].

### Motor coordination/balance, locomotion and nociception are impaired in *Irp2^−/−^* mice

The *Irp2^−/−^* strain generated by LaVaute et al. [17], [35] developed an adult-onset neurodegenerative movement disorder at >6 months of age characterized by tremors, hind-limb weakness, subtle kyphosis and abnormal gait. These mice displayed poor self-grooming activity, and impaired muscular strength and motor coordination when assayed by the hang test and rotarod [17], [35]. In contrast, *Irp2^−/−^* mice generated by Galy et al. [19] showed no overt signs of neurodegeneration or impaired muscle strength at 13–14 months of age. These mice displayed reduced self-grooming activity consistent with a tendency toward reduced rearings (vertical locomotor activity) and impaired motor coordination/balance when challenged by the modified-Hole Board and rotarod tests, respectively [26].

**Figure 2:**
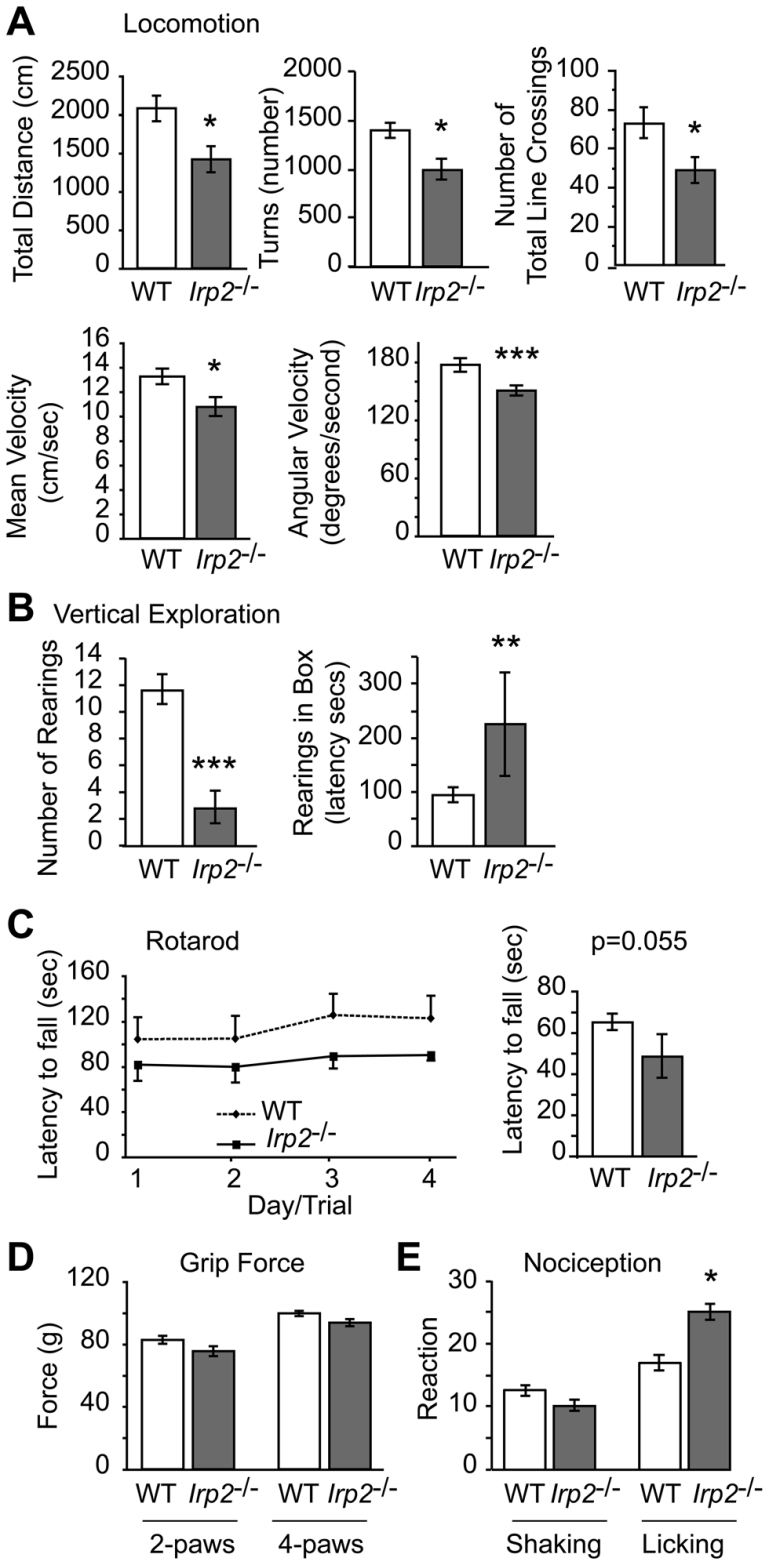
Locomotion, motor coordination and nociception are impaired in *Irp2^−/−^* mice. *Irp2^−/−^* mice display reduced horizontal locomotor activity (total distance traveled, number of turns, number of total line crossings, mean velocity and angular velocity), and *B*) reduced vertical exploratory activity (number of rearing and rearing latency) assessed by the modified-Hole Board test [31]. *C) Left panel,* performance of *Irp2^−/−^* and WT mice on the accelerating rotarod in four trials on four consecutive trials with 15 min inter-trial-interval; *right panel*, decreased mean latency of *Irp2^−/−^* mice to fall off the rotarod (n = 4 trial; *p* = 0.055). *D)* 4-paw grip force test shows no difference in muscular strength between *Irp2^−/−^* and WT mice. *E)* Hot plate test shows increased hind paw licking in *Irp2^−/−^* mice. Data are given as the mean ± SEM; *p<0.05; ^**^ *p*<0.01, ***p<0.001, relative to WT; WT (n = 9) and *Irp2^−/−^* (n = 10).

As our *Irp2^−/−^* mice at 45–63 weeks old did not display tremors, kyphosis, or abnormal gait, we performed a battery of tests to assess behavioral and neurological function. We used the modified-Hole Board test to assay locomotor and exploratory activities, arousal, memory and social affinity, the modified SHIRPA test to assess neurological function, the grip strength test to quantify muscular strength and the accelerating rotarod to measure motor coordination and balance (Tables S4–S5). *Irp2^−/−^* mice displayed an overall reduction in forward locomotion, speed of movement and reduced vertical exploratory activities (rearing frequency and latency) (Figure 2A and B). Rotarod performance was slighty impaired in aged *Irp2^−/−^* mice compared to WT mice, which was not observed in 20-week old *Irp2^−/−^* mice, suggesting a progressive phenotype (Figure 2C and data not shown). The grip strength test (2-paws and 4-paws) revealed that muscular strength was not impaired in *Irp2^−/−^* mice (Figure 2D).

Nociception was also assessed in *Irp2^−/−^* and WT mice. *Irp2^−/−^* mice showed no significant difference in the first pain reaction (hind paw shaking) since they present with the same reaction latency to heat stimuli as WT mice (Figure 2E). However, *Irp2^−/−^* mice displayed a significantly longer reaction latency in the second pain reaction (hind paw licking) (Figure 2E). This finding indicated that *Irp2^−/−^* mice tolerated heat better than WT mice when it was used as noxious stimulus. Increased hind paw licking was also observed in 20-week old *Irp2^−/−^* mice, indicating that this reaction was independent of age (data not shown). Retarded reaction to thermal stimuli on the hotplate could be a secondary effect of reduced motor abilities. If impaired motor abilities influenced the pain reaction, however, the same effect in both reactions, or at least in the first reaction and not in the second reaction, would be observed. Altered nociception heat tolerance was not reported for other *Irp2^−/−^* mice.

Impaired motor coordination and balance are consistent with the *Irp2^−/−^* neurological phenotypes reported by Galy et al. [26] and LaVaute et al. [17], although *Irp2^−/−^* mice generated by Galy et al. [26] did not display horizontal locomotor impairments that we observed in our *Irp2^−/−^* mice. Taken together, our data show that Irp2 deficiency is associated with mildly impaired horizontal locomotion, exploration, motor coordination/balance and nociceptive heat tolerance.

### Iron accumulation in oligodendrocytes and axons in the brain of *Irp2^−/−^* mice

Marked ferric iron accumulation was observed in oligodendrocyte soma and white matter axons in aged *Irp2^−/−^* mice generated by LaVaute et al. [17], [25]. Iron accumulated in the cerebellum, caudate putamen, thalamus, substantia nigra and colliculi, while the frontal cortex, globus pallidus and corpus callosum were not affected [17], [25], [36]. Iron accumulation in axons in cerebellar white matter was associated with axonopathy. Iron was also detected in neuronal cell bodies in gray matter, including the thalamus, colliculi and deep cerebellar neurons, and in Purkinje axons. In contrast, Galy et al. [26] did not detect abnormal brain iron accumulation in their aged *Irp2^−/−^* mice. Both groups analyzed aged mice (12–14 months old) and used DAB-enhanced Perls' iron staining method to detect ferric iron. Both groups also reported no significant differences in total brain iron [23] or total non-heme iron [26] between *Irp2^−/−^* and WT mice.

**Figure 3:**
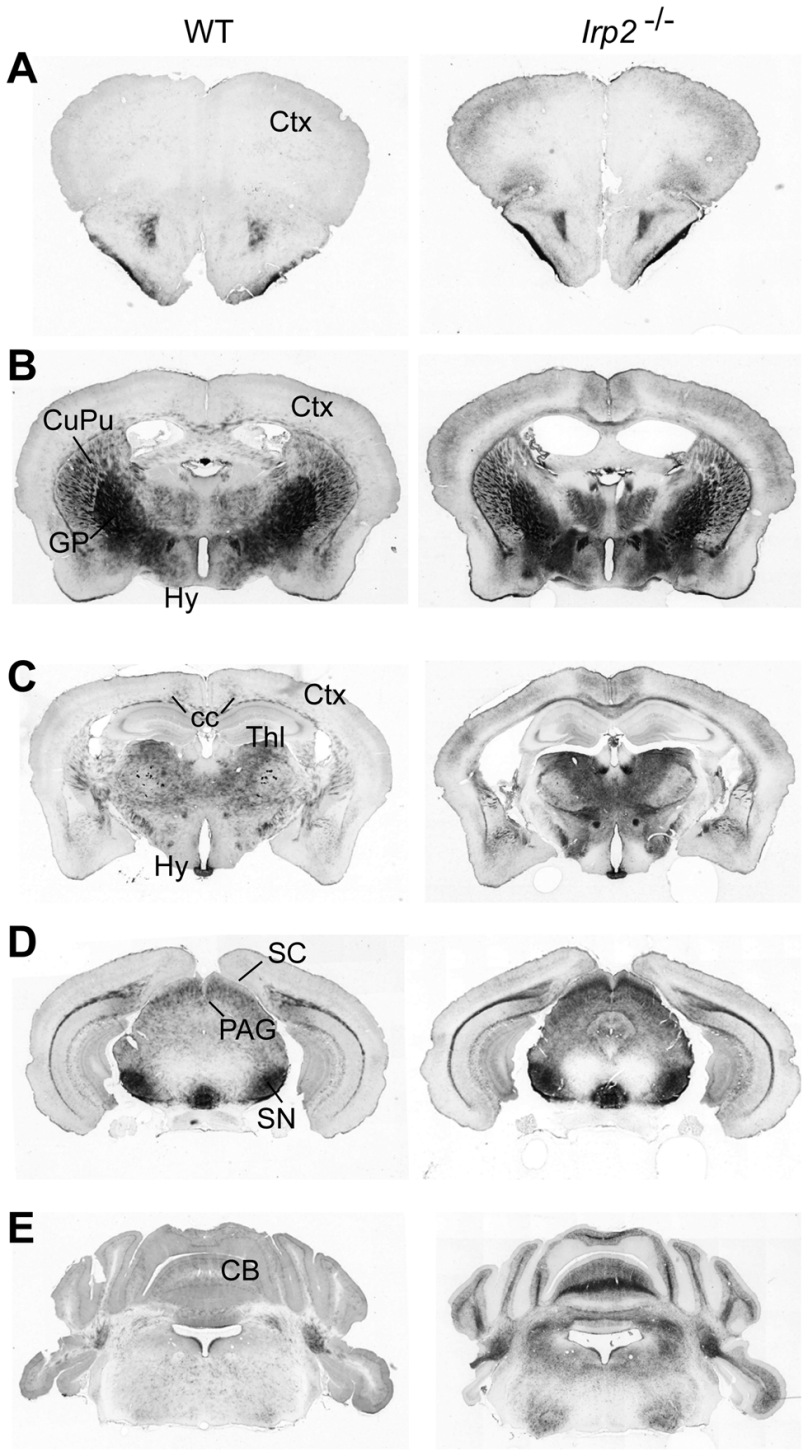
Iron accumulates in specific brain regions of *Irp2^−/−^* mice. Coronal sections from male *Irp2^−/−^* and WT brains from rostral *(top*) to caudal (*bottom*) were selected to show iron accumulation in specific regions of brain. Ferric iron was detected using DAB-enhanced Perls' stain. *Ctx* cortex, *CB* cerebellum, *CPu* caudate putamen, *cc* corpus collosum, *Gp* globus pallidus, *Hy* hypothalamus, *PAG*, periaqueductal gray; *SC* superior colliculus, *SN* substantia nigra and *Thl* thalamus.

**Figure 4:**
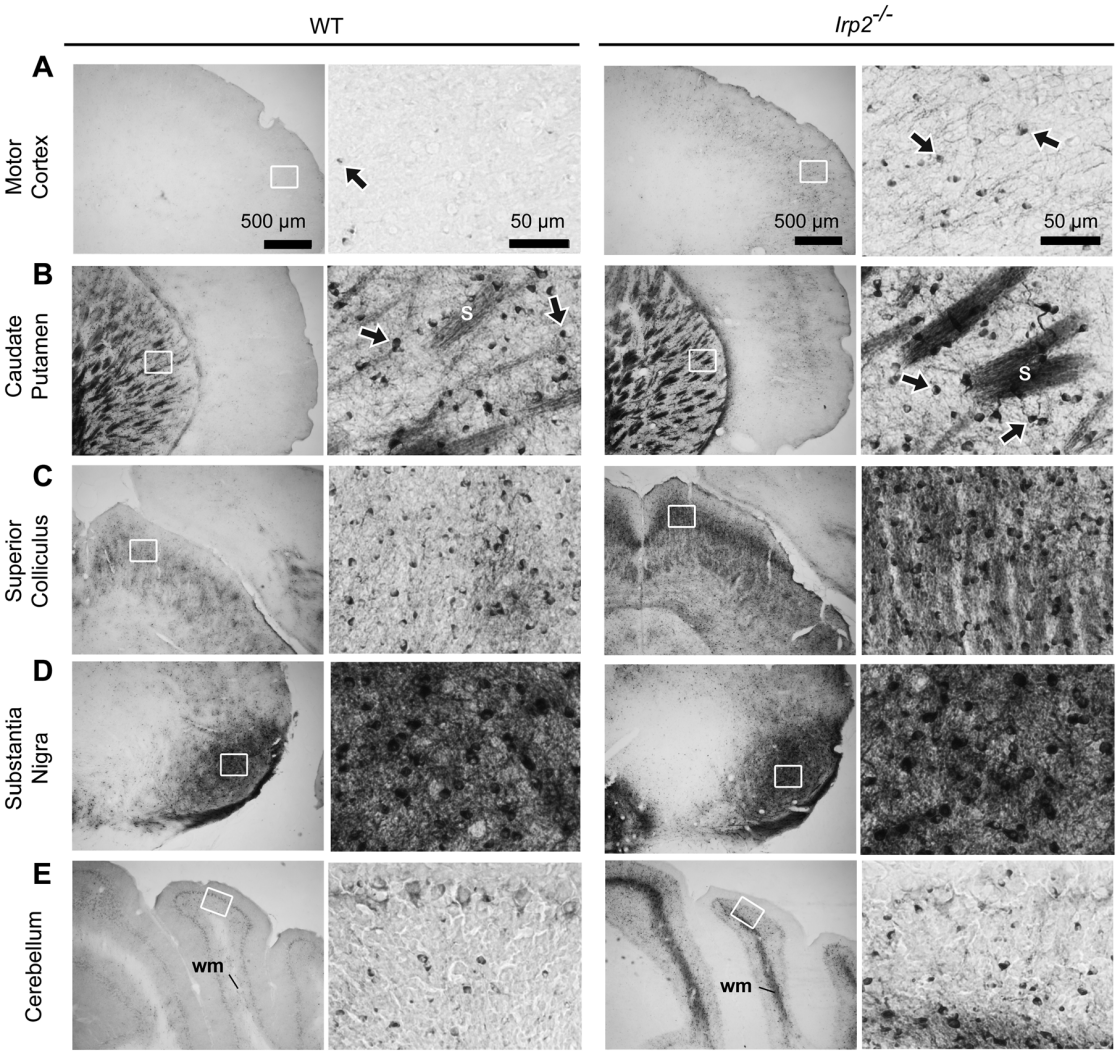
Iron accumulates in axons and oliogodendrocyte cell bodies of *Irp2^−/−^* mice. Increased DAB-enhanced Perls' iron stain is observed in small cells that have an eccentric nucleus characteristic of oliogodendrocyte morphology [37] and in axons in *A)* cerebral cortex, *B)* caudate putamen, *C)* superior colliculus, *D)* substantia nigra, and *E*) cerebellum. *Arrows*, oligodendrocytes; *S*, striosomes; *wm*, white matter; *gl,* granule layer. Scale bars: 50 μm and 500 μm.

Similar to previously reported global *Irp2^−/−^* mice, we found no significant differences in total brain iron content between *Irp2^−/−^* and WT mice using inductively-coupled optical emission spectroscopy (Table 1). We next assessed ferric iron histochemically in serial brain sections prepared from *Irp2^−/−^* (n = 10) and WT (n = 8) mice using DAB-enhanced Perls' stain (Files S1 and S2). Marked iron deposition was observed in cortex, caudate putamen, thalamus, superior colliculus and cerebellum as well as in other brain regions in *Irp2^−/−^* mice (Figure 3 and Files S1 and S2). In cortex, iron accumulated in small cells that have an eccentric nucleus characteristic of oligodendrocyte morphology [37] and in axons (Figure 4A). Iron accumulation was also observed in axon tracts and in oligodendrocyte soma associated with striosomes, as well as in oligodendrocytes scattered throughout the caudate putamen (Figure 4B). Significant iron deposition was observed in the superior colliculus (Figure 4C) and in the cerebellar white matter and oligodendrocytes associated with white matter in *Irp2^−/−^* mice (Figure 4E). Iron deposition in caudate putamen, cerebellar white matter and colliculus, as well as other regions (Files S1 and S2), is in agreement with *Irp2^−/−^* mice generated by LaVaute et al. [17], [25]. Unlike *Irp2^−/−^* mice generated by LaVaute et al. [17], [25], we did not detect increased iron deposition in the substantia nigra of our *Irp2^−/−^* mice (Figure 4D). WT and *Irp2^−/−^* mice used in our study were older than mice used by Smith et al. [25] (<12 months old), and it is possible that the high iron content in the substantia nigra of our WT mice made it difficult to discern iron differences between WT and *Irp2^−/−^* mice. We also found significant iron deposition in the cerebral cortex and corpus collosum of *Irp2^−/−^* mice, which was not observed by LaVaute et al. [17] (Figures 3C and 4A).

### Iron content is reduced in CA1 pyramidal neurons and in Purkinje neurons of *Irp2^−/−^* mice

**Figure 5:**
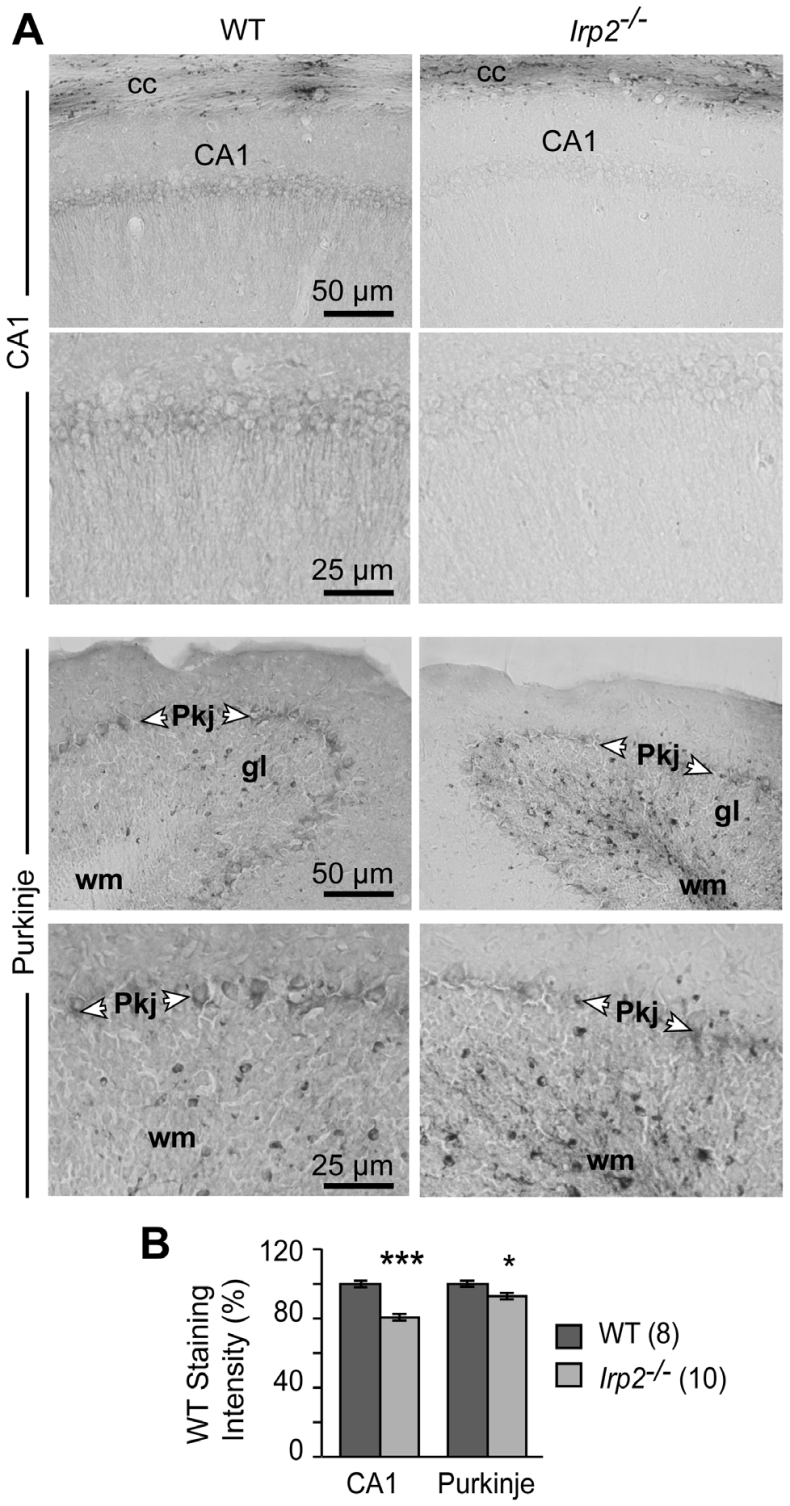
Iron is reduced in Purkinje neurons and in CA1 pyramidal neurons of *Irp2^−/−^* mice. *A)* Perls' DAB-enhanced iron staining in CA1 pyramidal neurons and Purkinje neurons in *Irp2^−/−^* and WT mice (*Pkj*, Purkinje; *gl*, granular layer; *wm*, white matter). *B)* Quantification of Perls' staining of images in *(A)* was carried out by measuring staining intensity in a defined subsection of brains from *Irp2^−/−^* (n = 10) and WT (n = 8) (Figure S3). Data are given as the mean ± SEM; *p<0.05; ***p<0.001 relative to WT. Scale bars: 25 μm and 50 μm.

We next assessed Perls' iron staining in hippocampal CA1 pyramidal neurons and in cerebellar Purkinje neurons in *Irp2^−/−^* and WT mice. Perls' staining was significantly reduced in CA1 pyramidal neuronal soma and in their apical dendrites (Figure 5 and Figure S3). Purkinje neuronal soma in *Irp2^−/−^* mice showed a slight, but significant reduction in iron content compared to WT mice (Figure 5 and Figure S3).

**Figure 6:**
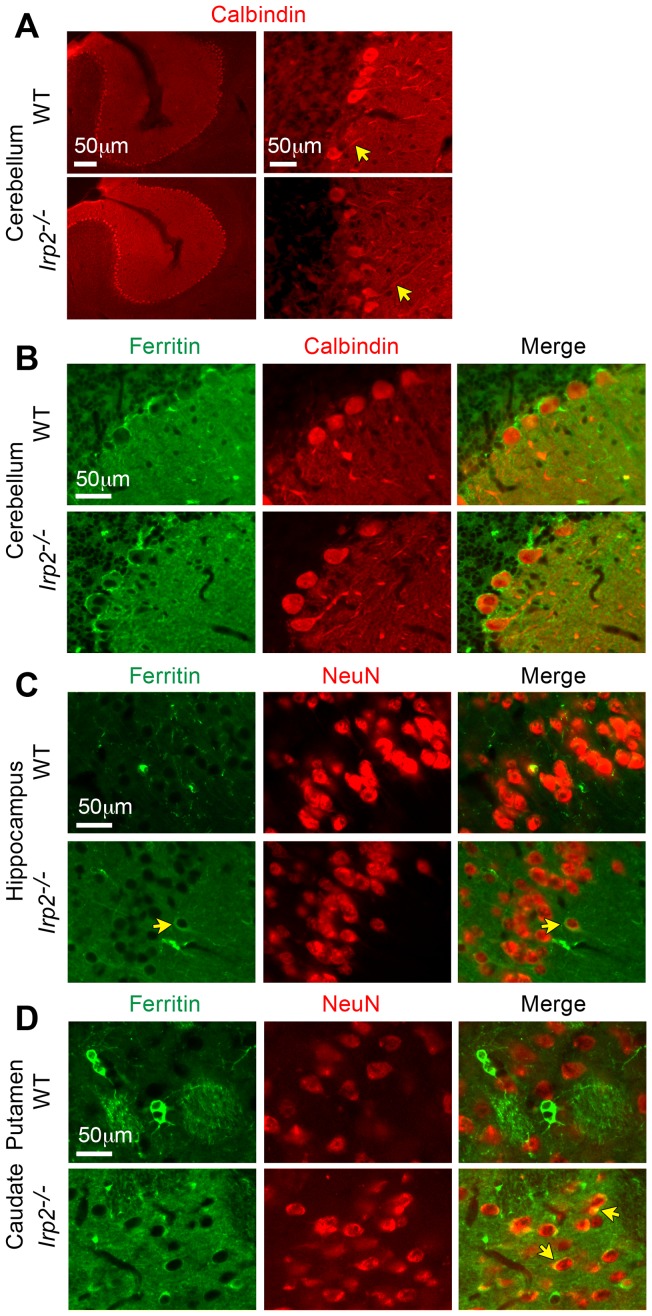
Ferritin expression is increased in *Irp2^−/−^* neurons. Double immunofluorescence labeling of brain sections from male *Irp2^−/−^* and WT mice using ferritin antibody with either calbindin (Purkinje specific) or NeuN (neuronal nuclei) antibodies. *A)* Calbindin immunofluorescence shows that Purkinje cell number and morphology (*yellow arrows*, processes), are normal in *Irp2^−/−^* cerebellum. *B)* Ferritin and calbindin double immunofluorescence of cerebellum shows similar ferritin expression in *Irp2^−/−^* and WT Purkinje neurons. *C)* Ferritin and NeuN double immunofluorescence of hippocampal CA1 pyramidal cell layer, and *D)* caudate putamen shows increased ferritin expression in neuronal cell bodies (*yellow arrows*) of *Irp2^−/−^* mice compared to WT mice. Increased ferritin staining is observed in the neuropil in the hippocampus and caudate putamen of *Irp2^−/−^* mice. Scale bars: 50 μm.

A reported phenotype of *Irp2^−/−^* mice generated by LaVaute et al. [17] is the degeneration and partial loss of Purkinje neurons although this was not found in *Irp2^−/−^* mice generated by Galy et al. [26]. We stained *Irp2^−/−^* and WT cerebellar sections with calbindin antibody (Purkinje cell specific) and found no abnormalities in Purkinje cell morphology or cell number in *Irp2^−/−^* mice (WT, 3.16 cells/inch ±0.267; *Irp2^−/−^*, 3.57 cells/inch ±0.232; *p* = 0.264) (Figure 6A). Ultrastructural analysis of substantia nigra, caudate putamen, cerebellum, cortex, hippocampus and hypothalamus did not reveal pathological alterations in these regions except for the presence of age-related lipofuscin deposits in neurons that are detected in aged WT and *Irp2^−/−^* mice (Figure S4 and data not shown). Myelinization also appeared normal in *Irp2^−/−^* mice evaluated by Luxol Fast Blue staining (Figure S5).

The reduction in Perls' staining in *Irp2^−/−^* CA1 pyramidal and Purkinje neurons suggests that these neurons may be iron deficient. Because neurons acquire iron primarily by TfR1 [38], [39], the reduction in TfR1 abundance in *Irp2^−/−^* cerebellar and forebrain lysates compared to WT is consistent with reduced transferrin-dependent iron uptake (Figure 1A). In contrast to TfR1, ferritin expression is increased in *Irp2^−/−^* cerebellar and forebrain lysates (Figure 1A). We next assessed ferritin expression in neurons in *Irp2^−/−^* and WT sections from cerebellum, caudate putamen and hippocampus by double immunofluorescence using a ferritin antibody in combination with antibodies specific to neurons (NeuN) and to Purkinje neurons (calbindin). Ferritin immunoreactivity was similar in *Irp2^−/−^* and WT Purkinje neurons (Figure 6B), but was increased in *Irp2^−/−^* CA1 pyramidal neurons and in neurons in the caudate putamen (Figure 6C and D). We were not able to assess TfR1 immunostaining in these sections due to technical difficulties. Taken together, these data suggest that the reduction in TfR1 expression and increased ferritin expression in *Irp2^−/−^* mice could lead to reduced iron uptake and increased iron storage, thus causing cellular iron deficiency. While notable neuronal pathology was not observed in *Irp2^−/−^* brain, we suggest that neuronal iron deficiency may cause cellular dysfunction that contributes to the neurological and behavioral deficits in *Irp2^−/−^* mice.

## Conclusions

In conclusion, our data show that mice with a global *Irp2* deficiency recapitulate the main features of other Irp2 deficient mouse models such as microcytic anemia, erythropoietic protoporphyria, altered body iron distribution and altered expression of ferritin and TfR1 in tissues. Our aged *Irp2^−/−^* mice did not exhibit, tremor, ataxia, bradykinesia and postural abnormalities as described by LaVaute et al. [17], but did display mild impairments in horizontal locomotion, rearing activity, balance/motor coordination and nociceptive heat tolerance when assayed by specific tests. Our data are consistent with impaired motor and balance coordination reported for the other two strains of *Irp2^−/−^* mice and with reduced rearing activity reported for *Irp2^−/−^* mice generated by Galy et al. [26]. The reduced rearing activity in our *Irp2^−/−^* mice is consistent with a slight reduction in self-grooming (which could imply a reduction in vertical locomotion), although this did not reach statistical significance, and is similar to other *Irp2^−/−^* mice that exhibited a significant reduction in self-grooming activity [17], [26].

Our *Irp2^−/−^* mice showed distinctive features not reported for other *Irp2^−/−^* mice. The modified hole board test revealed that *Irp2^−/−^* mice displayed an overall reduction in locomotor activity characterized by decreased total distance moved, line crossings, turning frequency and movement velocity. This combination of mild hypoactivity and reduced rearing in our *Irp2^−/−^* mice suggests reduced exploratory motivation. Galy et al. [26] also used the modified hole board test, but did not observe impaired horizontal locomotion in their 13–14 month old *Irp2^−/−^* mice. We also found that *Irp2^−/−^* mice showed a hypoalgesic phenotype as assessed by the hot plate test that was independent of age. Our *Irp2^−/−^* mice develop diabetes (EAL, SN, CPA and KBZ, unpublished observation), and it is possible that hypoalgesia is a consequence of impaired glucose tolerance. Another novel phenotype in our *Irp2^−/−^* mice is the presence of PPIX containing aggregates in the cystic, hepatic, and common bile ducts. Cooperman et al. [23] showed increased synthesis of the heme biosynthetic enzyme erythroid aminolevulinate synthase 2 (Alas2), which contains an IRE in the 5′ untranslated region, and reduced expression of TfR1 in *Irp2^−/−^* erythroid precursor cells. Loss of translational repression of Alas2 by Irp2 deficiency and reduced Tf-dependent iron uptake likely results in elevated PPIX levels we observed in the serum, liver and bile ducts. Whether other *Irp2^−/−^* mice show PPIX aggregates in the bile ducts remains to be determined.

An explanation for the varied neurodegenerative phenotypes in the different *Irp2^−/−^* models is not clear, but it is known that neurological and behavioral phenotypes can be sensitive to genetic background differences and gene targeting strategies [40]–[42]. Our mice were generated using 129/Sv-CP ES cell line and were backcrossed to C57BL/6J mice for five generations, and those of Galy et al. [19], [26] were generated using the 129P2/OlaHsd ES cell line and backcrossed to C57BL/6J mice for three generations. *Irp2^−/−^* mice generated by LaVaute et al. [17] are mixed genetic background consisting of C57BL/6 and B129S4/SVJ. In addition, different targeting approaches were used to generate *Irp2^−/−^* mice. LaVaute et al. [17] generated a global model of Irp2 deficiency by insertion of a PGK-neomycin gene into exon 3/4 of *Irp2* gene. Galy et al. [19] generated conditional alleles of *Irp2* by insertion of a β-Geo cassette flanked by Frt sites into intron 2 of the *Irp2* gene and was co-inserted with LoxP sites flanking exon 3. Cre-mediated excision of exon 3 generated a null allele. Our *Irp2^−/−^* mice were generated by the insertion of a self-excision cassette containing neomycin linked to Cre-recombinase into exon 3 of the mouse *Irp2* gene. Targeting strategies can lead to different phenotypes due to silencing or activation of flanking genes caused by the retention of selection cassettes, generation of truncated gene products with biological activity or inactivation of non-coding RNAs with the targeted locus [43], [44]. The expression of neighboring genes close to the *Irp2* locus was not altered in *Irp2^−/−^* mice [19] and we did not identify annotated non-coding RNAs located within the *Irp2* locus. The differences in neurological and behavioral phenotypes in *Irp2^−/−^* mice could also be affected by environmental conditions (food, altitude or environmental stimulation) [44] and natural variation in iron metabolism in different strains of inbred mice [45]. The reasons for the different neurological and behavioral phenotypes observed in *Irp2^−/−^* mice are not completely understood, but it is likely that the genetic background and the targeting strategies used to generate *Irp2^−/−^* are important components.

Ultrastructural analyses of several regions of brain from *Irp2^−/−^* mice reveal normal neuronal morphology and calbindin staining of cerebellar sections show no loss of Purkinje cells. However, marked iron accumulation is observed in white matter and in oligodendrocytes associated with white matter throughout the brain consistent with studies from LaVaute et al. [17]. Oligodendrocytes normally contain high levels of iron and express high levels of ferritin. The high iron content of oligodendrocytes is thought to be required for myelin production [46]. Myelinization is not affected in *Irp2^−/−^* brain, suggesting that high iron content is not detrimental to oligodendrocyte function.

A notable difference between our *Irp2^−/−^* mice and those of LaVaute et al. [17] is the significant iron accumulation in oligodendroycytes in the cortex of *Irp2^−/−^* mice. We also found that CA1 pyramidal neurons and Purkinje neurons are iron deprived. Since neurons primarily acquire iron by TfR1, reduced TfR1 and increased ferritin expression in *Irp2^−/−^* brain would reduce transferrin-dependent iron uptake in parallel with an increase ferritin iron sequestration, eventually leading to cellular iron deficiency. Although we did not find pathological evidence of neurodegeneration, it is possible that cellular iron deficiency in neurons causes cellular dysfunction that leads to mild deficits in locomotion, motor coordination and nociception. This idea is consistent with a recent study showing that *Irp2^−/−^* mice develop motor neuron disease characterized by increased ferritin and decreased TfR1 expression in motor neurons, reduced spinal cord iron and impaired mitochondrial function [47]. Taken together, these studies suggest that abnormal brain iron metabolism due to Irp2 deficiency disrupts cellular function and leads to mild neurological, behavioral and nociception impairments.

## Supporting Information

Figure S1 **Generation of** ***Irp2^−/−^*** **mice.** (A1) Schematic representation of exons I-VII of the murine *Irp2* (*Ireb2*) gene. Exons (black boxes) and introns (black lines). (A2) Schematic diagram of the targeting vector. A genomic clone (p11.1) containing exons III-VII encoding amino acids 37–233 in Irp2 was isolated from a mouse 129 genomic library. A *Sca*I*-Pst*I fragment from p11.1 (dotted lines) was subcloned into Bluescript SK to generate the targeting vector. A self-excision cassette containing neomycin (*Neo^r^*) linked to Cre-recombinase (*Cre*) was inserted into exon III of the mouse *Irp2* gene [48]. This cassette (pACN) contains the *Cre* gene (driven by the testes-specific angiotensin-converting enzyme (tACE promoter) linked to the *Neo^r^* gene (driven by the polymerase II promoter), and is flanked by loxP sites allowing for excision of *Neo^r^* as it passes through the male germ line. This cassette also contains human *hIrp1-Flag* sequence fused in-frame to exon III of *Irp2* for experiments related to functional replacement of Irp2 with Irp1. For reasons discussed below, Irp1-Flag was not detected in any tissues examined. Thymidine kinase (*Tk1* and *Tk2*) genes were inserted at the 5′ and 3′ ends of the targeting vector to select against random insertion. (A3) Predicted structure of the *Irp2* replacement allele after homologous recombination and germ-line induced self-excision. The targeting vector was electroporated into R1 ES cell line (129/Sv-CP) and heterozygous cells in which a homologous recombination event occurred were identified by PCR and Southern blot analysis of *Sca*I-digested DNA probed with 3′ EXT and Neo probes (Figure S1B, left panels). A targeted ES cell clone (F4) was microinjected into C57BL/6J blastocysts to generate chimeric animals. A chimera was bred to C57BL/6J mice and resulting chimeric progeny was identified by coat color and for the presence of the *Irp2^+/−^* allele by PCR and Southern blotting. Heterozygous offspring were backcrossed five times to the C57BL/6J background and intercrossed to generate homozygous *Irp2^−/−^* mice. (B) Germline transmission of the *Neo*-deleted allele was confirmed by Southern blot analysis of *Sca*1-digested genomic DNA from *Irp2^+/+^*, *Irp2^+/−^* and *Irp2^−/−^* mice probed with the 3′ EXT probe and shows a WT band (10 kb) in *Irp2^+/+^*, a WT and a mutant band (7 kb) in *Irp2^+/−^* and a mutant band in *Irp2^−/−^* (7 kb). The absence of the *Neo^r^/Cre* cassette was confirmed by probing the blot with a Neo probe (not shown). PCR genotyping was performed using tail DNA biopsies (Table S9). (C) Quantitative RT-PCR showed *Irp2* mRNA was not detected in *Irp2^−/−^* E9.5-E10.5 lysates and reduced 2-fold in *Irp2^+/−^* lysates compared to WT lysates (TaqMan assay: *Irp2* exons 3–4). *hIrp1-Flag* mRNA was not expressed in *Irp2^+/+^* lysates, but is expressed in *Irp2^+/−^* and *Irp2^−/−^* lysates (TaqMan assay: *hIrp1*). A TaqMan assay corresponding to exons 1–2 (common to both *Irp2* and *hIrp1-Flag*) showed that *hIrp1-Flag* mRNA expression is ∼ 80% of endogenous *Irp2* mRNA. (D) Although *hIrp1-Flag* mRNA was detected in *Irp2^−/−^* lysates, no hIrp1-Flag protein or RNA-binding activity was detected by Western blot analysis or by RNA-EMSA, respectively, in E9.5-E10.5 lysates or any tissue examined (data not shown). The lack of hIrp1-Flag expression is likely due to faulty translation and/or increased turnover of the hIrp1-Flag protein. If hIrp1-Flag is expressed at low levels, the RNA-binding activity of this recombinant protein is not sufficient to compensate for loss of Irp2 protein since *Irp2^−/−^* mice display altered body iron distribution (Table 1), microcytic anemia (Table S1), and inappropriate regulation of Irp2 targets ferritin and TfR1 (Figure 1) in agreement with published phenotypes of *Irp2^−/−^* mice [17], [23], [24]. TaqMan assays used in these experiments can be found in Table S6.
(TIF)

Figure S2 **Presence of PPIX-containing granules in the common bile duct of** ***Irp2^−/−^*** **mice.** Black arrows indicate PPIX-containing granules in *Irp2^−/−^* (C) mice. B and D are magnified images of A and C. PPIX-containing granules are found in all male and female *Irp2^−/−^* mice examined at 2.5- to 18-months of age.
(TIF)

Figure S3 **DAB-enhanced Perls' iron staining of hippocampus and cerebellum of aged** ***Irp2^−/−^*** **and WT mice.** Coronal sections of hippocampus (A) (slide 41) and cerebellum (B) (slide 58) used for quantification of iron in CA1 pyramidal neurons and in Purkinje neurons in Figure 5. Dotted boxes indicate the WT (6B-4) and the *Irp2^−/−^* (6A-3) mice used for DAB-enhanced Perls' staining in File S1 and File S2. Ages of male mice: WT, 65–71 weeks; *Irp2^−/−^*, 46–53 weeks.
(TIF)

Figure S4 **Ultrastructual analysis of brain regions of aged** ***Irp2^−/−^*** **and WT mice shows no evidence of neurodegeneration.** Electron micrographs of the caudate putamen, substantia nigra and cerebellum of *Irp2^−/−^* and WT mice. Myelinized (white arrows) and non-myelinized (black arrow) axons are shown. No pathological alterations were evident in these brain regions. Similar amounts of lipofuscin deposits (L) were detected in both *Irp2^−/−^* and WT mice. This can be accounted as a normal finding since lipofuscin accumulates with age in many organs including brain. Representative images are shown from WT (n = 4) and *Irp2^−/−^* (n = 5) mice. Ages of male mice: WT, 65–71 weeks; *Irp2^−/−^* 46–53 weeks. Scale bars are indicated.
(TIF)

Figure S5 **Luxol Fast Blue staining of brains of aged** ***Irp2^−/−^*** **and WT mice shows no difference in myelinization.** Representative images are shown from WT (n = 4) and *Irp2^−/−^* (n = 5) mice. Ages of male mice: WT, 65–71 weeks; *Irp2^−/−^*, 46–53 weeks.
(TIF)

Table S1 **Hematological parameters of aged male WT and** ***Irp2^−/−^*** **mice.**
(DOCX)

Table S2 **Serum iron parameters of aged male WT and** ***Irp2^−/−^*** **mice.**
(DOCX)

Table S3 **Protoporphyrin IX (PPIX) levels of 12-month old male WT and** ***Irp2^−/−^*** **mice.**
(DOCX)

Table S4 **Results of behavioral observation in the modified-Hole Board test.**
(DOCX)

Table S5 **Video-tracking results regarding locomotor behavior.**
(DOCX)

Table S6 **List of primers and TaqMan assays used for genotyping and qRT-PCR.**
(DOCX)

File S1 **DAB-enhanced Perls' iron staining of coronal sections of WT (6B-4) brains.** Images (10–66) are from rostral to caudal. High resolution files are available from the corresponding author.
(ZIP)

File S2 **DAB-enhanced Perls' iron staining of coronal sections of** ***Irp2^−/−^*** **(6A-3) brains.** Images (10–66) are from rostral to caudal. High resolution files are available from the corresponding author.
(ZIP)
