# Supplementary figures and images for: Abnormal Brain Iron Metabolism in Irp2 Deficient Mice Is Associated with Mild Neurological and Behavioral Impairments (part 1 of 2)
Source: PLoS One. 2014 Jun 4;9(6):e98072. doi: 10.1371/journal.pone.0098072 (PMC4045679; doi:10.1371/journal.pone.0098072)

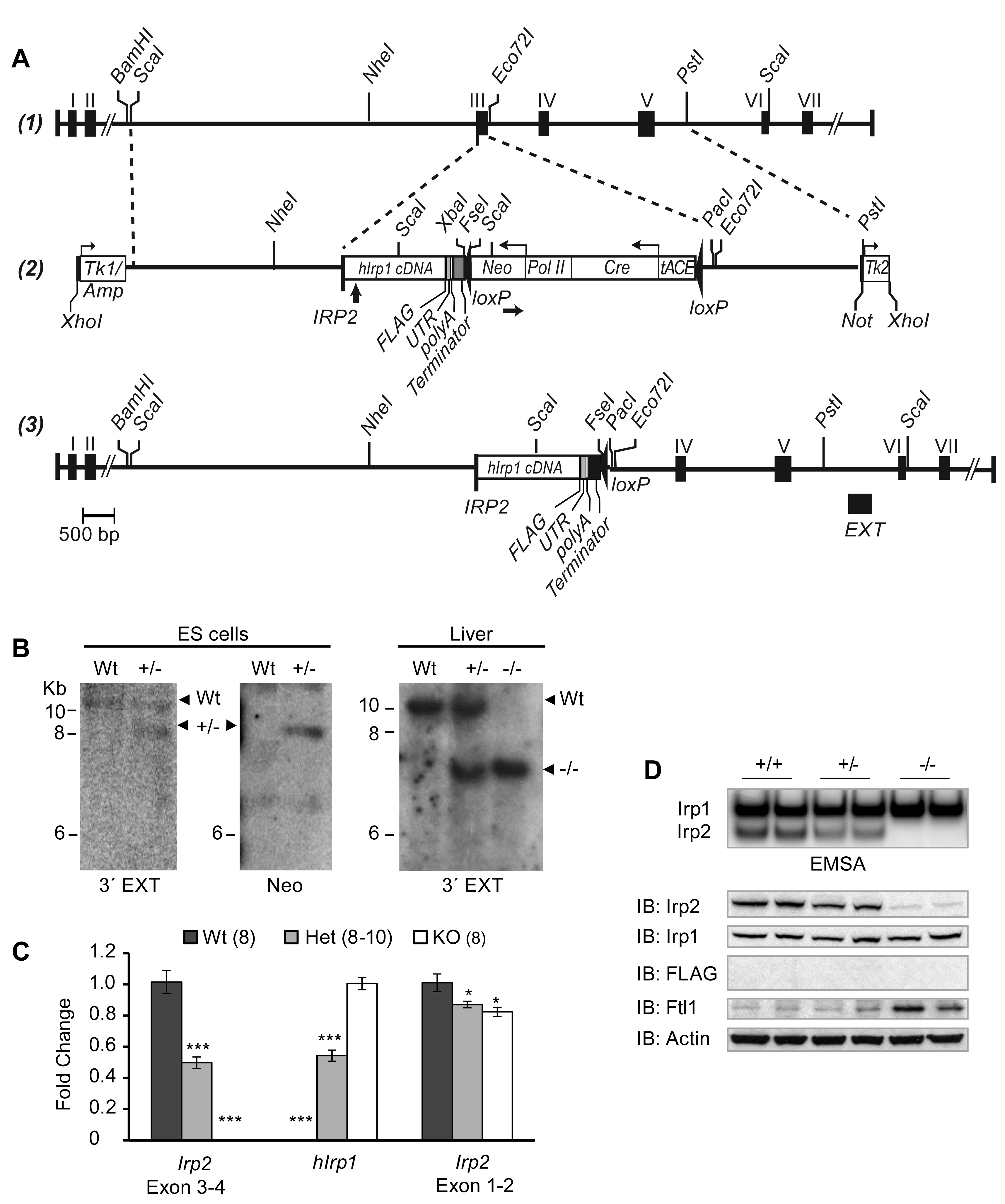

Supplement: Figure S1 — Generation of Irp2−/− mice. (A1) Schematic representation of exons I-VII of the murine Irp2 (Ireb2) gene. Exons (black boxes) and introns (black lines). (A2) Schematic diagram of the targeting vector. A genomic clone (p11.1) containing exons III-VII encoding amino acids 37–233 in Irp2 was isolated from a mouse 129 genomic library. A ScaI-PstI fragment from p11.1 (dotted lines) was subcloned into Bluescript SK to generate the targeting vector. A self-excision cassette containing neomycin (Neor) linked to Cre-recombinase (Cre) was inserted into exon III of the mouse Irp2 gene [48]. This cassette (pACN) contains the Cre gene (driven by the testes-specific angiotensin-converting enzyme (tACE promoter) linked to the Neor gene (driven by the polymerase II promoter), and is flanked by loxP sites allowing for excision of Neor as it passes through the male germ line. This cassette also contains human hIrp1-Flag sequence fused in-frame to exon III of Irp2 for experiments related to functional replacement of Irp2 with Irp1. For reasons discussed below, Irp1-Flag was not detected in any tissues examined. Thymidine kinase (Tk1 and Tk2) genes were inserted at the 5′ and 3′ ends of the targeting vector to select against random insertion. (A3) Predicted structure of the Irp2 replacement allele after homologous recombination and germ-line induced self-excision. The targeting vector was electroporated into R1 ES cell line (129/Sv-CP) and heterozygous cells in which a homologous recombination event occurred were identified by PCR and Southern blot analysis of ScaI-digested DNA probed with 3′ EXT and Neo probes (Figure S1B, left panels). A targeted ES cell clone (F4) was microinjected into C57BL/6J blastocysts to generate chimeric animals. A chimera was bred to C57BL/6J mice and resulting chimeric progeny was identified by coat color and for the presence of the Irp2+/− allele by PCR and Southern blotting. Heterozygous offspring were backcrossed five times to the C57BL/6J background and [file pone.0098072.s001.tif]

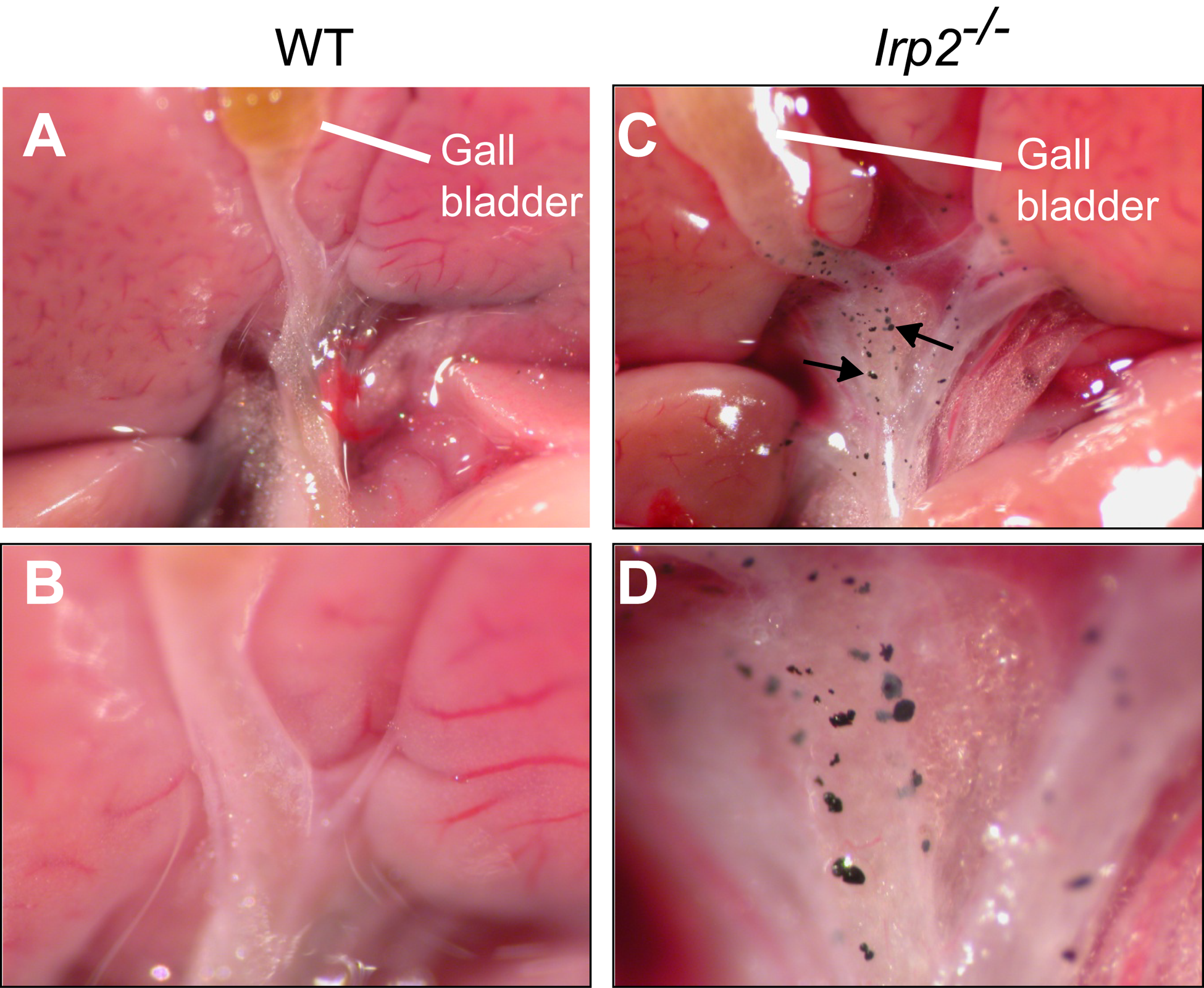

Supplement: Figure S2 — Presence of PPIX-containing granules in the common bile duct of Irp2−/− mice. Black arrows indicate PPIX-containing granules in Irp2−/− (C) mice. B and D are magnified images of A and C. PPIX-containing granules are found in all male and female Irp2−/− mice examined at 2.5- to 18-months of age. (TIF) [file pone.0098072.s002.tif]

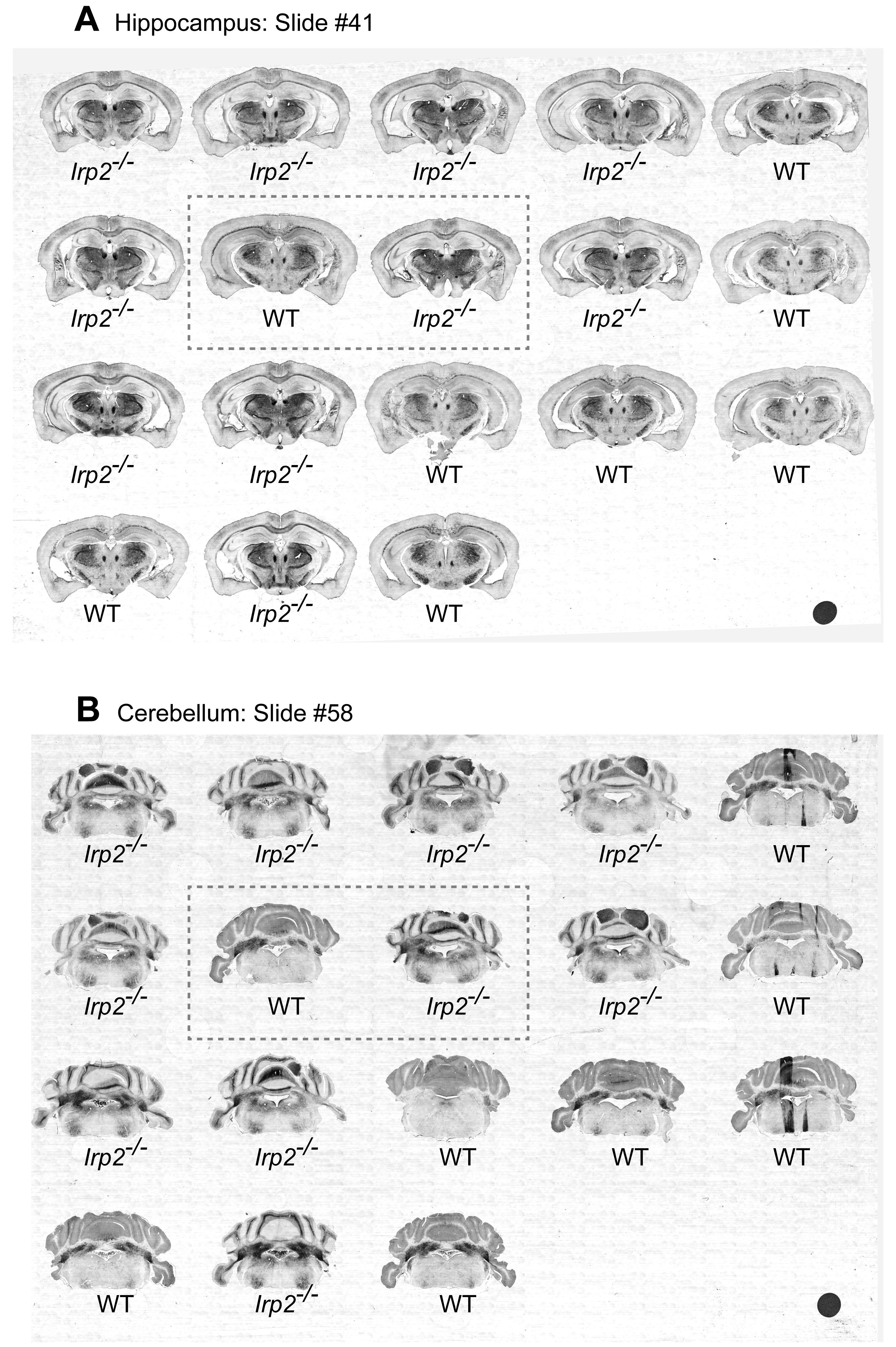

Supplement: Figure S3 — DAB-enhanced Perls' iron staining of hippocampus and cerebellum of aged Irp2−/− and WT mice. Coronal sections of hippocampus (A) (slide 41) and cerebellum (B) (slide 58) used for quantification of iron in CA1 pyramidal neurons and in Purkinje neurons in Figure 5. Dotted boxes indicate the WT (6B-4) and the Irp2−/− (6A-3) mice used for DAB-enhanced Perls' staining in File S1 and File S2. Ages of male mice: WT, 65–71 weeks; Irp2−/−, 46–53 weeks. (TIF) [file pone.0098072.s003.tif]

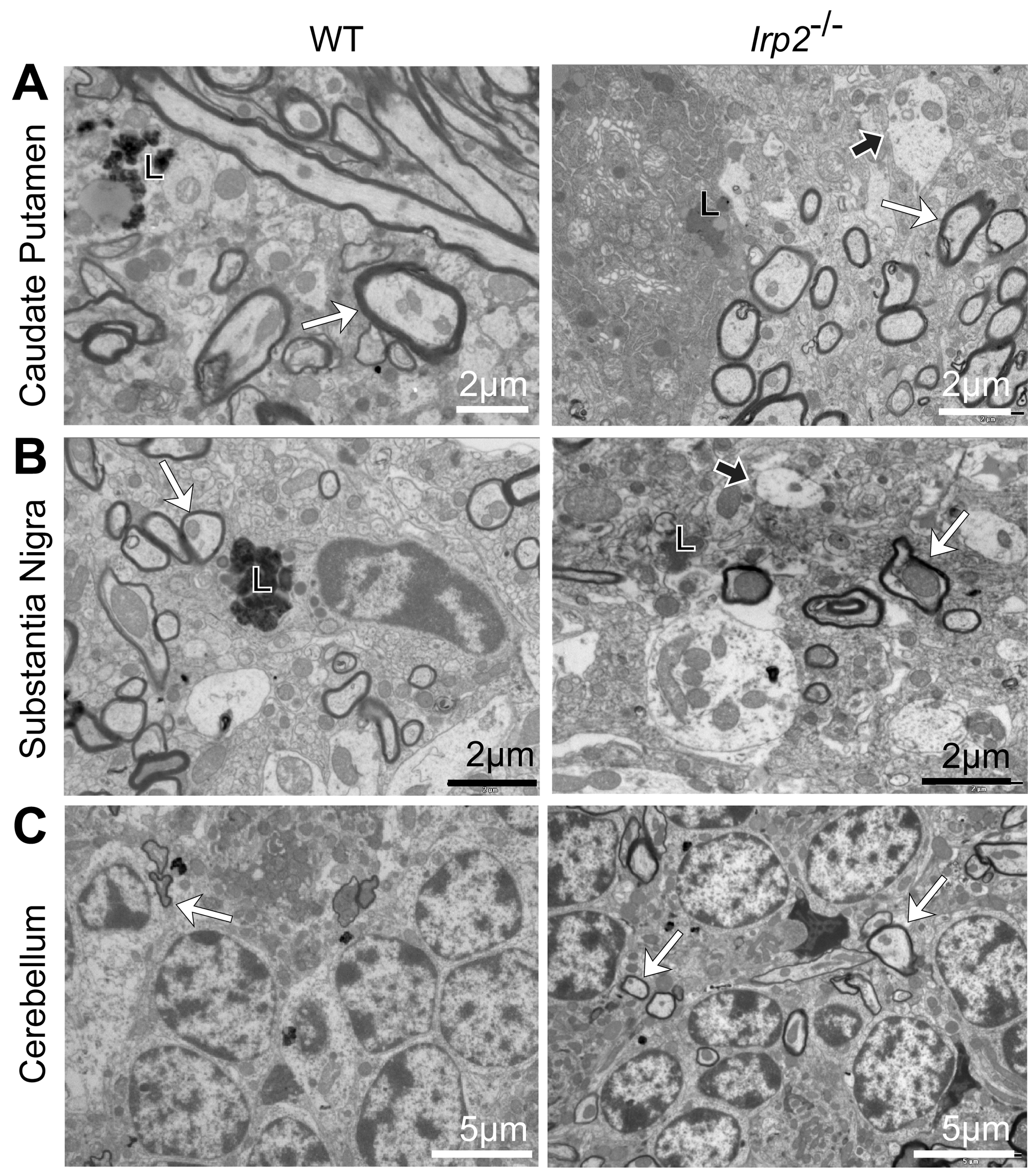

Supplement: Figure S4 — Ultrastructual analysis of brain regions of aged Irp2−/− and WT mice shows no evidence of neurodegeneration. Electron micrographs of the caudate putamen, substantia nigra and cerebellum of Irp2−/− and WT mice. Myelinized (white arrows) and non-myelinized (black arrow) axons are shown. No pathological alterations were evident in these brain regions. Similar amounts of lipofuscin deposits (L) were detected in both Irp2−/− and WT mice. This can be accounted as a normal finding since lipofuscin accumulates with age in many organs including brain. Representative images are shown from WT (n = 4) and Irp2−/− (n = 5) mice. Ages of male mice: WT, 65–71 weeks; Irp2−/− 46–53 weeks. Scale bars are indicated. (TIF) [file pone.0098072.s004.tif]

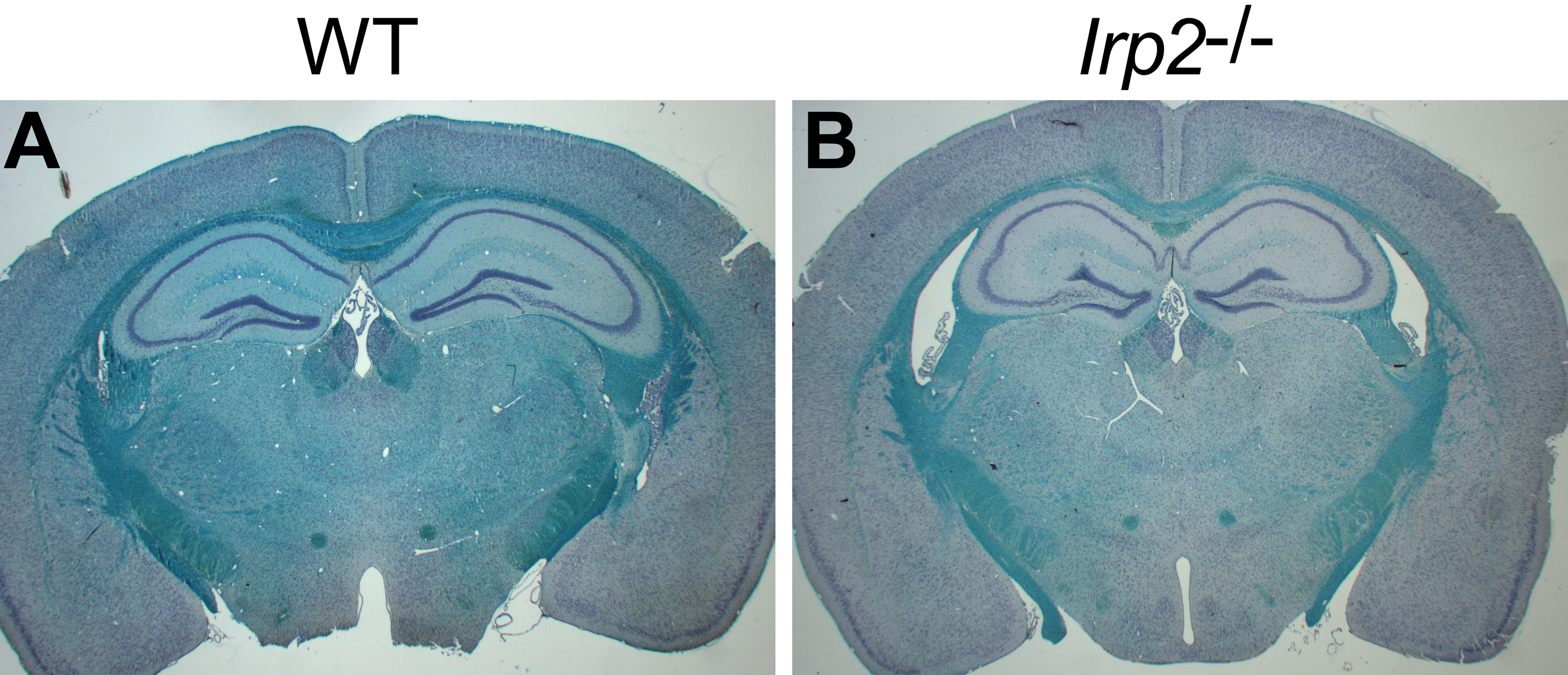

Supplement: Figure S5 — Luxol Fast Blue staining of brains of aged Irp2−/− and WT mice shows no difference in myelinization. Representative images are shown from WT (n = 4) and Irp2−/− (n = 5) mice. Ages of male mice: WT, 65–71 weeks; Irp2−/−, 46–53 weeks. (TIF) [file pone.0098072.s005.tif]

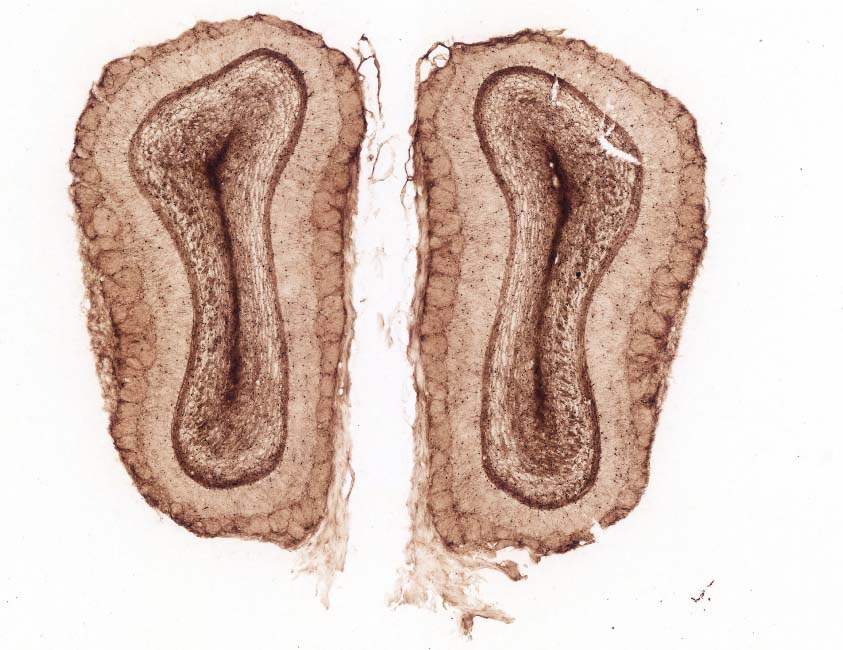

Supplement: File S1 — DAB-enhanced Perls' iron staining of coronal sections of WT (6B-4) brains. Images (10–66) are from rostral to caudal. High resolution files are available from the corresponding author. (ZIP) [file pone.0098072.s012.zip › PerlsIronStain_WT/6B4-10.jpg]

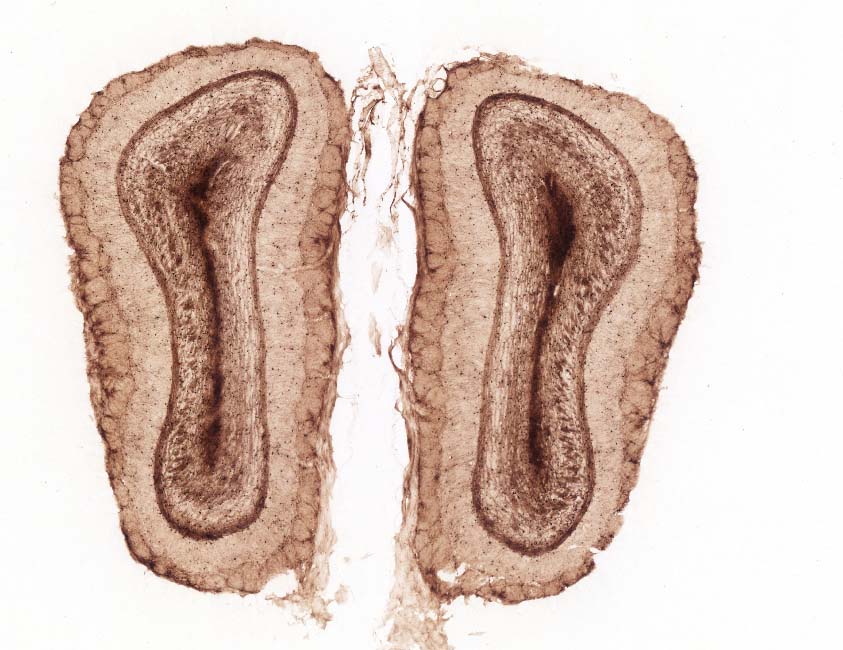

Supplement: File S1 — DAB-enhanced Perls' iron staining of coronal sections of WT (6B-4) brains. Images (10–66) are from rostral to caudal. High resolution files are available from the corresponding author. (ZIP) [file pone.0098072.s012.zip › PerlsIronStain_WT/6B4-11.jpg]

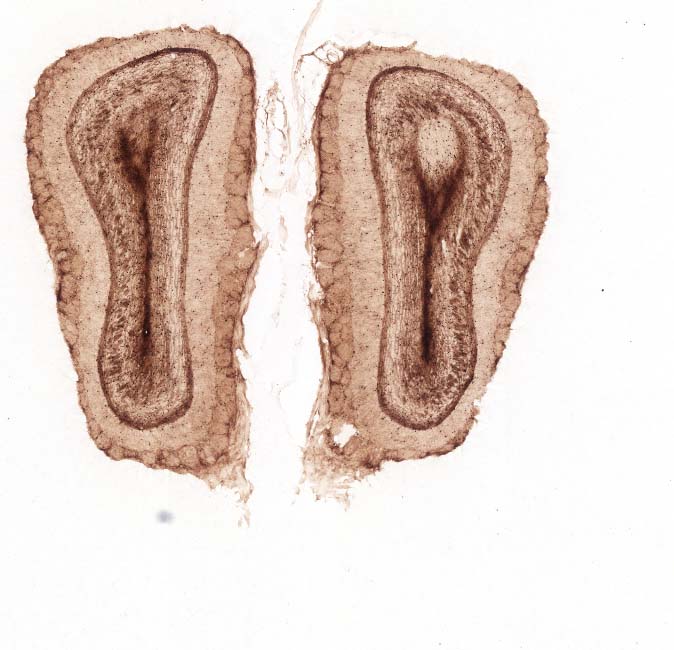

Supplement: File S1 — DAB-enhanced Perls' iron staining of coronal sections of WT (6B-4) brains. Images (10–66) are from rostral to caudal. High resolution files are available from the corresponding author. (ZIP) [file pone.0098072.s012.zip › PerlsIronStain_WT/6B4-12.jpg]

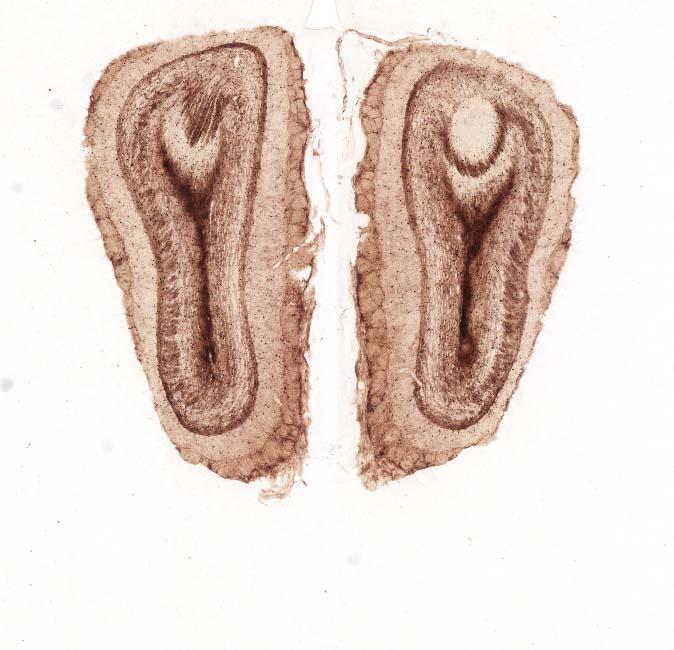

Supplement: File S1 — DAB-enhanced Perls' iron staining of coronal sections of WT (6B-4) brains. Images (10–66) are from rostral to caudal. High resolution files are available from the corresponding author. (ZIP) [file pone.0098072.s012.zip › PerlsIronStain_WT/6B4-13.jpg]

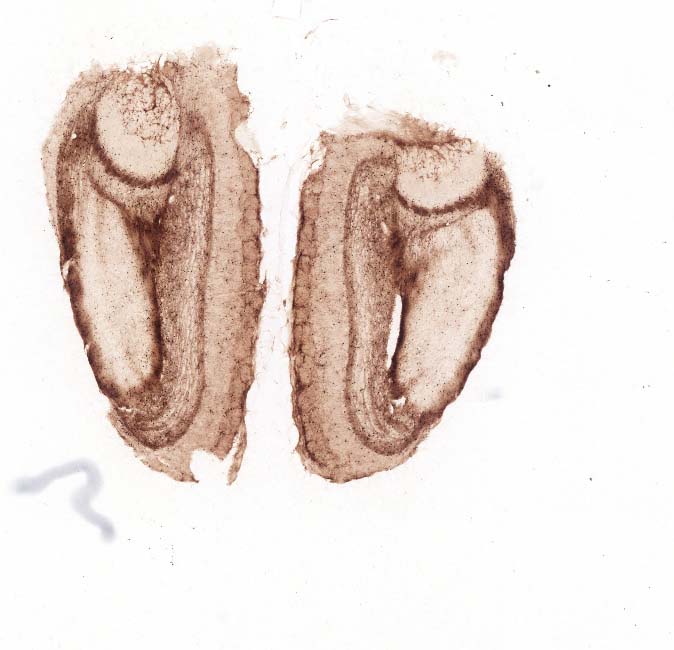

Supplement: File S1 — DAB-enhanced Perls' iron staining of coronal sections of WT (6B-4) brains. Images (10–66) are from rostral to caudal. High resolution files are available from the corresponding author. (ZIP) [file pone.0098072.s012.zip › PerlsIronStain_WT/6B4-14.jpg]

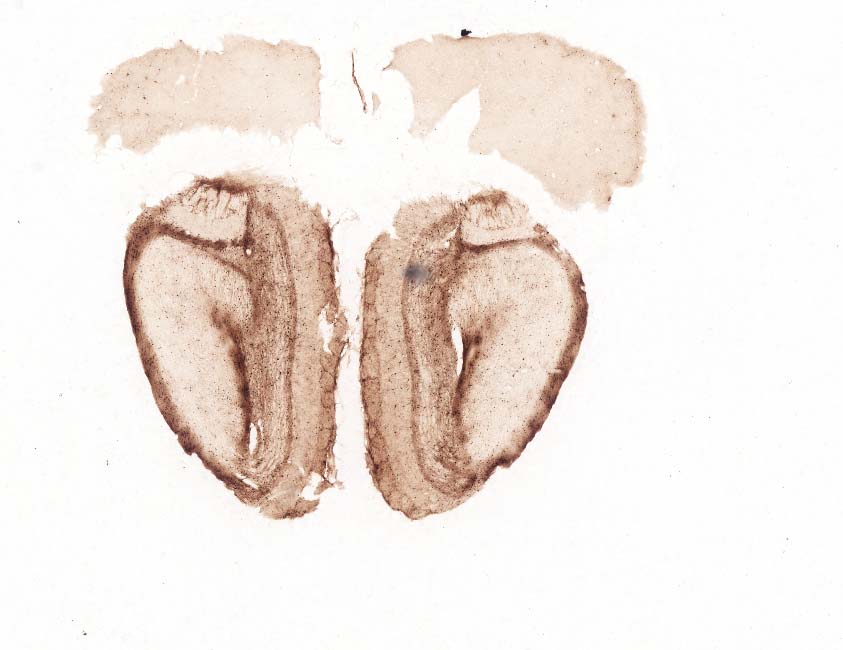

Supplement: File S1 — DAB-enhanced Perls' iron staining of coronal sections of WT (6B-4) brains. Images (10–66) are from rostral to caudal. High resolution files are available from the corresponding author. (ZIP) [file pone.0098072.s012.zip › PerlsIronStain_WT/6B4-15.jpg]

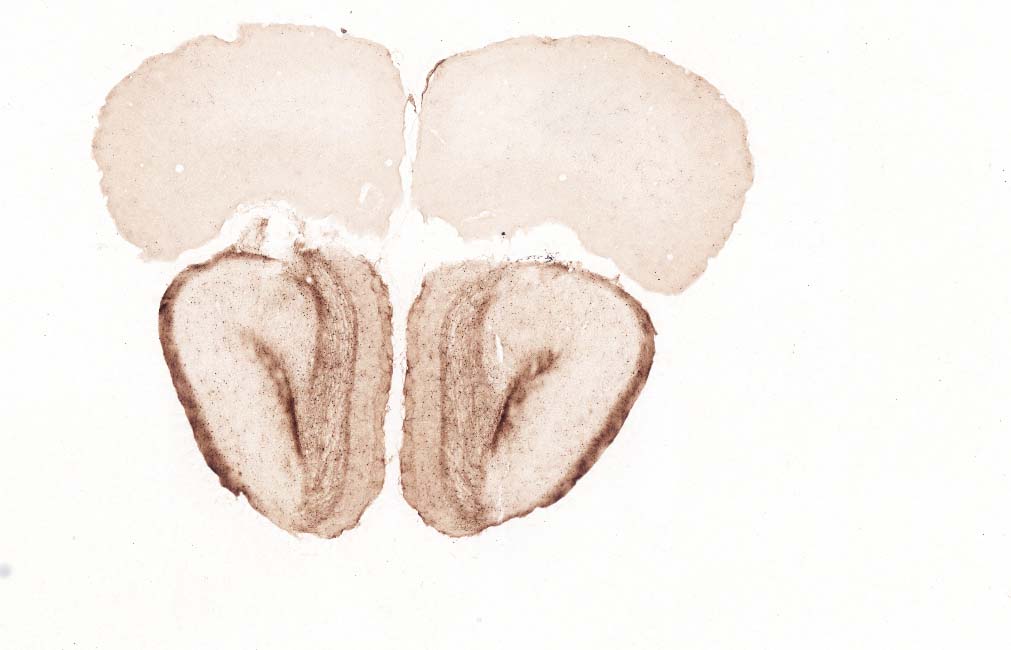

Supplement: File S1 — DAB-enhanced Perls' iron staining of coronal sections of WT (6B-4) brains. Images (10–66) are from rostral to caudal. High resolution files are available from the corresponding author. (ZIP) [file pone.0098072.s012.zip › PerlsIronStain_WT/6B4-16.jpg]

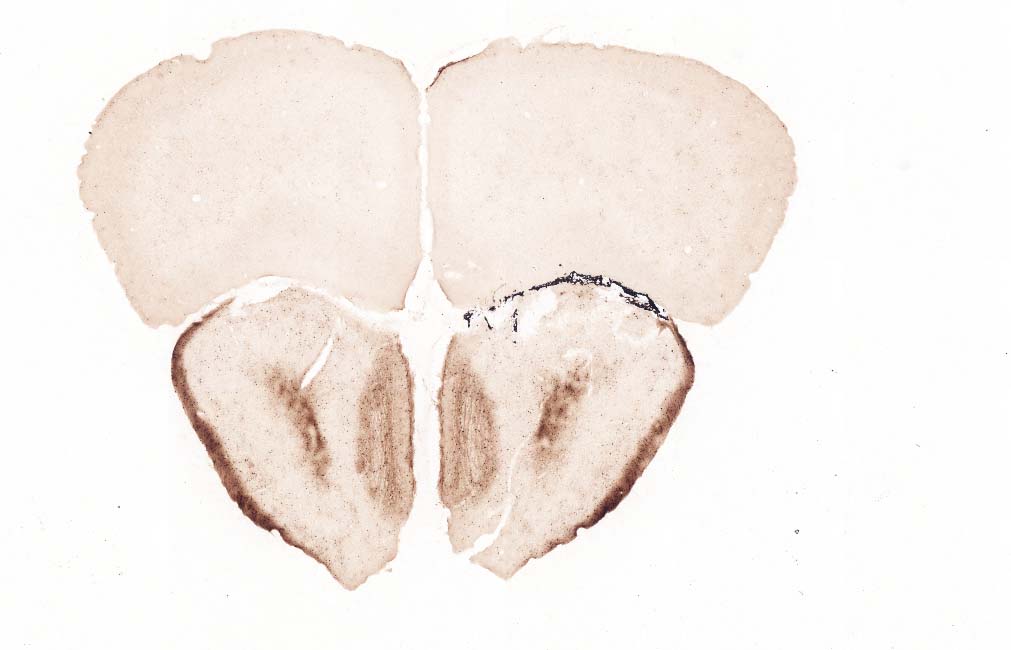

Supplement: File S1 — DAB-enhanced Perls' iron staining of coronal sections of WT (6B-4) brains. Images (10–66) are from rostral to caudal. High resolution files are available from the corresponding author. (ZIP) [file pone.0098072.s012.zip › PerlsIronStain_WT/6B4-17.jpg]

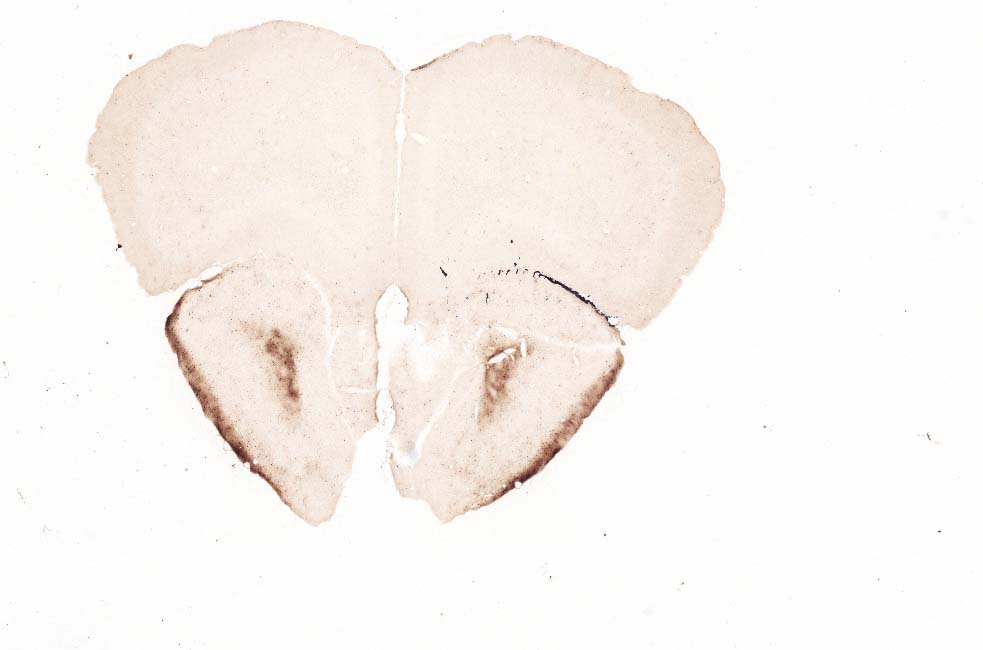

Supplement: File S1 — DAB-enhanced Perls' iron staining of coronal sections of WT (6B-4) brains. Images (10–66) are from rostral to caudal. High resolution files are available from the corresponding author. (ZIP) [file pone.0098072.s012.zip › PerlsIronStain_WT/6B4-18.jpg]

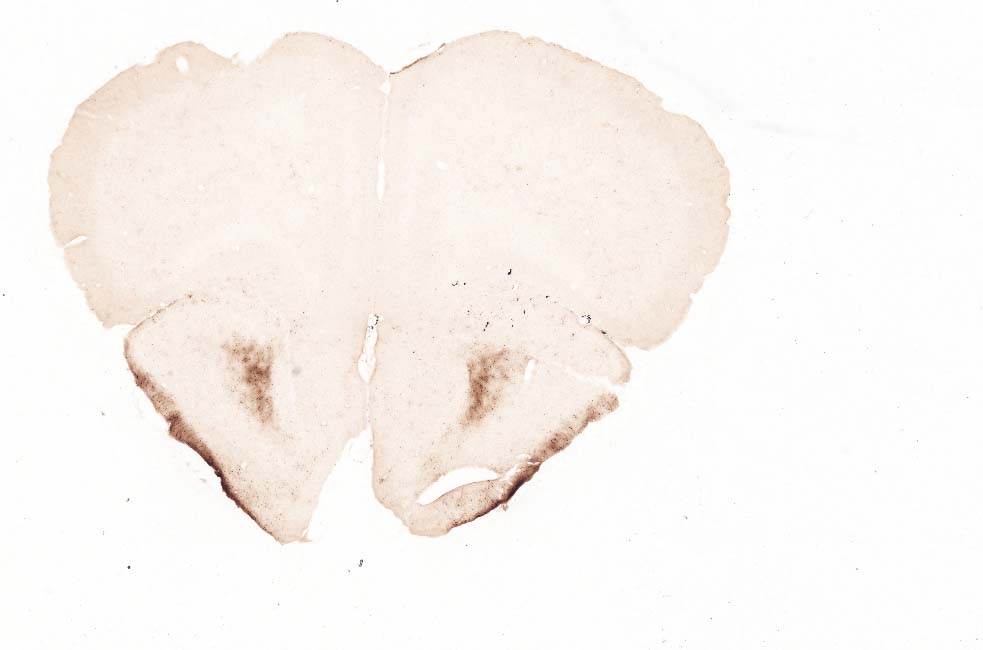

Supplement: File S1 — DAB-enhanced Perls' iron staining of coronal sections of WT (6B-4) brains. Images (10–66) are from rostral to caudal. High resolution files are available from the corresponding author. (ZIP) [file pone.0098072.s012.zip › PerlsIronStain_WT/6B4-19.jpg]

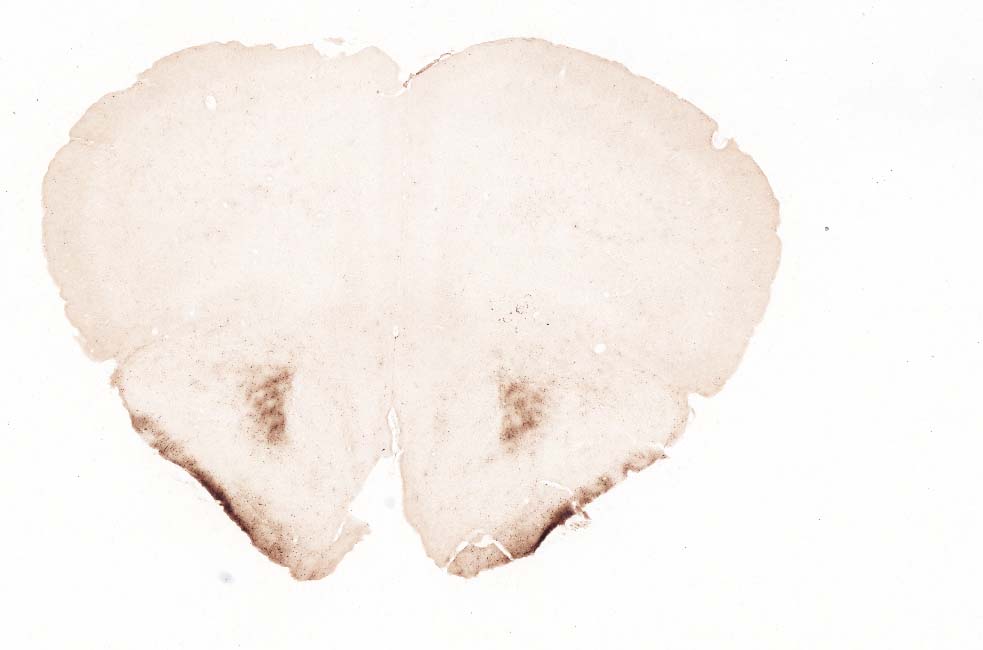

Supplement: File S1 — DAB-enhanced Perls' iron staining of coronal sections of WT (6B-4) brains. Images (10–66) are from rostral to caudal. High resolution files are available from the corresponding author. (ZIP) [file pone.0098072.s012.zip › PerlsIronStain_WT/6B4-20.jpg]

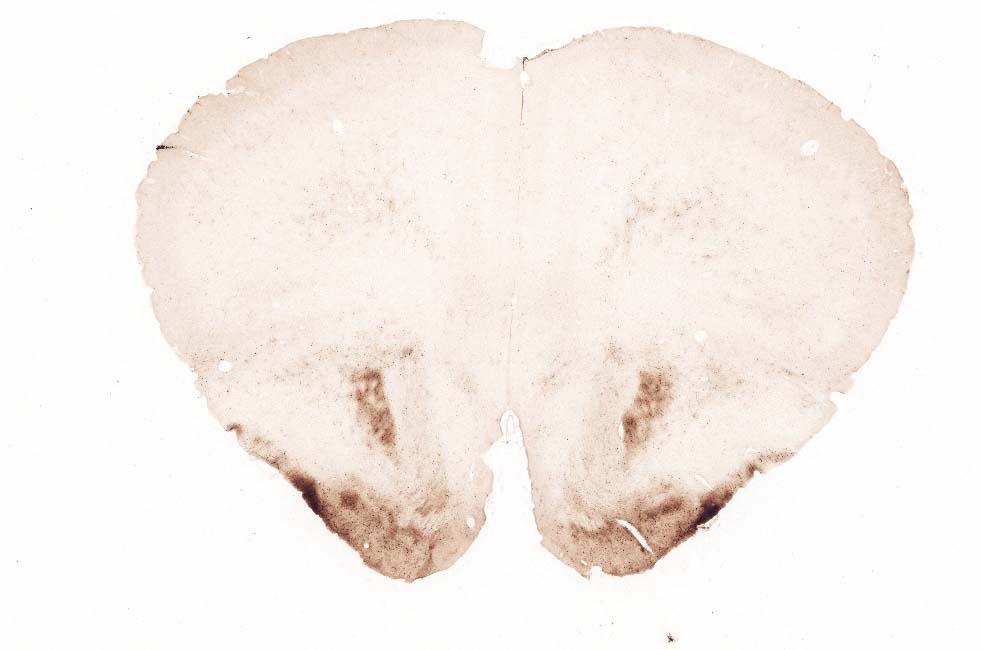

Supplement: File S1 — DAB-enhanced Perls' iron staining of coronal sections of WT (6B-4) brains. Images (10–66) are from rostral to caudal. High resolution files are available from the corresponding author. (ZIP) [file pone.0098072.s012.zip › PerlsIronStain_WT/6B4-21.jpg]

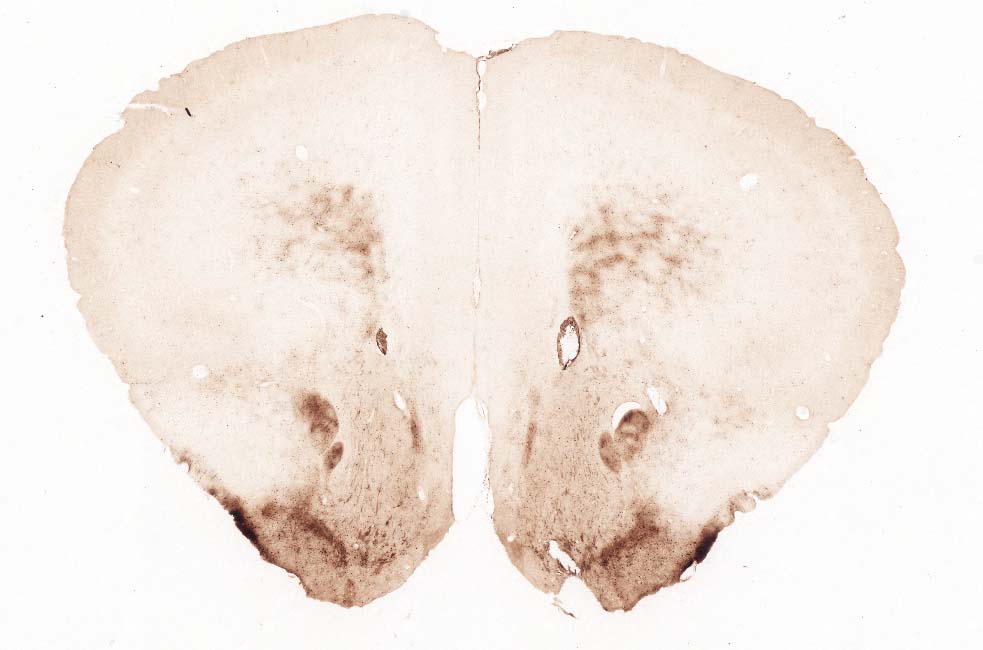

Supplement: File S1 — DAB-enhanced Perls' iron staining of coronal sections of WT (6B-4) brains. Images (10–66) are from rostral to caudal. High resolution files are available from the corresponding author. (ZIP) [file pone.0098072.s012.zip › PerlsIronStain_WT/6B4-22.jpg]

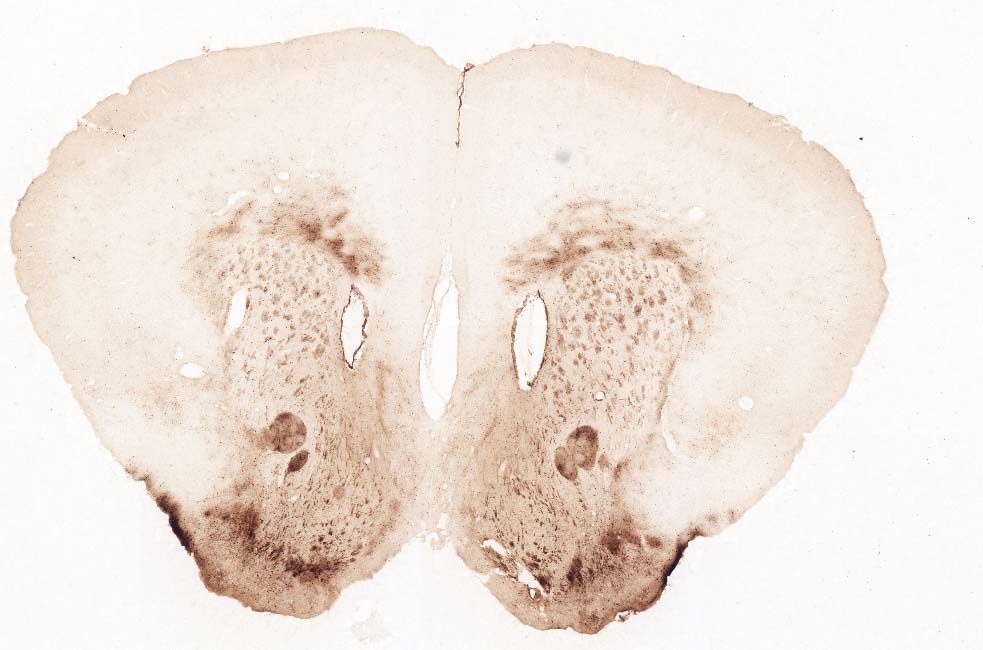

Supplement: File S1 — DAB-enhanced Perls' iron staining of coronal sections of WT (6B-4) brains. Images (10–66) are from rostral to caudal. High resolution files are available from the corresponding author. (ZIP) [file pone.0098072.s012.zip › PerlsIronStain_WT/6B4-23.jpg]

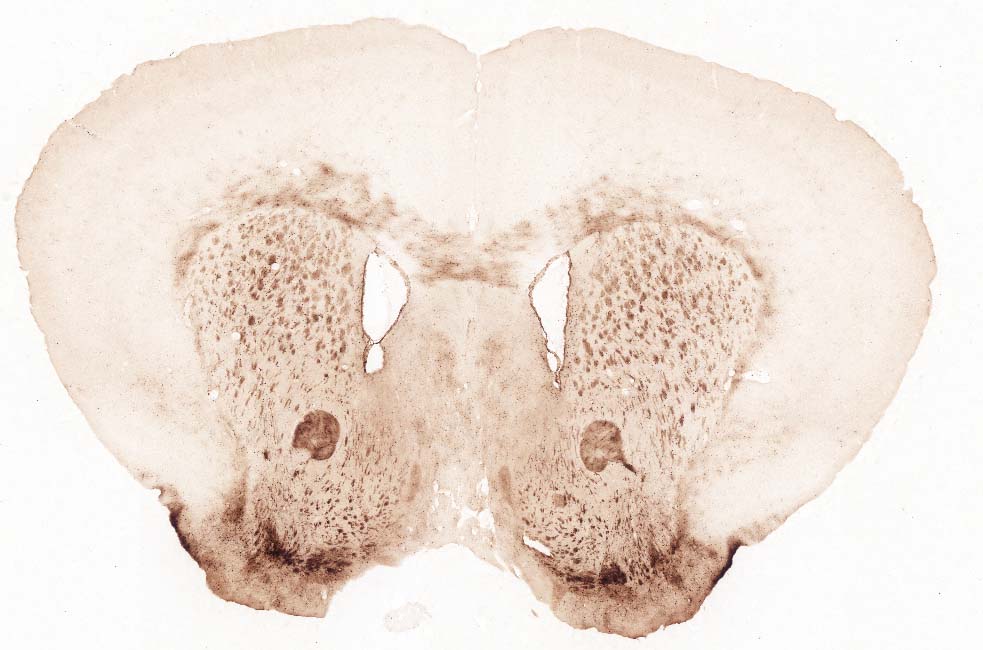

Supplement: File S1 — DAB-enhanced Perls' iron staining of coronal sections of WT (6B-4) brains. Images (10–66) are from rostral to caudal. High resolution files are available from the corresponding author. (ZIP) [file pone.0098072.s012.zip › PerlsIronStain_WT/6B4-24.jpg]

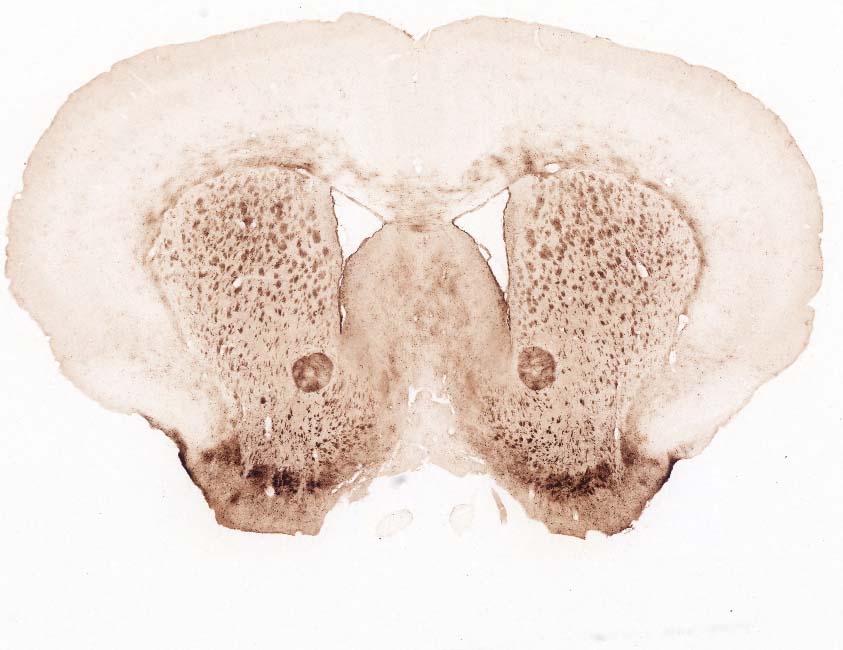

Supplement: File S1 — DAB-enhanced Perls' iron staining of coronal sections of WT (6B-4) brains. Images (10–66) are from rostral to caudal. High resolution files are available from the corresponding author. (ZIP) [file pone.0098072.s012.zip › PerlsIronStain_WT/6B4-25.jpg]

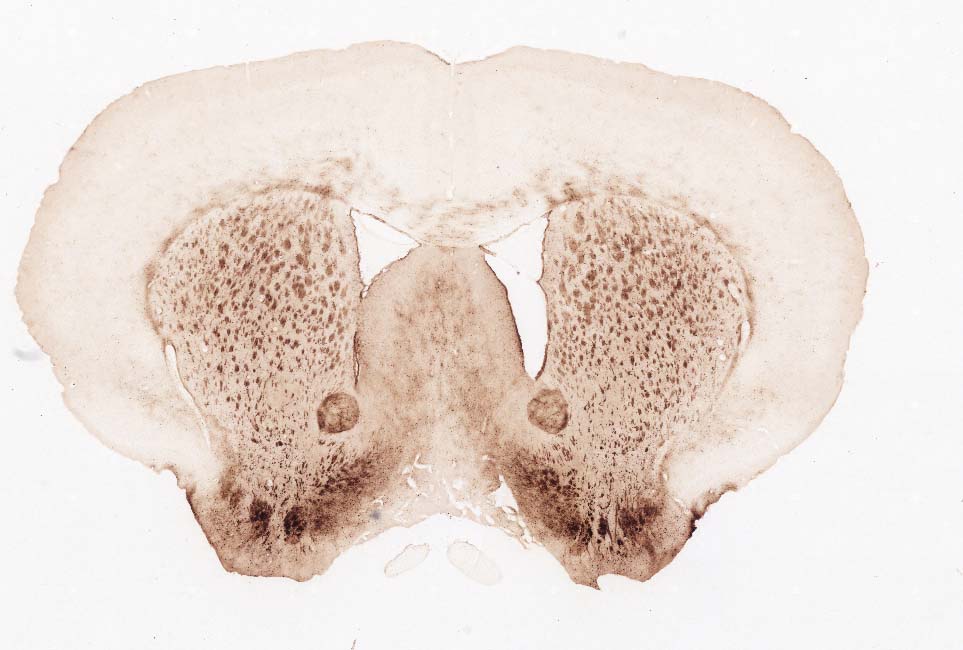

Supplement: File S1 — DAB-enhanced Perls' iron staining of coronal sections of WT (6B-4) brains. Images (10–66) are from rostral to caudal. High resolution files are available from the corresponding author. (ZIP) [file pone.0098072.s012.zip › PerlsIronStain_WT/6B4-26.jpg]

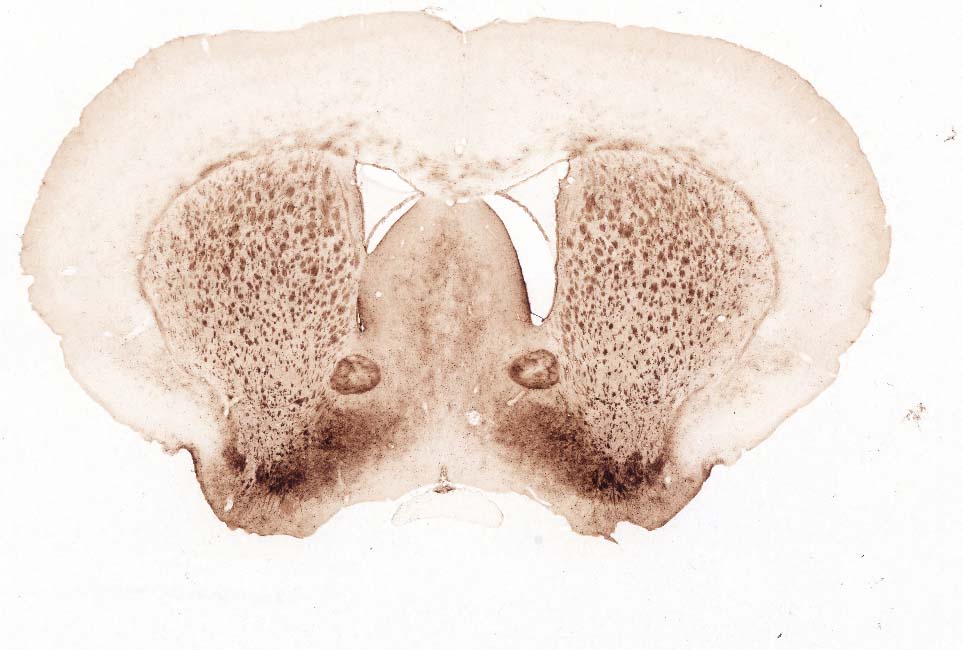

Supplement: File S1 — DAB-enhanced Perls' iron staining of coronal sections of WT (6B-4) brains. Images (10–66) are from rostral to caudal. High resolution files are available from the corresponding author. (ZIP) [file pone.0098072.s012.zip › PerlsIronStain_WT/6B4-27.jpg]

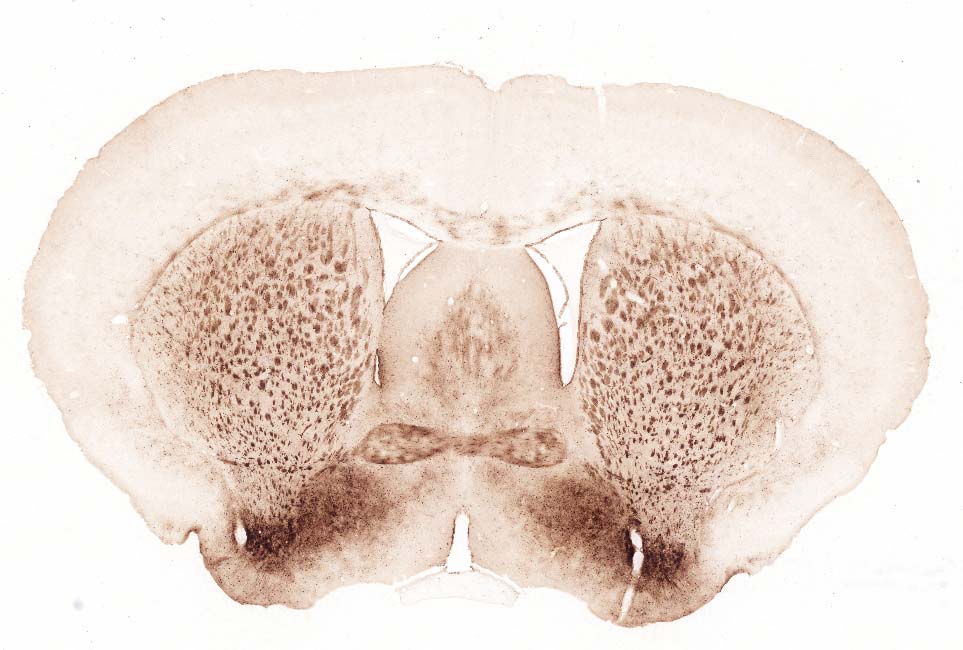

Supplement: File S1 — DAB-enhanced Perls' iron staining of coronal sections of WT (6B-4) brains. Images (10–66) are from rostral to caudal. High resolution files are available from the corresponding author. (ZIP) [file pone.0098072.s012.zip › PerlsIronStain_WT/6B4-28.jpg]

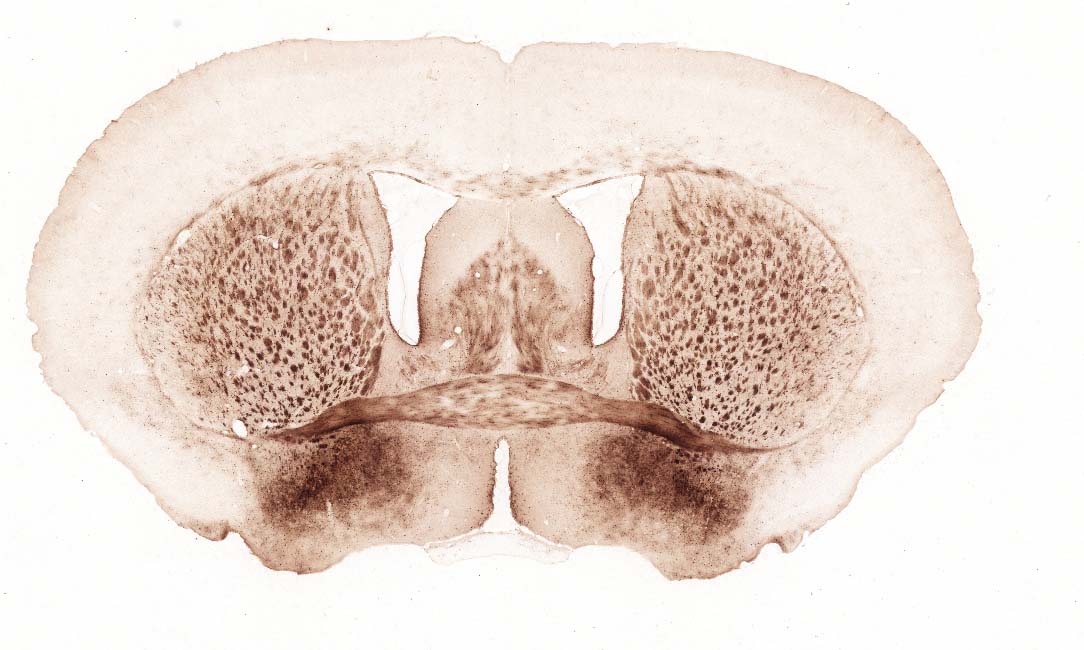

Supplement: File S1 — DAB-enhanced Perls' iron staining of coronal sections of WT (6B-4) brains. Images (10–66) are from rostral to caudal. High resolution files are available from the corresponding author. (ZIP) [file pone.0098072.s012.zip › PerlsIronStain_WT/6B4-29.jpg]

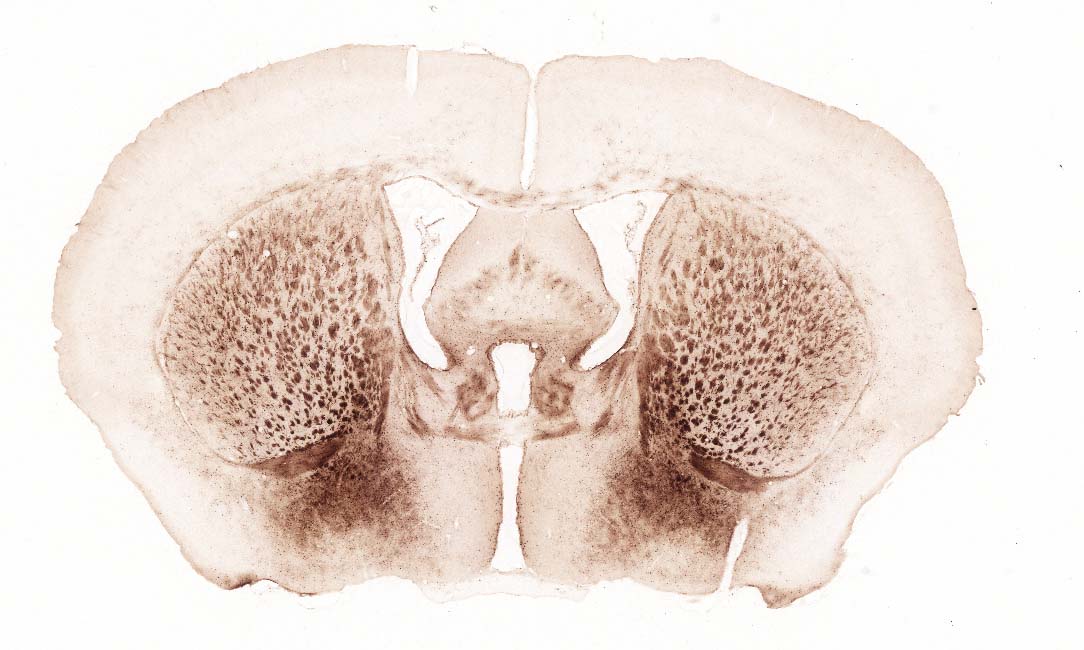

Supplement: File S1 — DAB-enhanced Perls' iron staining of coronal sections of WT (6B-4) brains. Images (10–66) are from rostral to caudal. High resolution files are available from the corresponding author. (ZIP) [file pone.0098072.s012.zip › PerlsIronStain_WT/6B4-30.jpg]

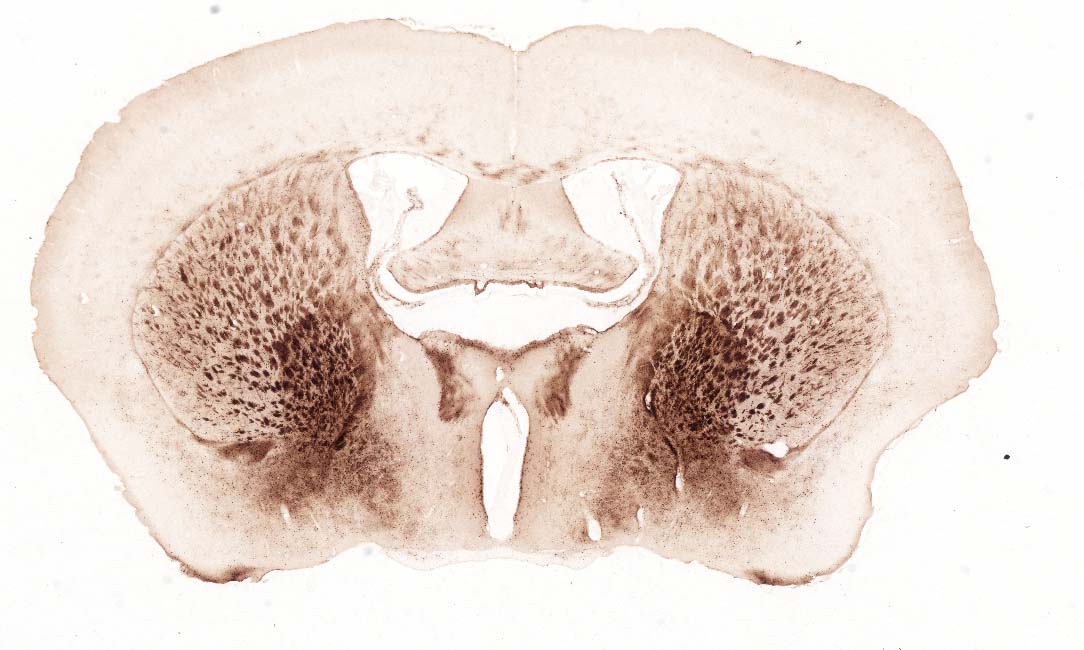

Supplement: File S1 — DAB-enhanced Perls' iron staining of coronal sections of WT (6B-4) brains. Images (10–66) are from rostral to caudal. High resolution files are available from the corresponding author. (ZIP) [file pone.0098072.s012.zip › PerlsIronStain_WT/6B4-31.jpg]

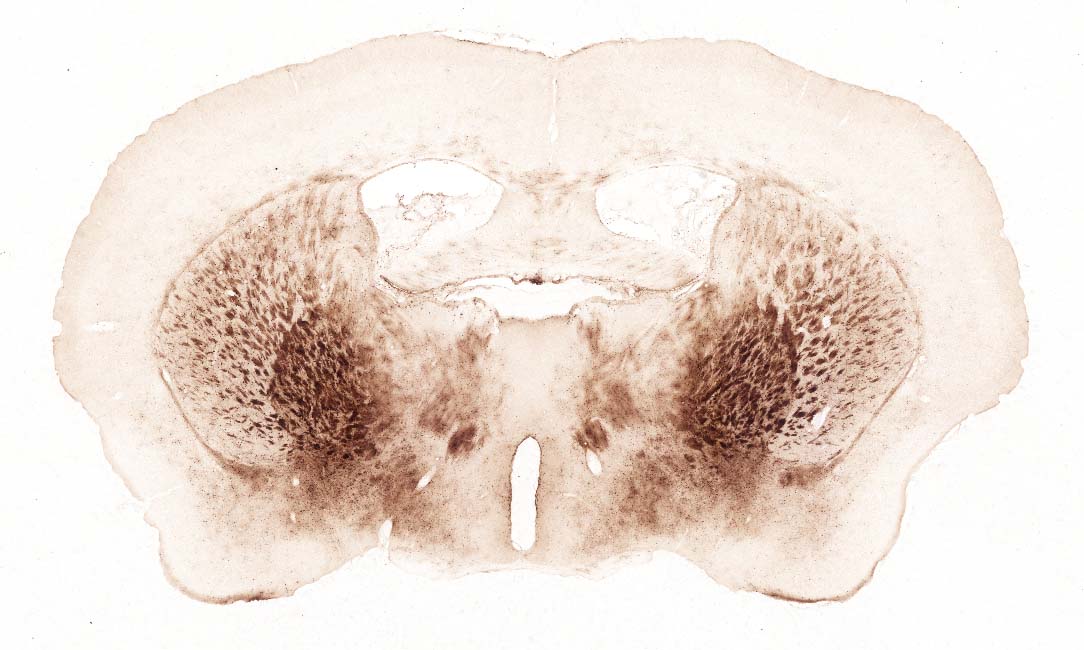

Supplement: File S1 — DAB-enhanced Perls' iron staining of coronal sections of WT (6B-4) brains. Images (10–66) are from rostral to caudal. High resolution files are available from the corresponding author. (ZIP) [file pone.0098072.s012.zip › PerlsIronStain_WT/6B4-32.jpg]

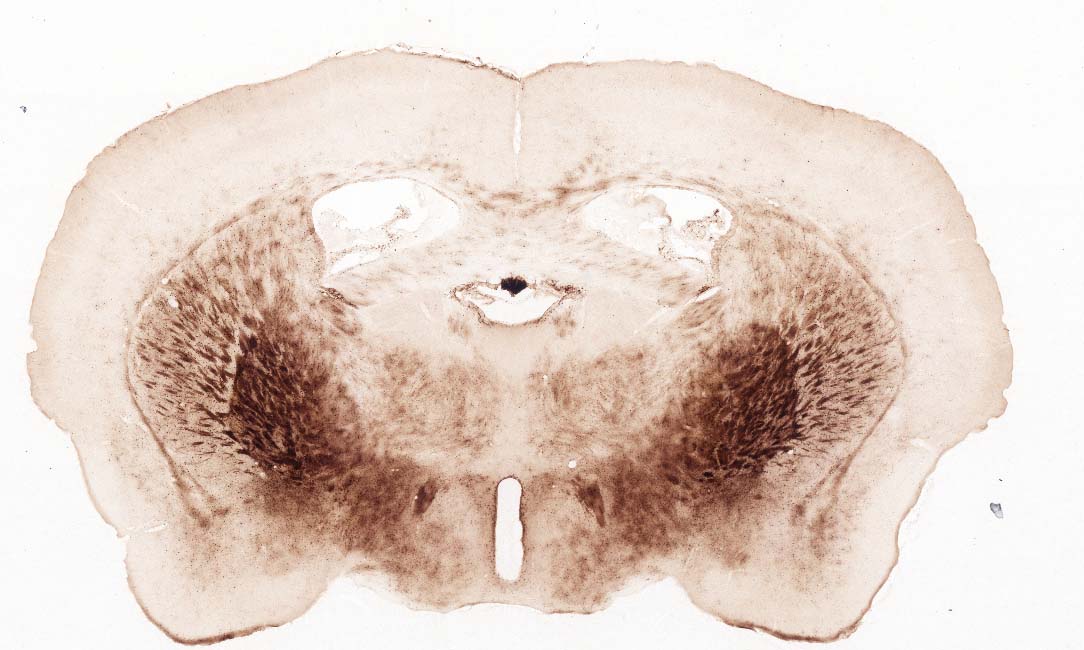

Supplement: File S1 — DAB-enhanced Perls' iron staining of coronal sections of WT (6B-4) brains. Images (10–66) are from rostral to caudal. High resolution files are available from the corresponding author. (ZIP) [file pone.0098072.s012.zip › PerlsIronStain_WT/6B4-33.jpg]

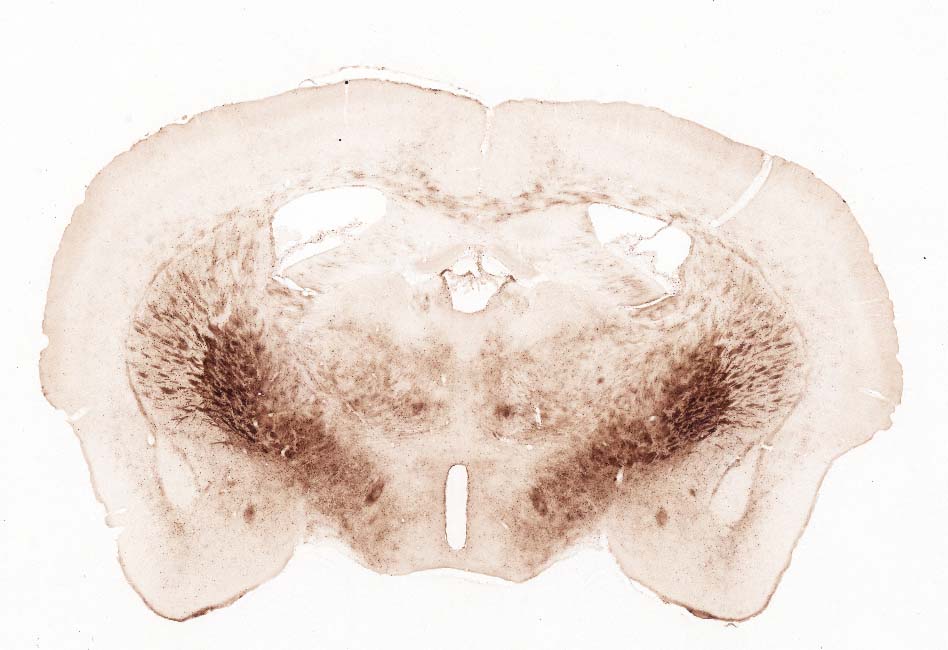

Supplement: File S1 — DAB-enhanced Perls' iron staining of coronal sections of WT (6B-4) brains. Images (10–66) are from rostral to caudal. High resolution files are available from the corresponding author. (ZIP) [file pone.0098072.s012.zip › PerlsIronStain_WT/6B4-34.jpg]

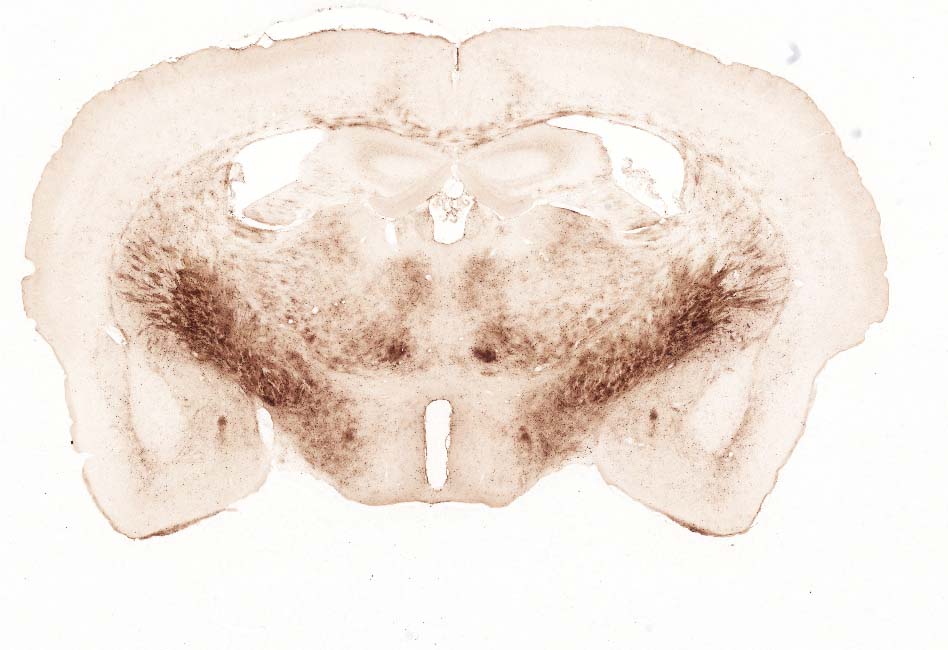

Supplement: File S1 — DAB-enhanced Perls' iron staining of coronal sections of WT (6B-4) brains. Images (10–66) are from rostral to caudal. High resolution files are available from the corresponding author. (ZIP) [file pone.0098072.s012.zip › PerlsIronStain_WT/6B4-35.jpg]

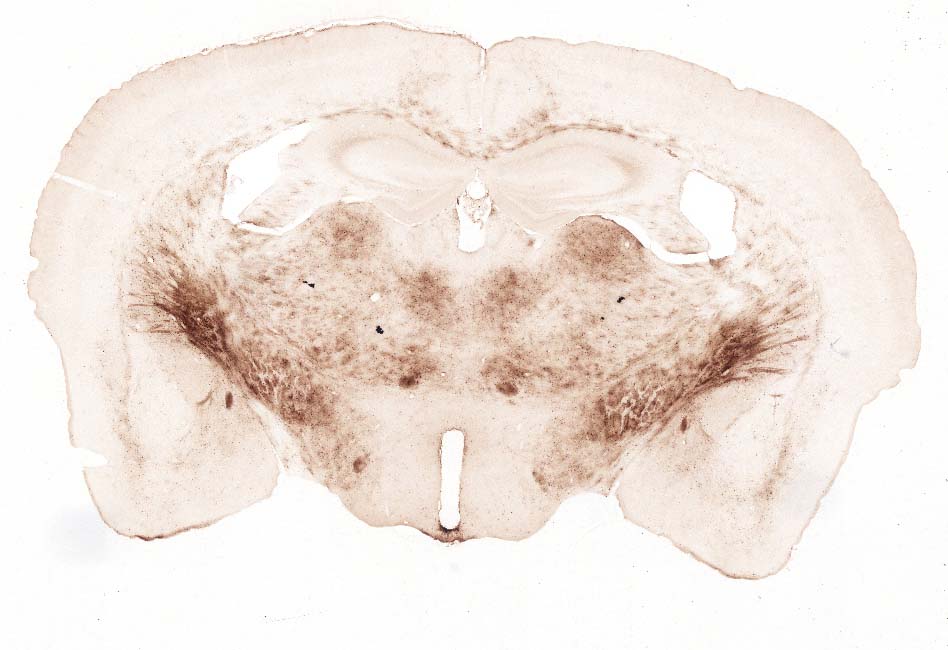

Supplement: File S1 — DAB-enhanced Perls' iron staining of coronal sections of WT (6B-4) brains. Images (10–66) are from rostral to caudal. High resolution files are available from the corresponding author. (ZIP) [file pone.0098072.s012.zip › PerlsIronStain_WT/6B4-36.jpg]

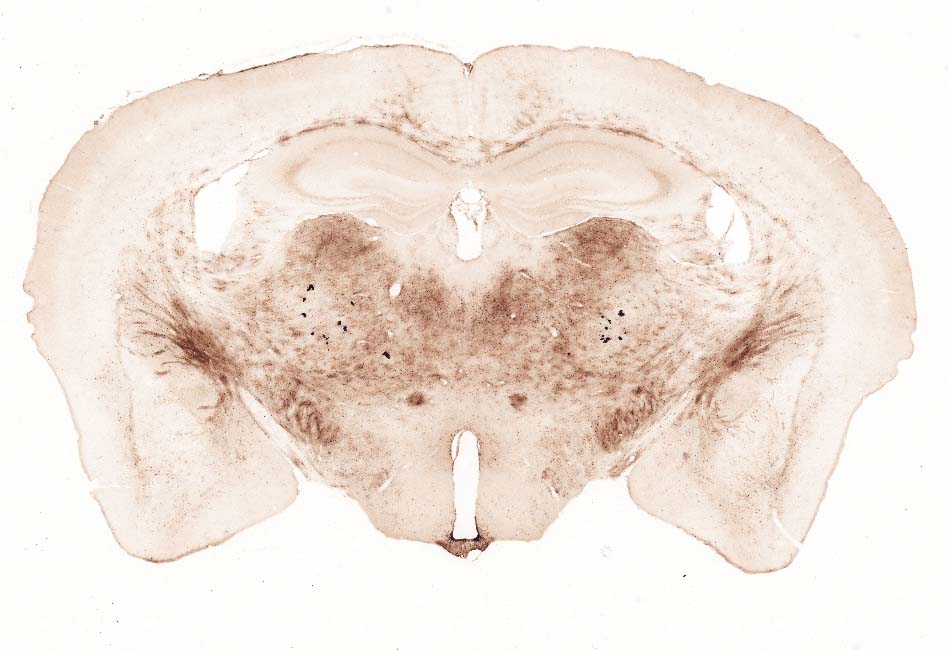

Supplement: File S1 — DAB-enhanced Perls' iron staining of coronal sections of WT (6B-4) brains. Images (10–66) are from rostral to caudal. High resolution files are available from the corresponding author. (ZIP) [file pone.0098072.s012.zip › PerlsIronStain_WT/6B4-37.jpg]

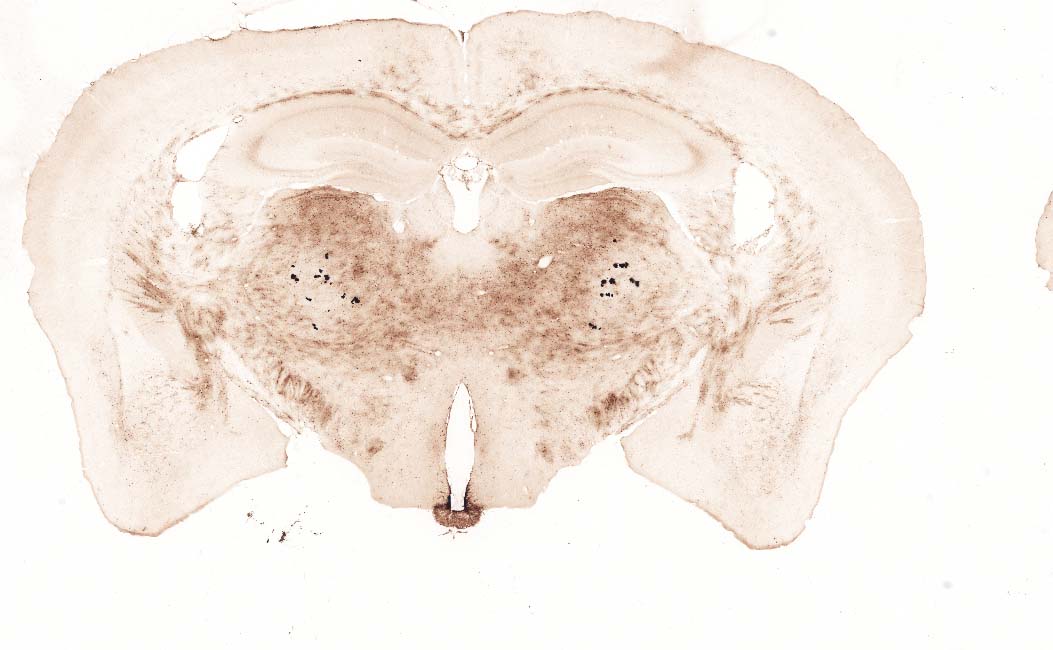

Supplement: File S1 — DAB-enhanced Perls' iron staining of coronal sections of WT (6B-4) brains. Images (10–66) are from rostral to caudal. High resolution files are available from the corresponding author. (ZIP) [file pone.0098072.s012.zip › PerlsIronStain_WT/6B4-38.jpg]

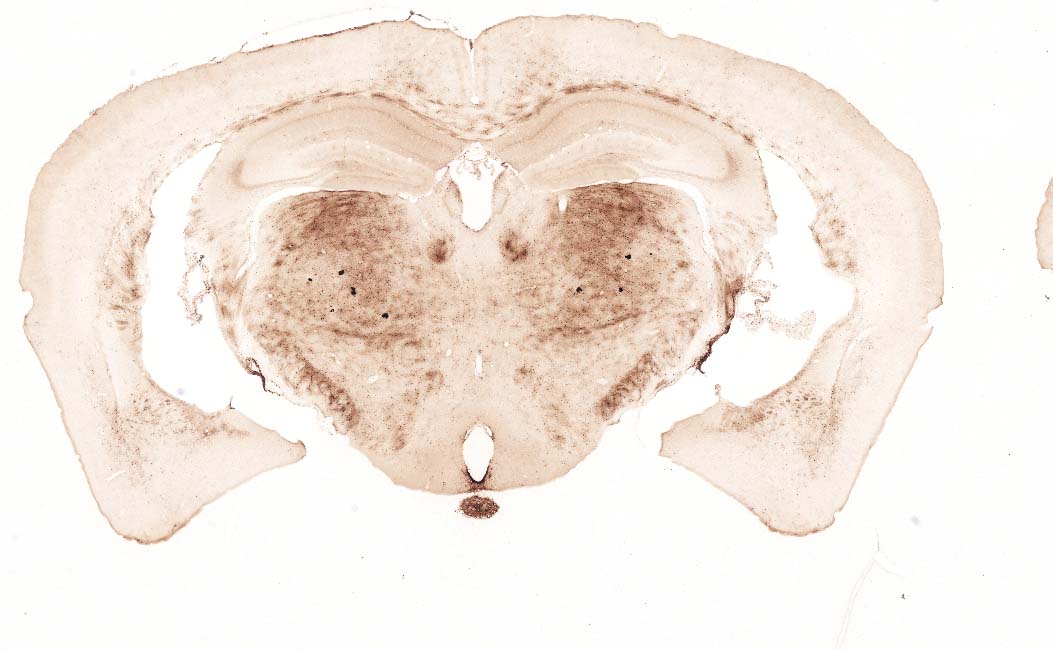

Supplement: File S1 — DAB-enhanced Perls' iron staining of coronal sections of WT (6B-4) brains. Images (10–66) are from rostral to caudal. High resolution files are available from the corresponding author. (ZIP) [file pone.0098072.s012.zip › PerlsIronStain_WT/6B4-39.jpg]

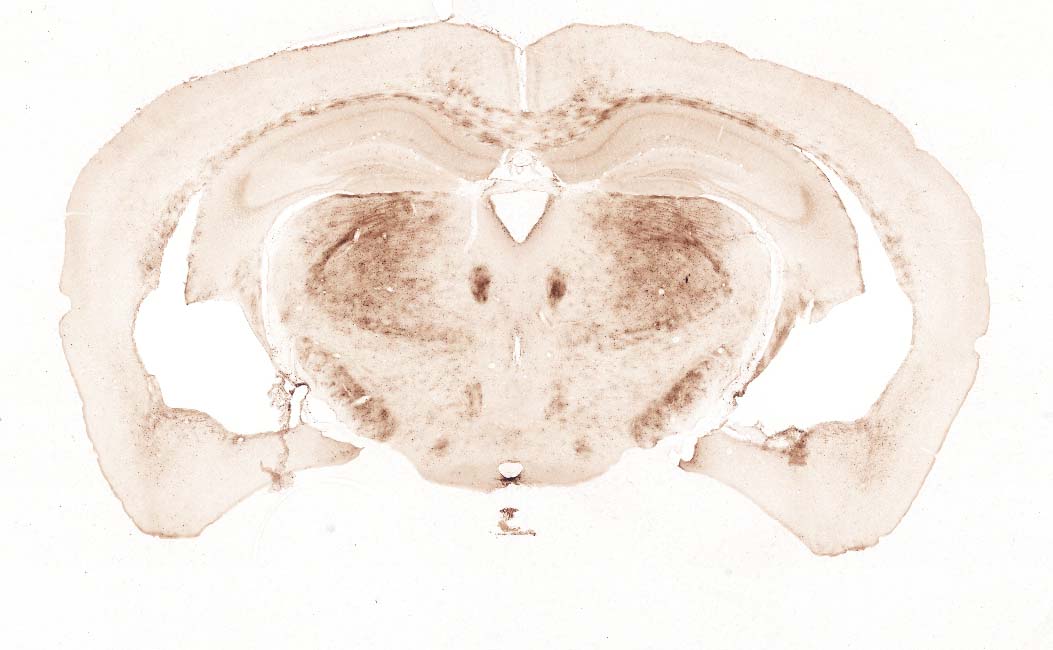

Supplement: File S1 — DAB-enhanced Perls' iron staining of coronal sections of WT (6B-4) brains. Images (10–66) are from rostral to caudal. High resolution files are available from the corresponding author. (ZIP) [file pone.0098072.s012.zip › PerlsIronStain_WT/6B4-40.jpg]

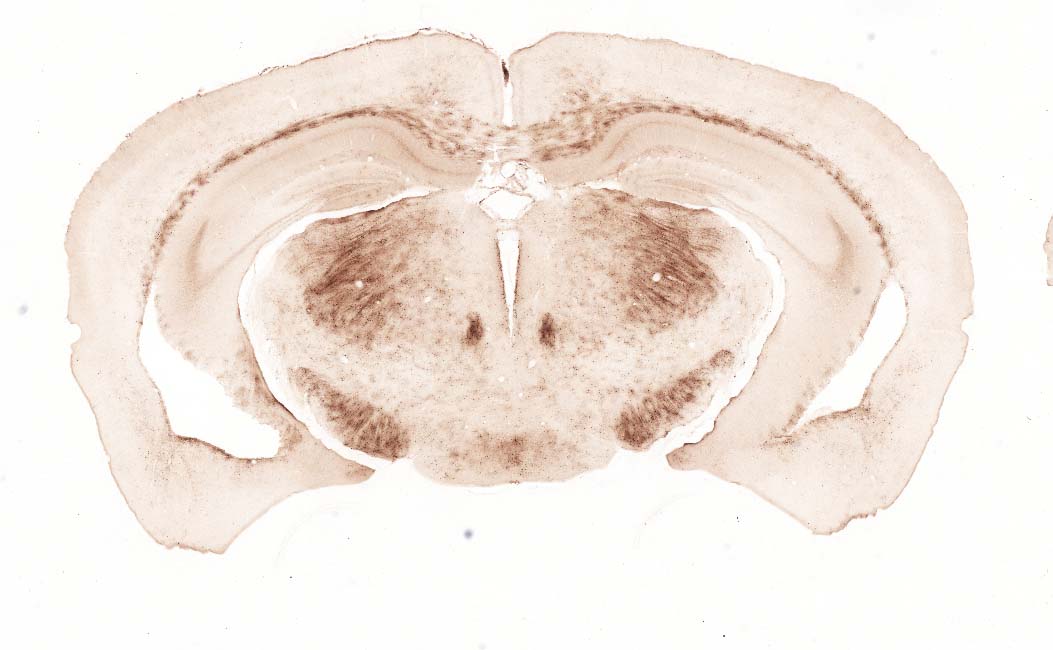

Supplement: File S1 — DAB-enhanced Perls' iron staining of coronal sections of WT (6B-4) brains. Images (10–66) are from rostral to caudal. High resolution files are available from the corresponding author. (ZIP) [file pone.0098072.s012.zip › PerlsIronStain_WT/6B4-41.jpg]

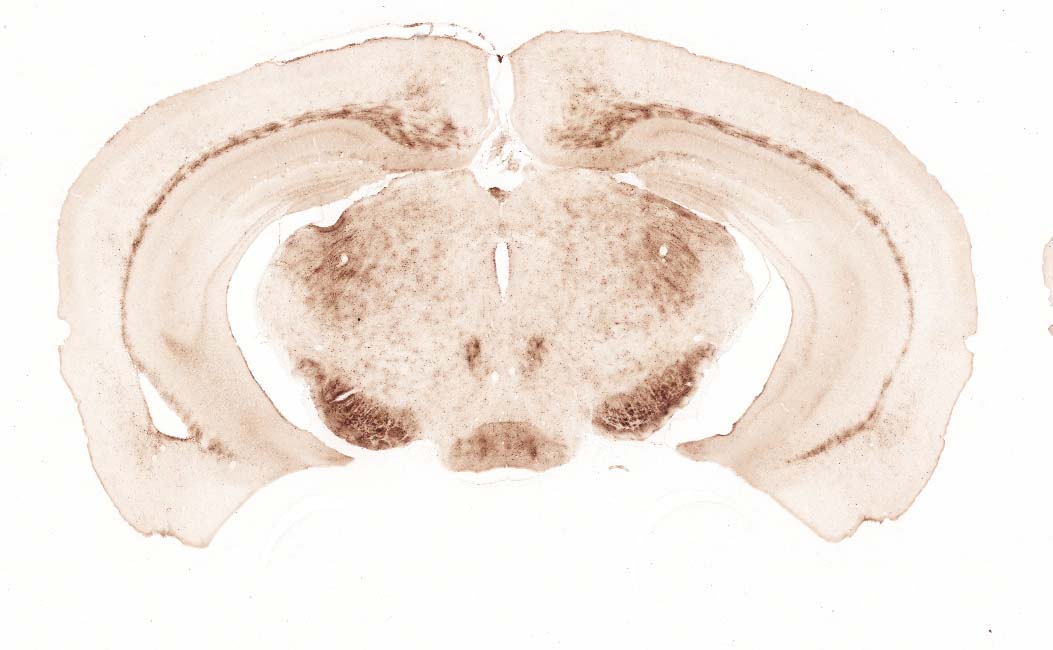

Supplement: File S1 — DAB-enhanced Perls' iron staining of coronal sections of WT (6B-4) brains. Images (10–66) are from rostral to caudal. High resolution files are available from the corresponding author. (ZIP) [file pone.0098072.s012.zip › PerlsIronStain_WT/6B4-42.jpg]

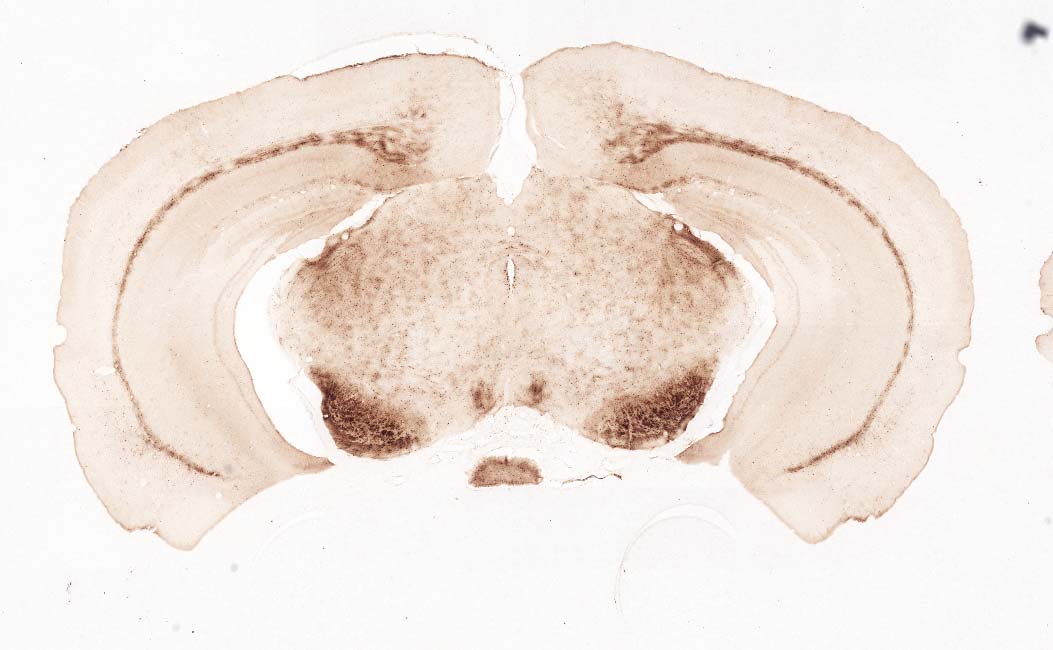

Supplement: File S1 — DAB-enhanced Perls' iron staining of coronal sections of WT (6B-4) brains. Images (10–66) are from rostral to caudal. High resolution files are available from the corresponding author. (ZIP) [file pone.0098072.s012.zip › PerlsIronStain_WT/6B4-43.jpg]

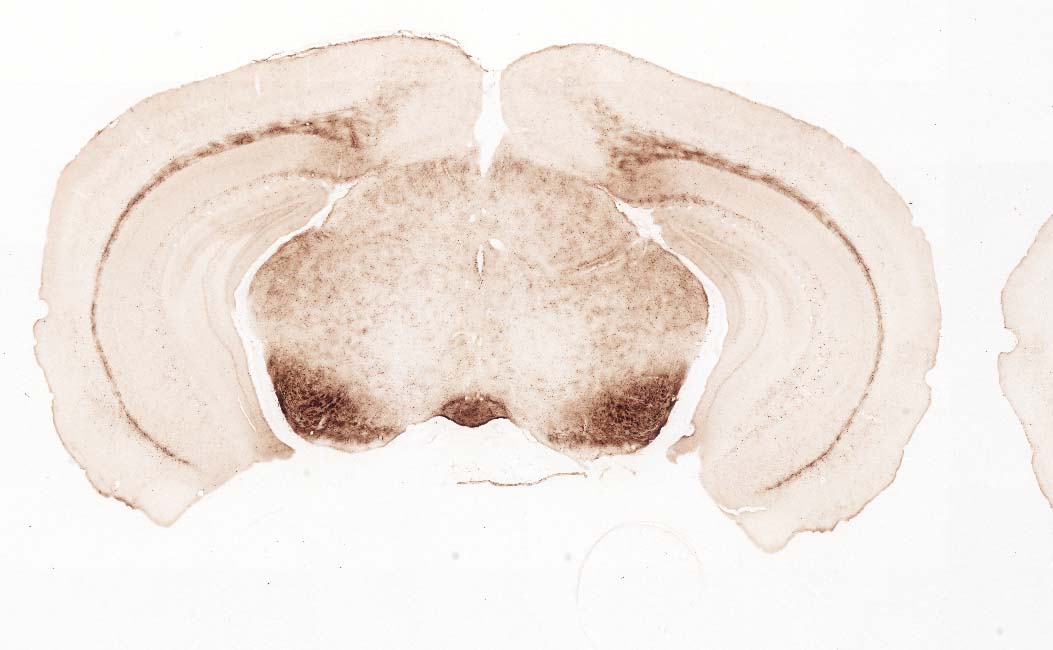

Supplement: File S1 — DAB-enhanced Perls' iron staining of coronal sections of WT (6B-4) brains. Images (10–66) are from rostral to caudal. High resolution files are available from the corresponding author. (ZIP) [file pone.0098072.s012.zip › PerlsIronStain_WT/6B4-44.jpg]

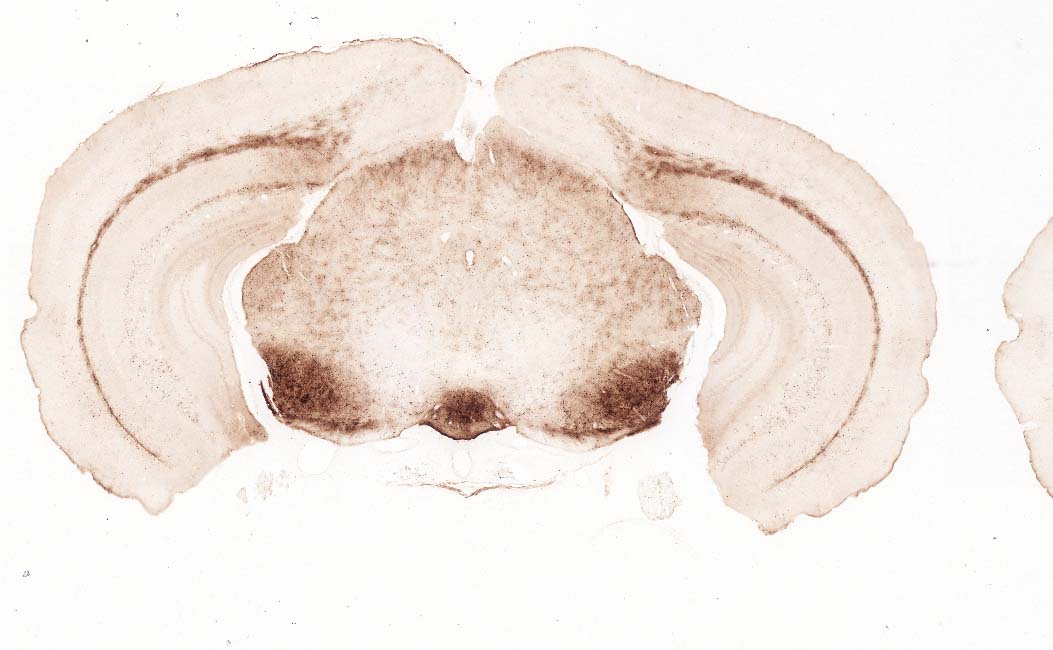

Supplement: File S1 — DAB-enhanced Perls' iron staining of coronal sections of WT (6B-4) brains. Images (10–66) are from rostral to caudal. High resolution files are available from the corresponding author. (ZIP) [file pone.0098072.s012.zip › PerlsIronStain_WT/6B4-45.jpg]

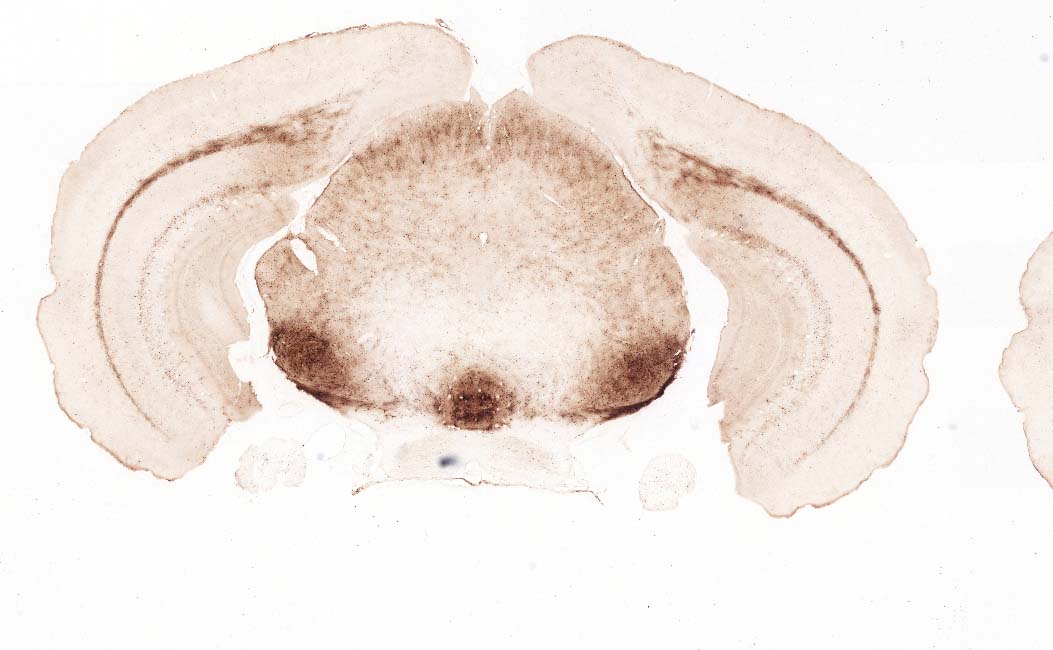

Supplement: File S1 — DAB-enhanced Perls' iron staining of coronal sections of WT (6B-4) brains. Images (10–66) are from rostral to caudal. High resolution files are available from the corresponding author. (ZIP) [file pone.0098072.s012.zip › PerlsIronStain_WT/6B4-46.jpg]

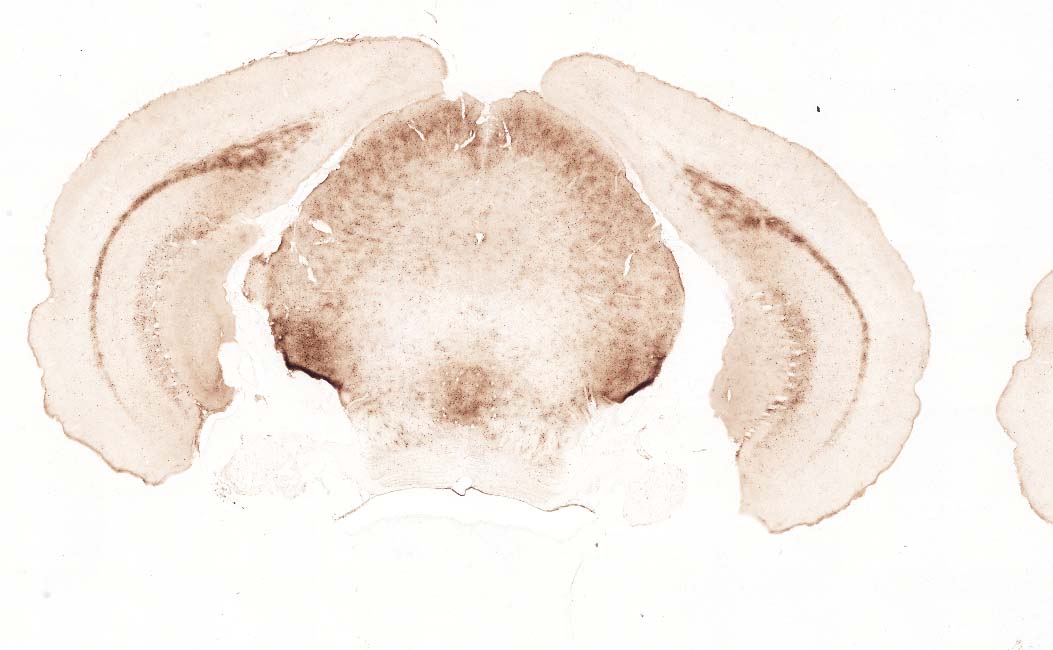

Supplement: File S1 — DAB-enhanced Perls' iron staining of coronal sections of WT (6B-4) brains. Images (10–66) are from rostral to caudal. High resolution files are available from the corresponding author. (ZIP) [file pone.0098072.s012.zip › PerlsIronStain_WT/6B4-47.jpg]

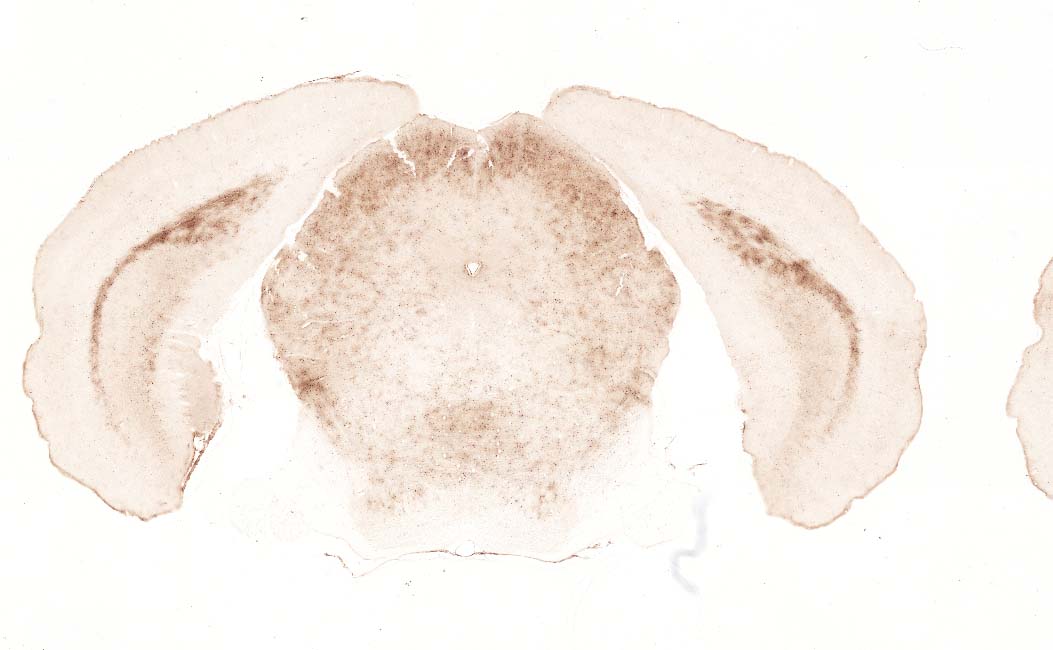

Supplement: File S1 — DAB-enhanced Perls' iron staining of coronal sections of WT (6B-4) brains. Images (10–66) are from rostral to caudal. High resolution files are available from the corresponding author. (ZIP) [file pone.0098072.s012.zip › PerlsIronStain_WT/6B4-48.jpg]

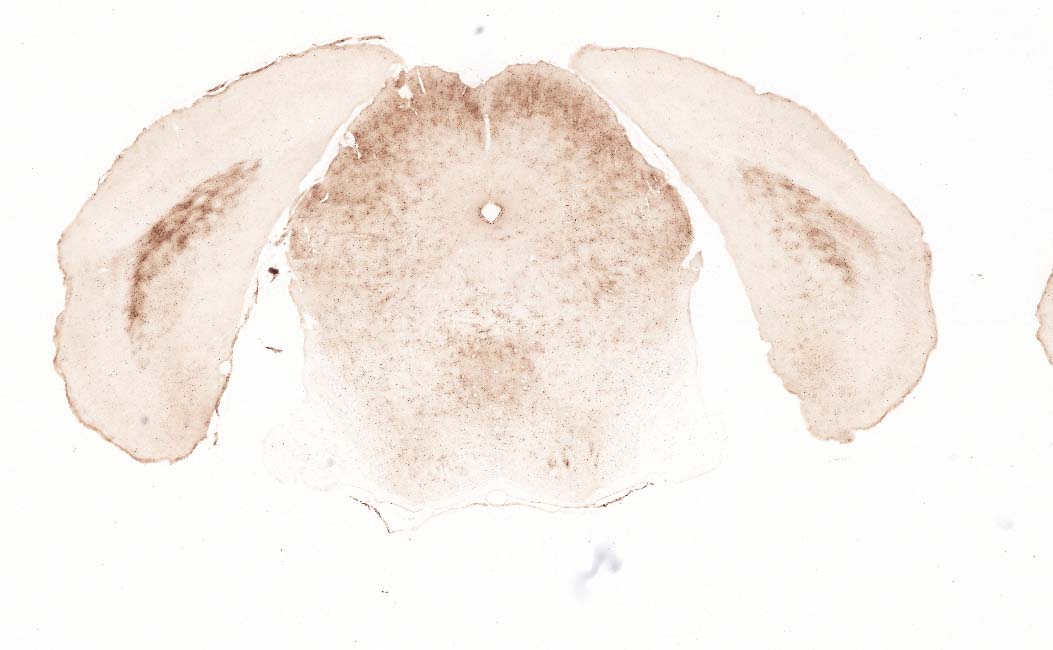

Supplement: File S1 — DAB-enhanced Perls' iron staining of coronal sections of WT (6B-4) brains. Images (10–66) are from rostral to caudal. High resolution files are available from the corresponding author. (ZIP) [file pone.0098072.s012.zip › PerlsIronStain_WT/6B4-49.jpg]

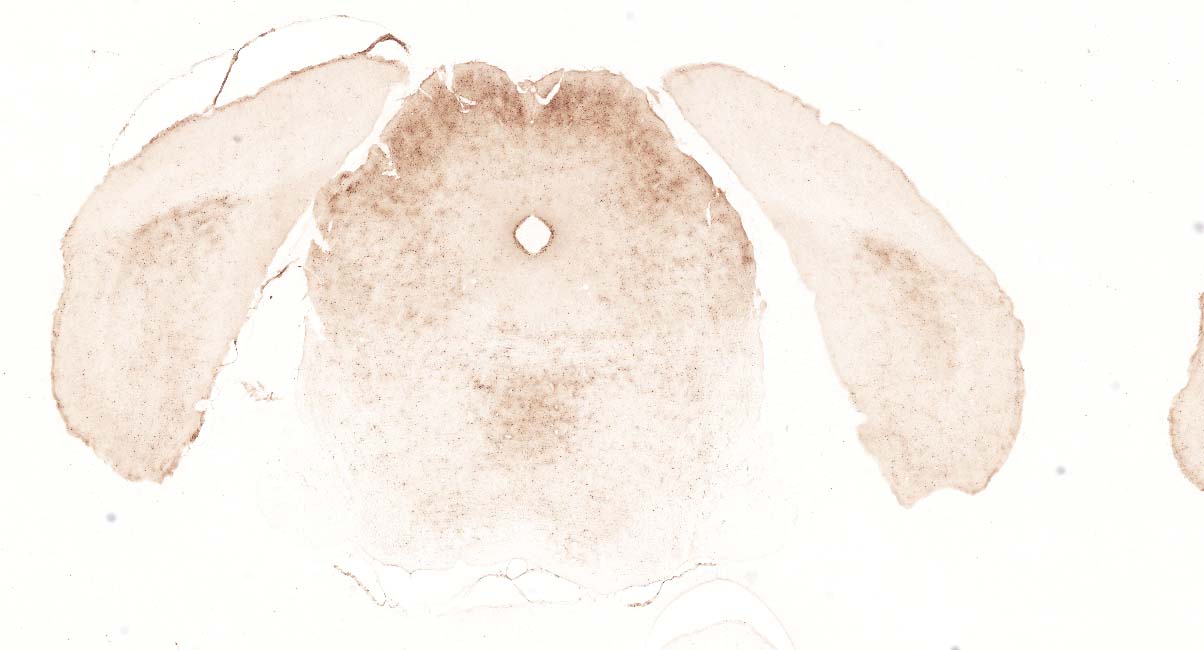

Supplement: File S1 — DAB-enhanced Perls' iron staining of coronal sections of WT (6B-4) brains. Images (10–66) are from rostral to caudal. High resolution files are available from the corresponding author. (ZIP) [file pone.0098072.s012.zip › PerlsIronStain_WT/6B4-50.jpg]

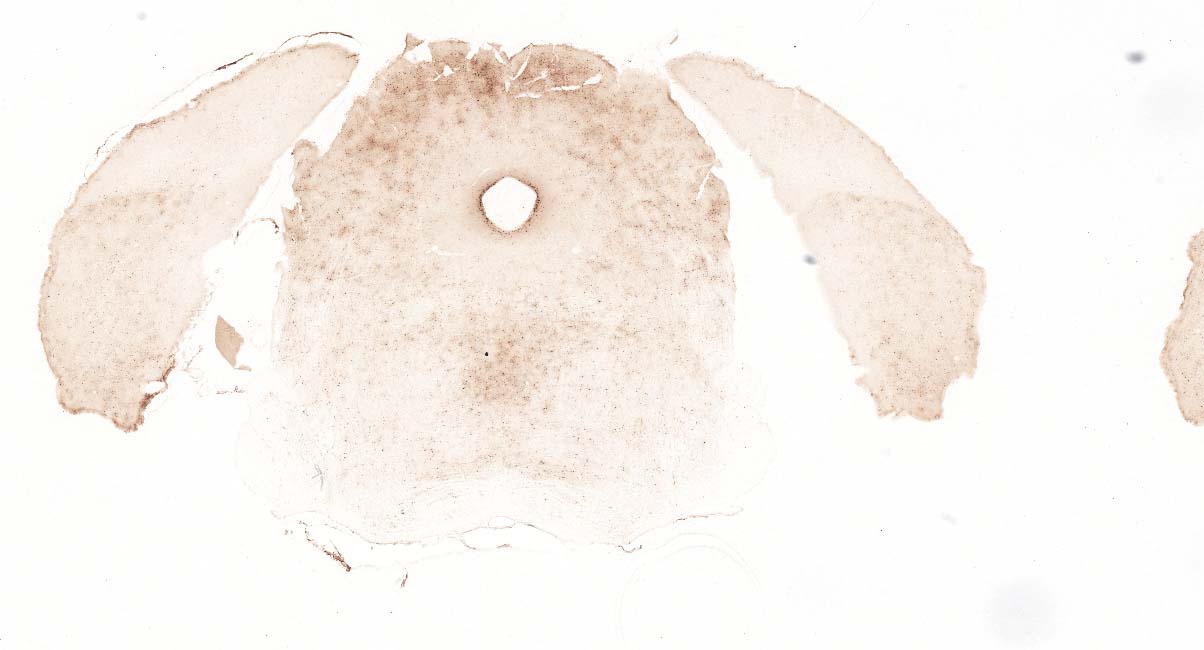

Supplement: File S1 — DAB-enhanced Perls' iron staining of coronal sections of WT (6B-4) brains. Images (10–66) are from rostral to caudal. High resolution files are available from the corresponding author. (ZIP) [file pone.0098072.s012.zip › PerlsIronStain_WT/6B4-51.jpg]

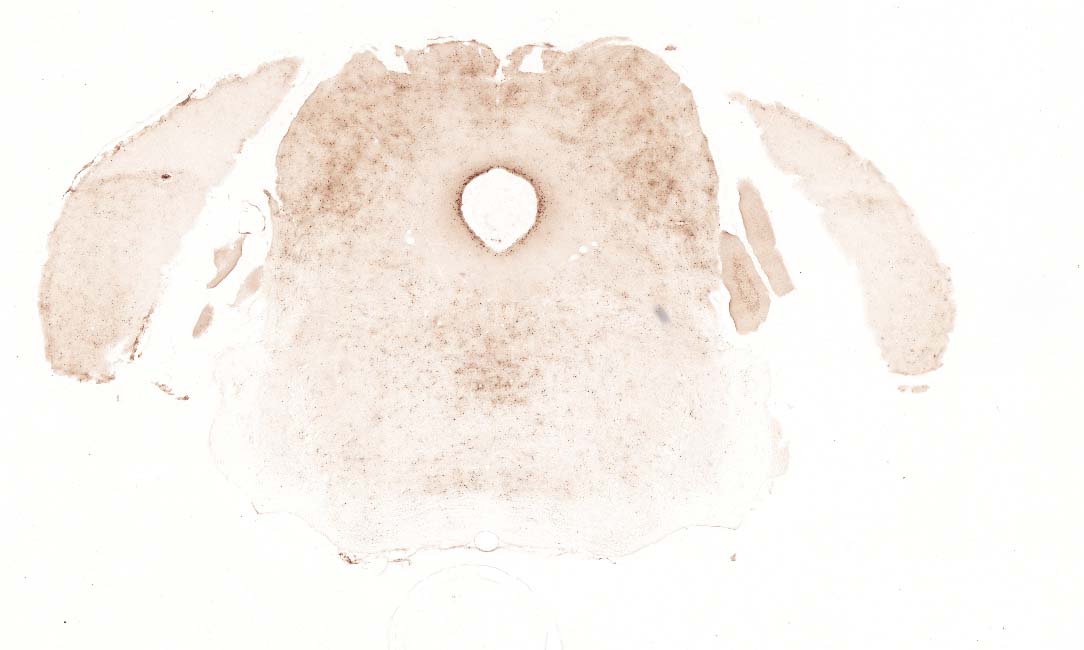

Supplement: File S1 — DAB-enhanced Perls' iron staining of coronal sections of WT (6B-4) brains. Images (10–66) are from rostral to caudal. High resolution files are available from the corresponding author. (ZIP) [file pone.0098072.s012.zip › PerlsIronStain_WT/6B4-52.jpg]

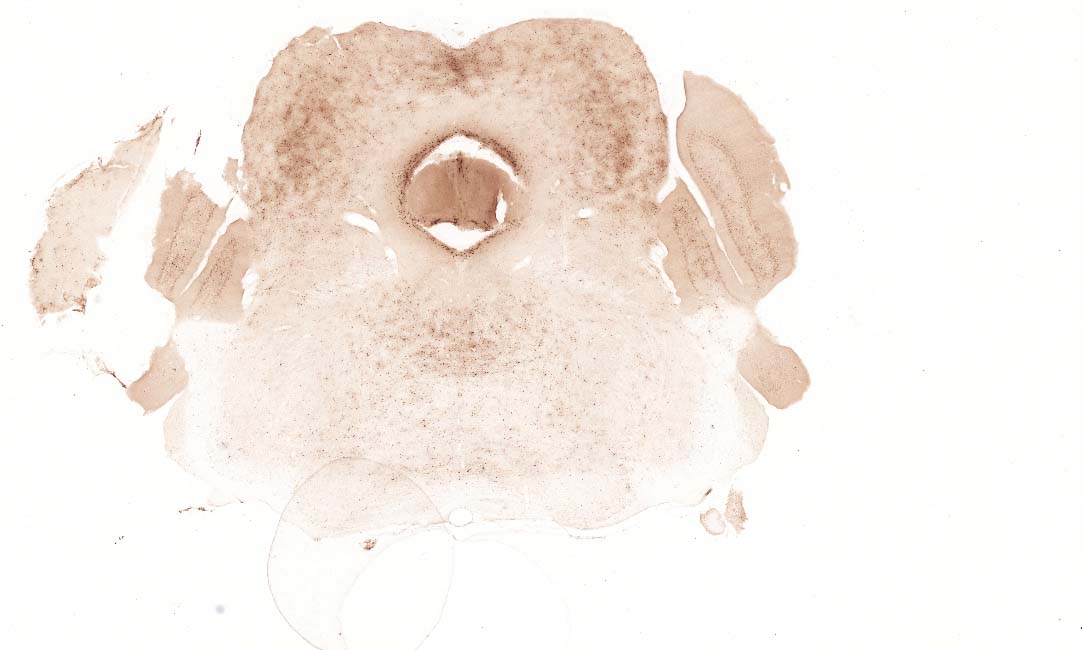

Supplement: File S1 — DAB-enhanced Perls' iron staining of coronal sections of WT (6B-4) brains. Images (10–66) are from rostral to caudal. High resolution files are available from the corresponding author. (ZIP) [file pone.0098072.s012.zip › PerlsIronStain_WT/6B4-53.jpg]

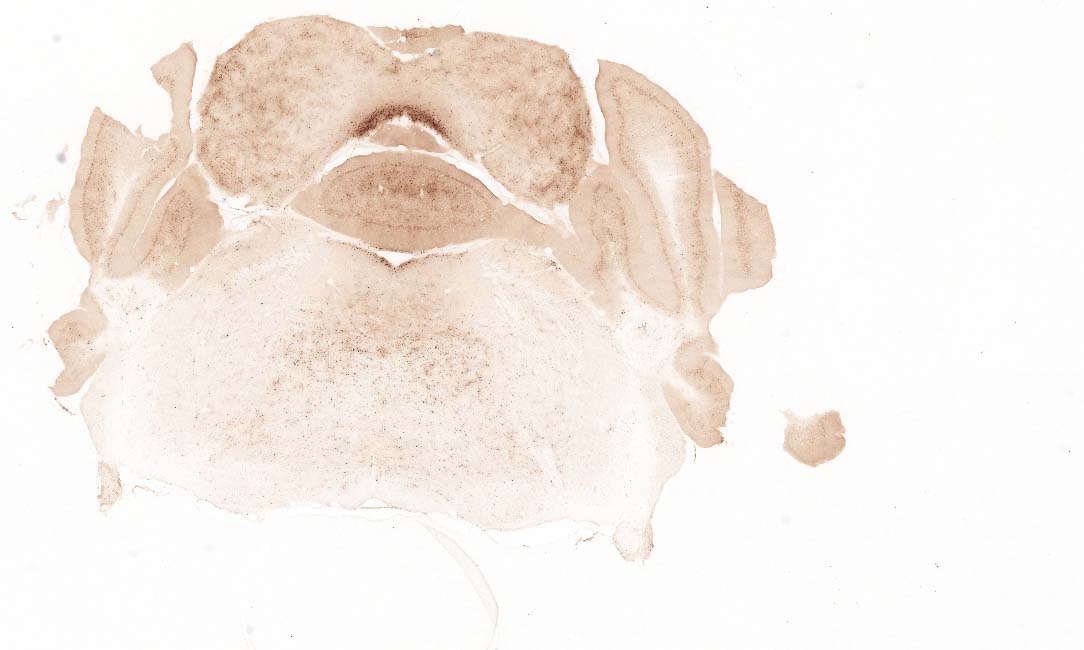

Supplement: File S1 — DAB-enhanced Perls' iron staining of coronal sections of WT (6B-4) brains. Images (10–66) are from rostral to caudal. High resolution files are available from the corresponding author. (ZIP) [file pone.0098072.s012.zip › PerlsIronStain_WT/6B4-54.jpg]

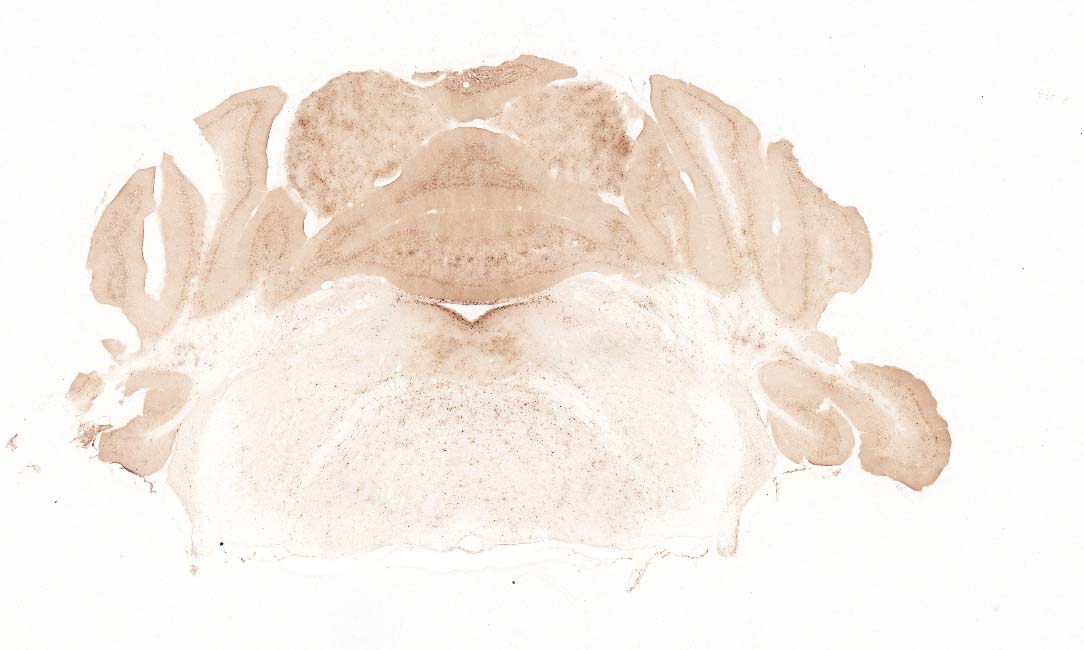

Supplement: File S1 — DAB-enhanced Perls' iron staining of coronal sections of WT (6B-4) brains. Images (10–66) are from rostral to caudal. High resolution files are available from the corresponding author. (ZIP) [file pone.0098072.s012.zip › PerlsIronStain_WT/6B4-55.jpg]

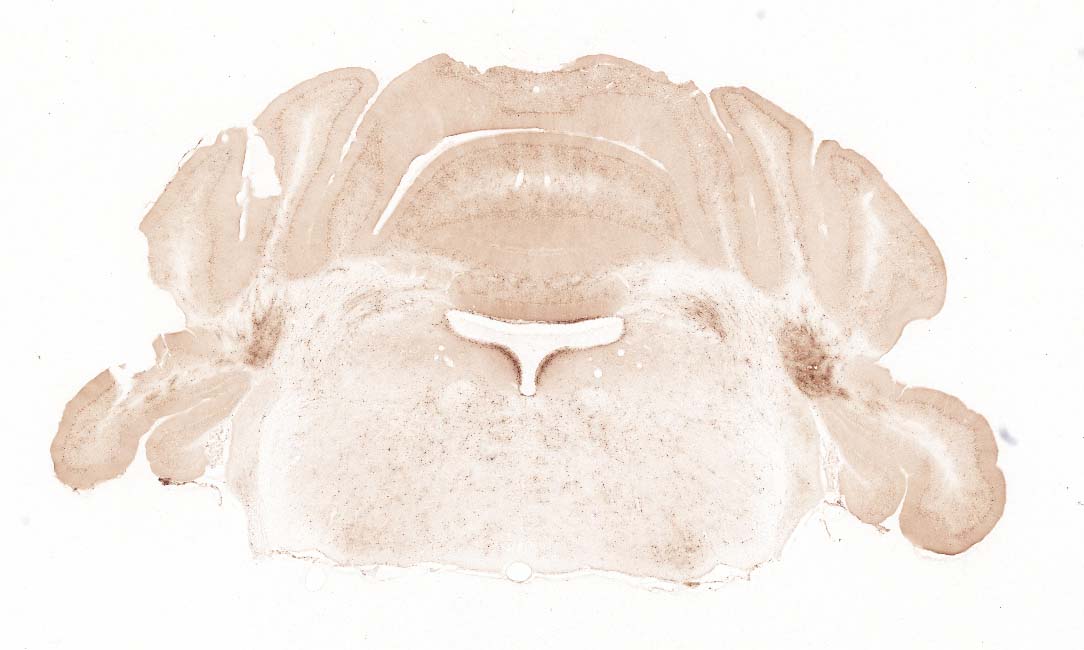

Supplement: File S1 — DAB-enhanced Perls' iron staining of coronal sections of WT (6B-4) brains. Images (10–66) are from rostral to caudal. High resolution files are available from the corresponding author. (ZIP) [file pone.0098072.s012.zip › PerlsIronStain_WT/6B4-56.jpg]

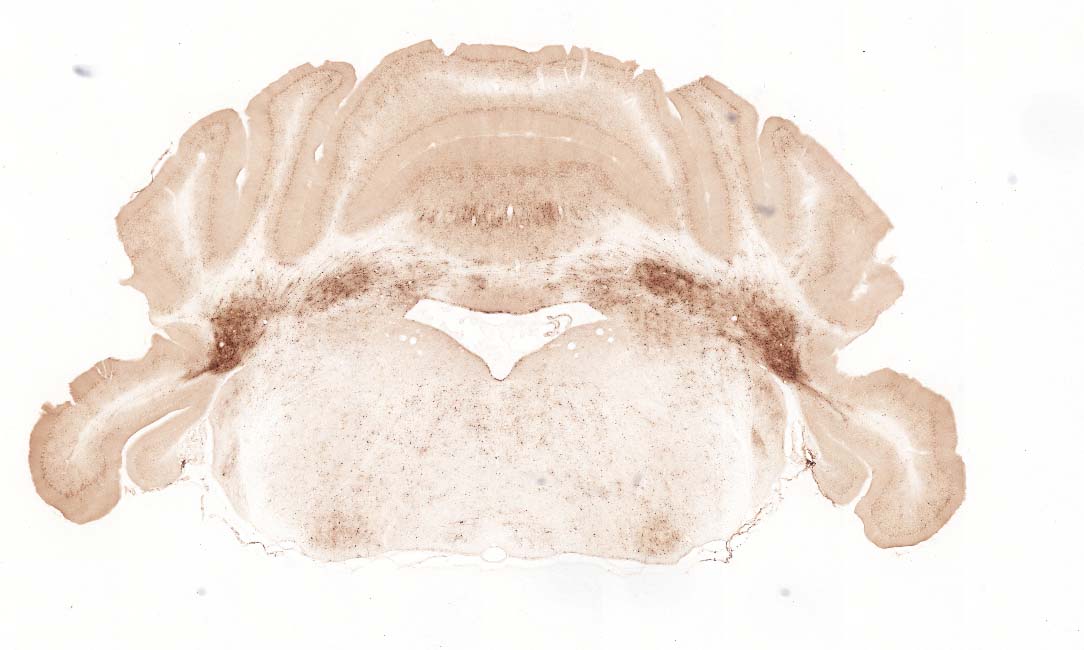

Supplement: File S1 — DAB-enhanced Perls' iron staining of coronal sections of WT (6B-4) brains. Images (10–66) are from rostral to caudal. High resolution files are available from the corresponding author. (ZIP) [file pone.0098072.s012.zip › PerlsIronStain_WT/6B4-57.jpg]

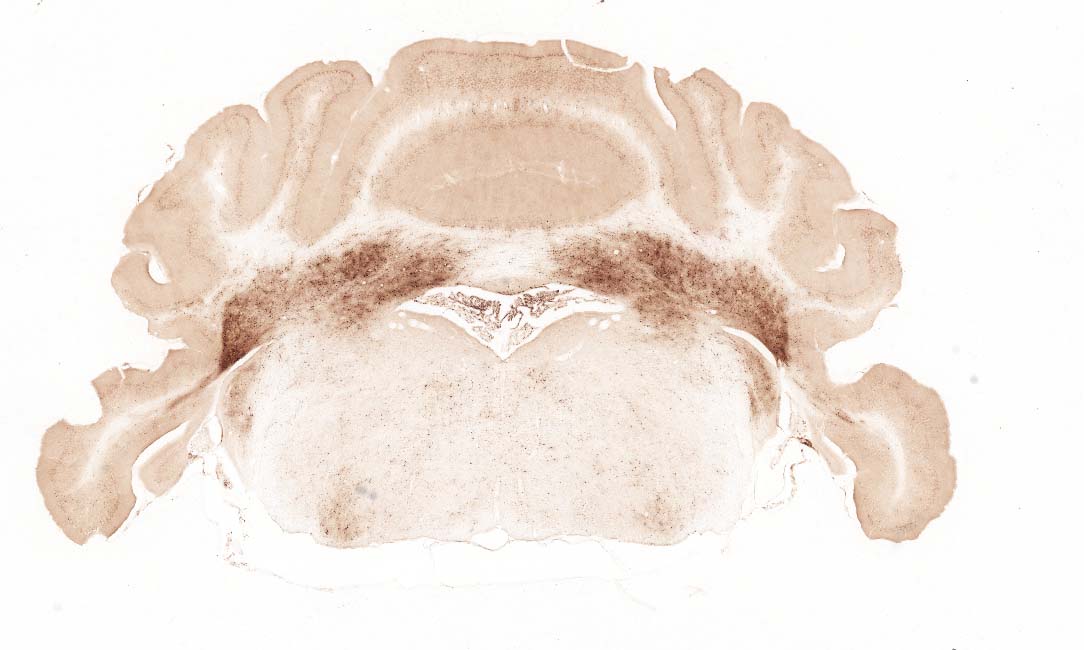

Supplement: File S1 — DAB-enhanced Perls' iron staining of coronal sections of WT (6B-4) brains. Images (10–66) are from rostral to caudal. High resolution files are available from the corresponding author. (ZIP) [file pone.0098072.s012.zip › PerlsIronStain_WT/6B4-58.jpg]

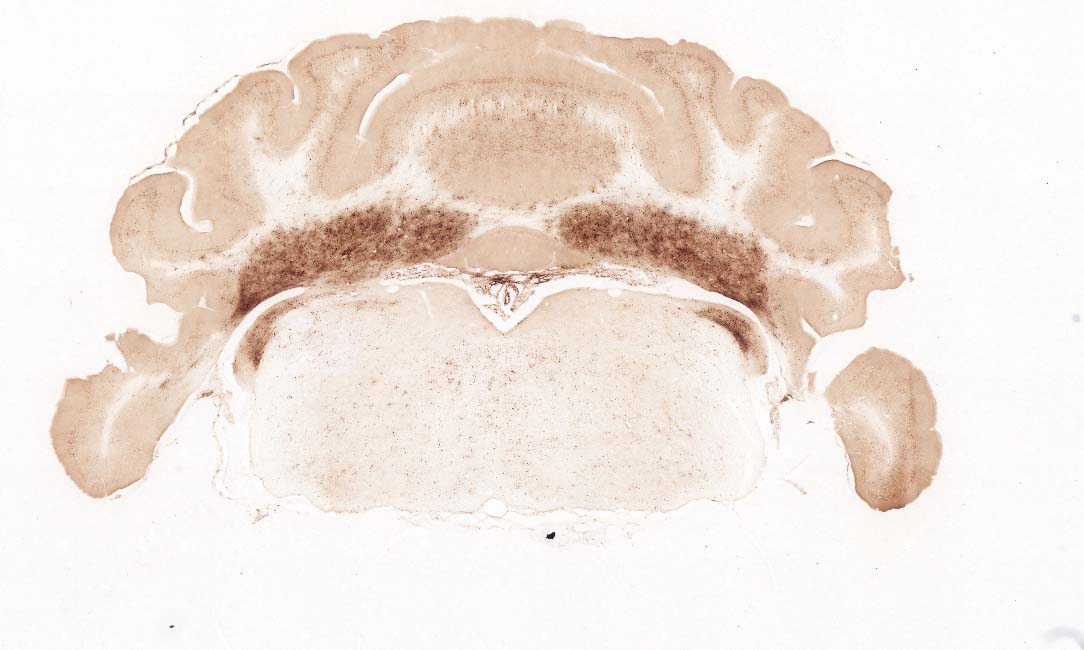

Supplement: File S1 — DAB-enhanced Perls' iron staining of coronal sections of WT (6B-4) brains. Images (10–66) are from rostral to caudal. High resolution files are available from the corresponding author. (ZIP) [file pone.0098072.s012.zip › PerlsIronStain_WT/6B4-59.jpg]

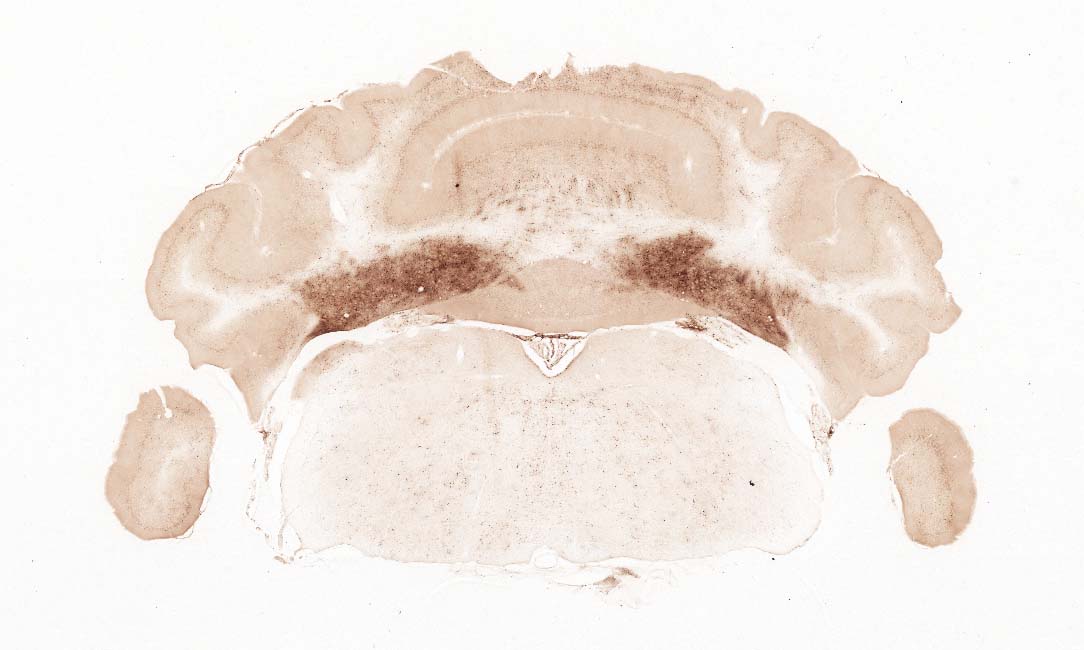

Supplement: File S1 — DAB-enhanced Perls' iron staining of coronal sections of WT (6B-4) brains. Images (10–66) are from rostral to caudal. High resolution files are available from the corresponding author. (ZIP) [file pone.0098072.s012.zip › PerlsIronStain_WT/6B4-60.jpg]

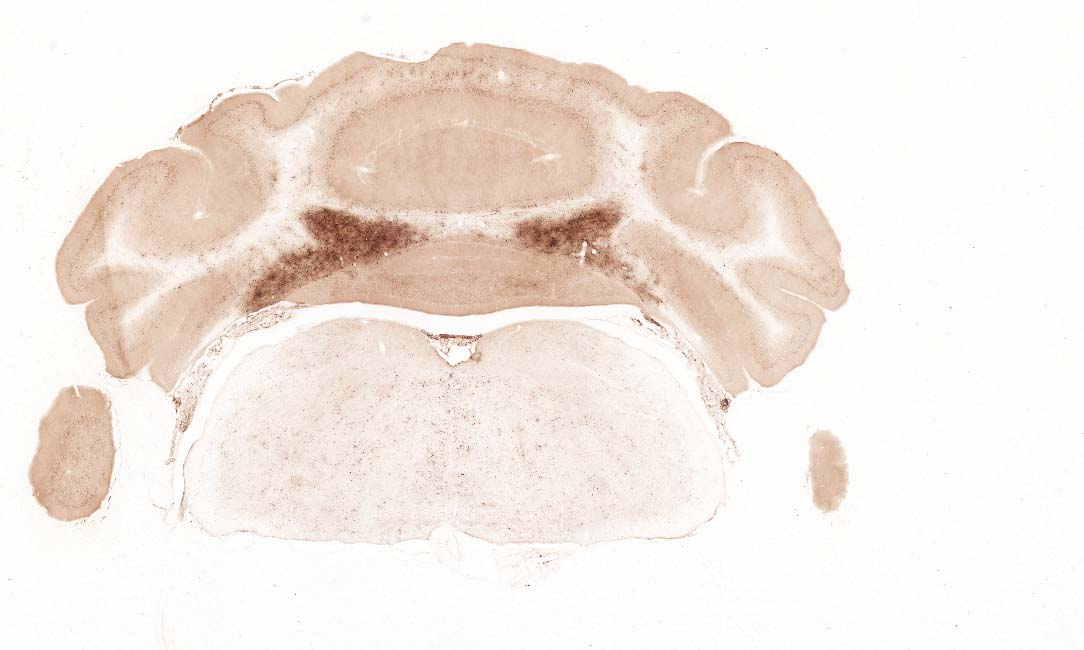

Supplement: File S1 — DAB-enhanced Perls' iron staining of coronal sections of WT (6B-4) brains. Images (10–66) are from rostral to caudal. High resolution files are available from the corresponding author. (ZIP) [file pone.0098072.s012.zip › PerlsIronStain_WT/6B4-61.jpg]

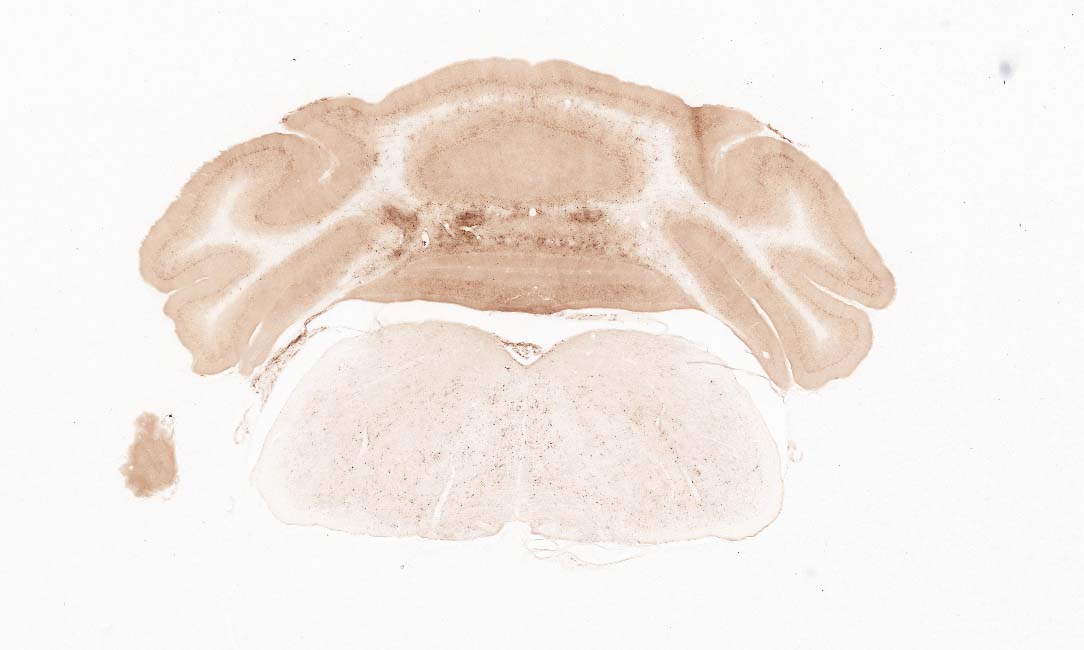

Supplement: File S1 — DAB-enhanced Perls' iron staining of coronal sections of WT (6B-4) brains. Images (10–66) are from rostral to caudal. High resolution files are available from the corresponding author. (ZIP) [file pone.0098072.s012.zip › PerlsIronStain_WT/6B4-62.jpg]

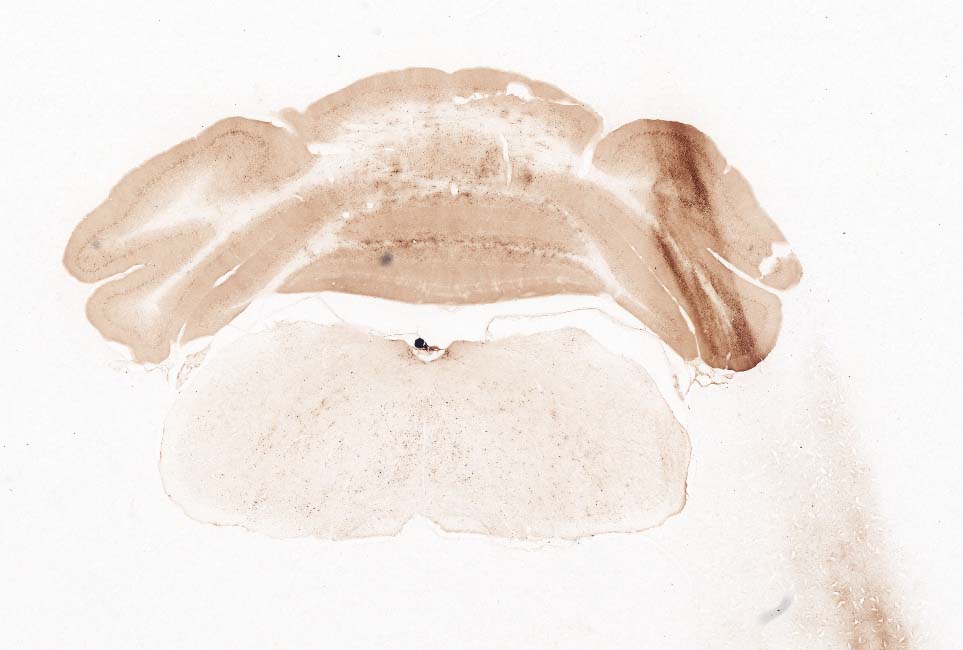

Supplement: File S1 — DAB-enhanced Perls' iron staining of coronal sections of WT (6B-4) brains. Images (10–66) are from rostral to caudal. High resolution files are available from the corresponding author. (ZIP) [file pone.0098072.s012.zip › PerlsIronStain_WT/6B4-63.jpg]

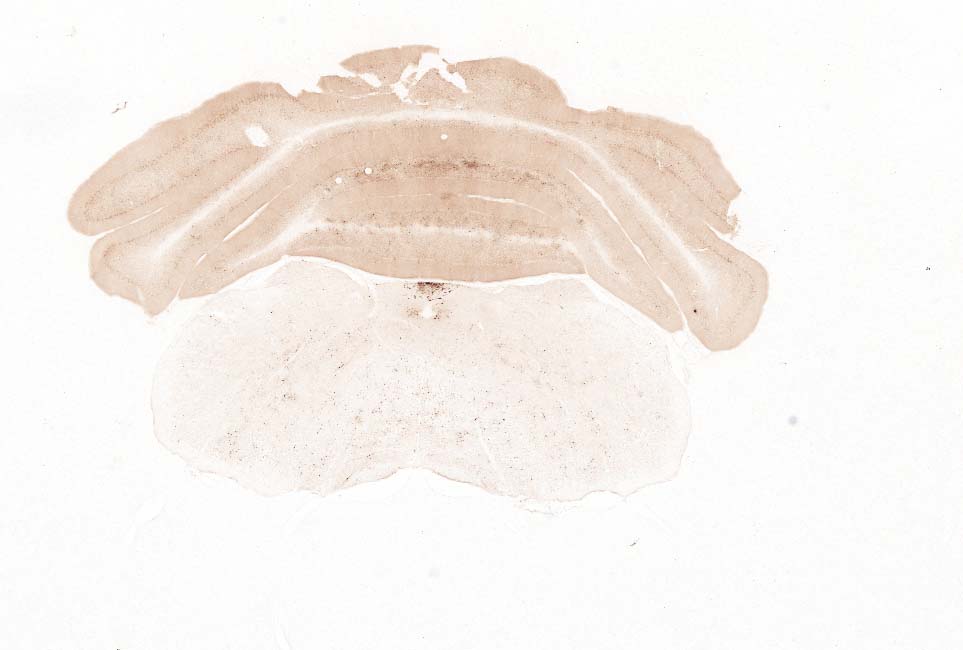

Supplement: File S1 — DAB-enhanced Perls' iron staining of coronal sections of WT (6B-4) brains. Images (10–66) are from rostral to caudal. High resolution files are available from the corresponding author. (ZIP) [file pone.0098072.s012.zip › PerlsIronStain_WT/6B4-64.jpg]

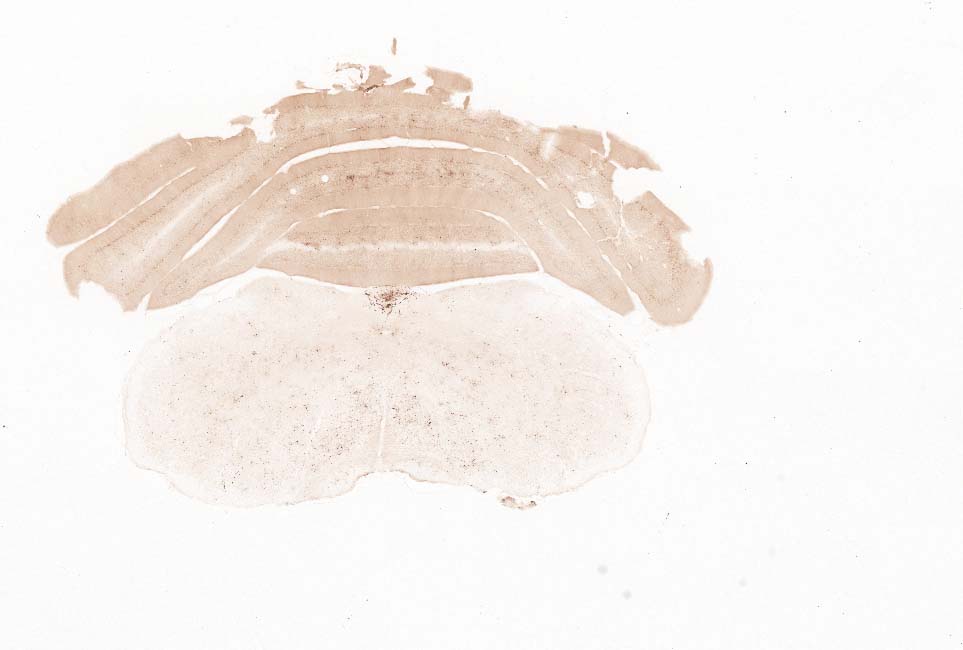

Supplement: File S1 — DAB-enhanced Perls' iron staining of coronal sections of WT (6B-4) brains. Images (10–66) are from rostral to caudal. High resolution files are available from the corresponding author. (ZIP) [file pone.0098072.s012.zip › PerlsIronStain_WT/6B4-65.jpg]

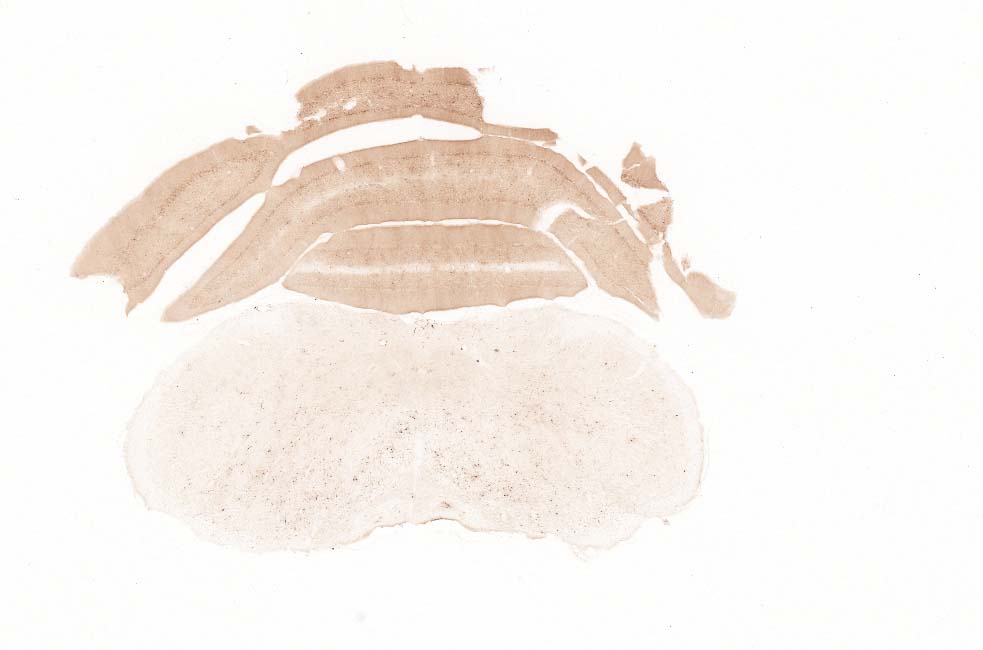

Supplement: File S1 — DAB-enhanced Perls' iron staining of coronal sections of WT (6B-4) brains. Images (10–66) are from rostral to caudal. High resolution files are available from the corresponding author. (ZIP) [file pone.0098072.s012.zip › PerlsIronStain_WT/6B4-66.jpg]

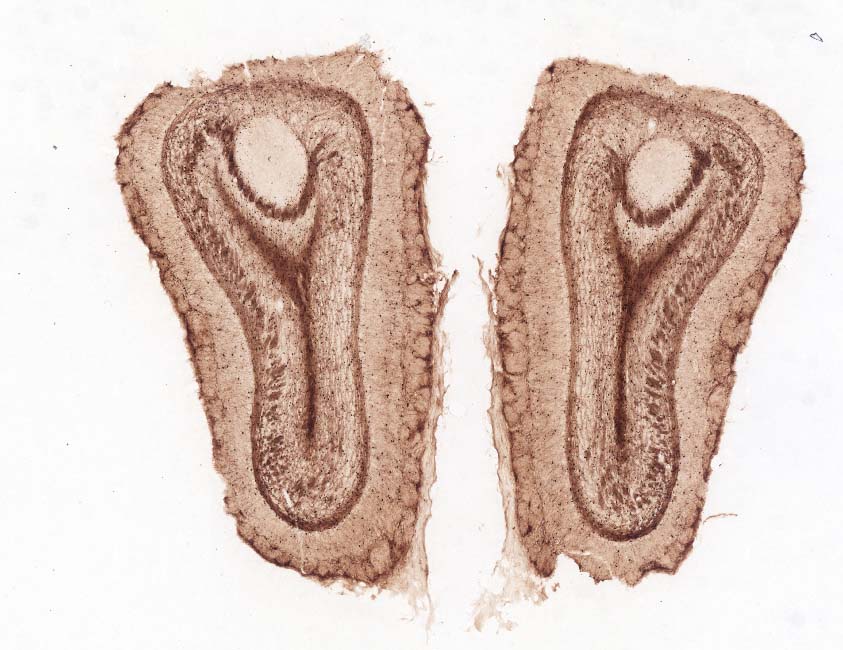

Supplement: File S2 — DAB-enhanced Perls' iron staining of coronal sections of Irp2−/− (6A-3) brains. Images (10–66) are from rostral to caudal. High resolution files are available from the corresponding author. (ZIP) [file pone.0098072.s013.zip › PerlsIronStain_IRP2KO/6A3-10.jpg]

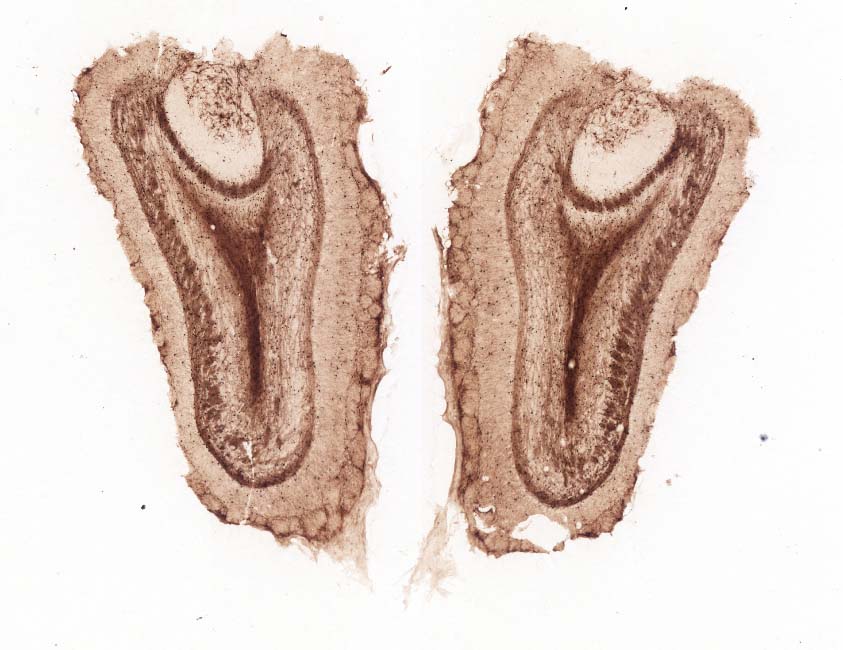

Supplement: File S2 — DAB-enhanced Perls' iron staining of coronal sections of Irp2−/− (6A-3) brains. Images (10–66) are from rostral to caudal. High resolution files are available from the corresponding author. (ZIP) [file pone.0098072.s013.zip › PerlsIronStain_IRP2KO/6A3-11.jpg]

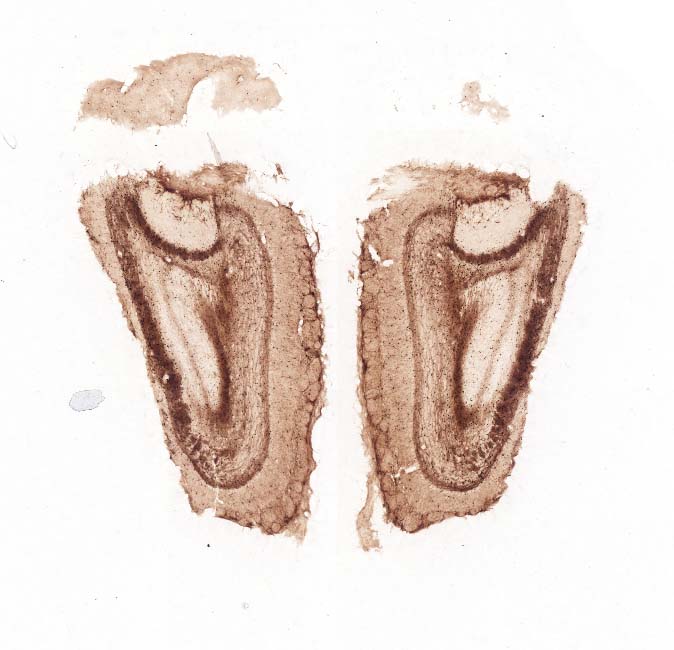

Supplement: File S2 — DAB-enhanced Perls' iron staining of coronal sections of Irp2−/− (6A-3) brains. Images (10–66) are from rostral to caudal. High resolution files are available from the corresponding author. (ZIP) [file pone.0098072.s013.zip › PerlsIronStain_IRP2KO/6A3-12.jpg]

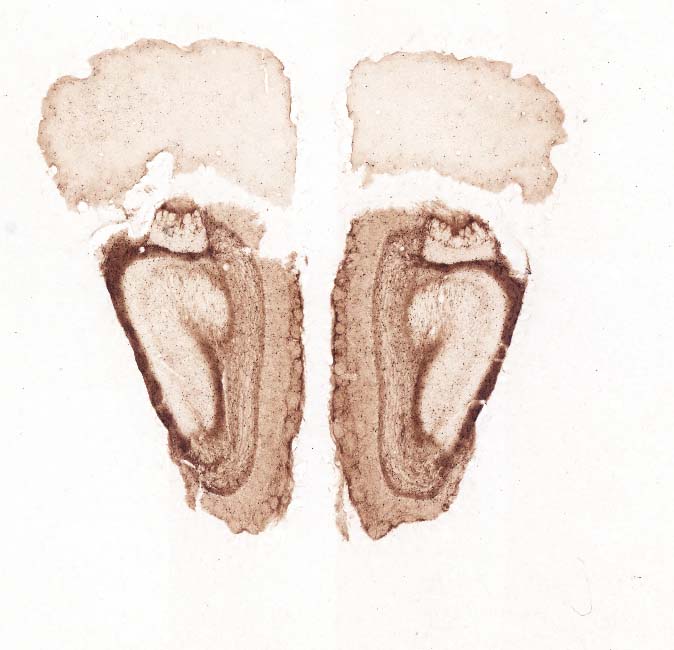

Supplement: File S2 — DAB-enhanced Perls' iron staining of coronal sections of Irp2−/− (6A-3) brains. Images (10–66) are from rostral to caudal. High resolution files are available from the corresponding author. (ZIP) [file pone.0098072.s013.zip › PerlsIronStain_IRP2KO/6A3-13.jpg]

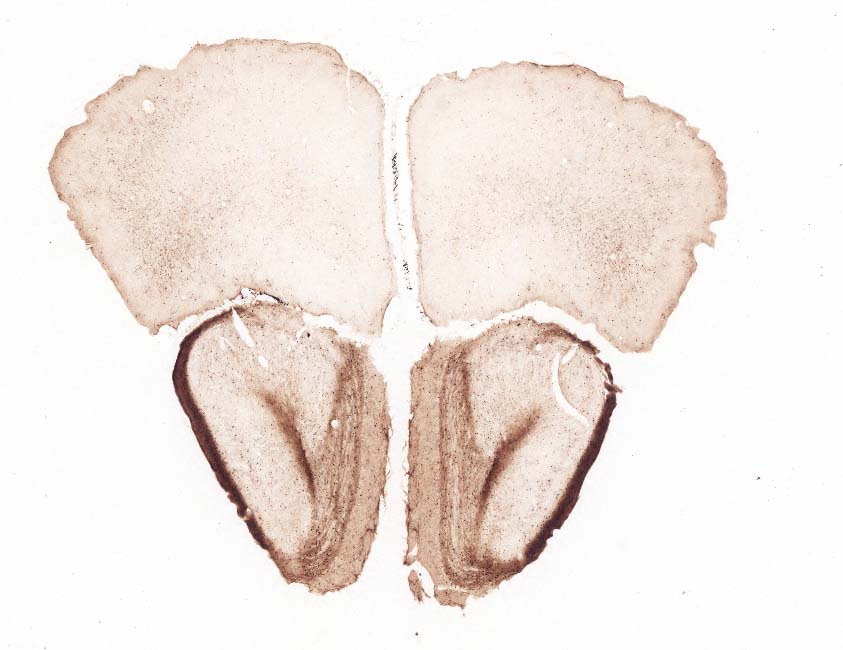

Supplement: File S2 — DAB-enhanced Perls' iron staining of coronal sections of Irp2−/− (6A-3) brains. Images (10–66) are from rostral to caudal. High resolution files are available from the corresponding author. (ZIP) [file pone.0098072.s013.zip › PerlsIronStain_IRP2KO/6A3-14.jpg]

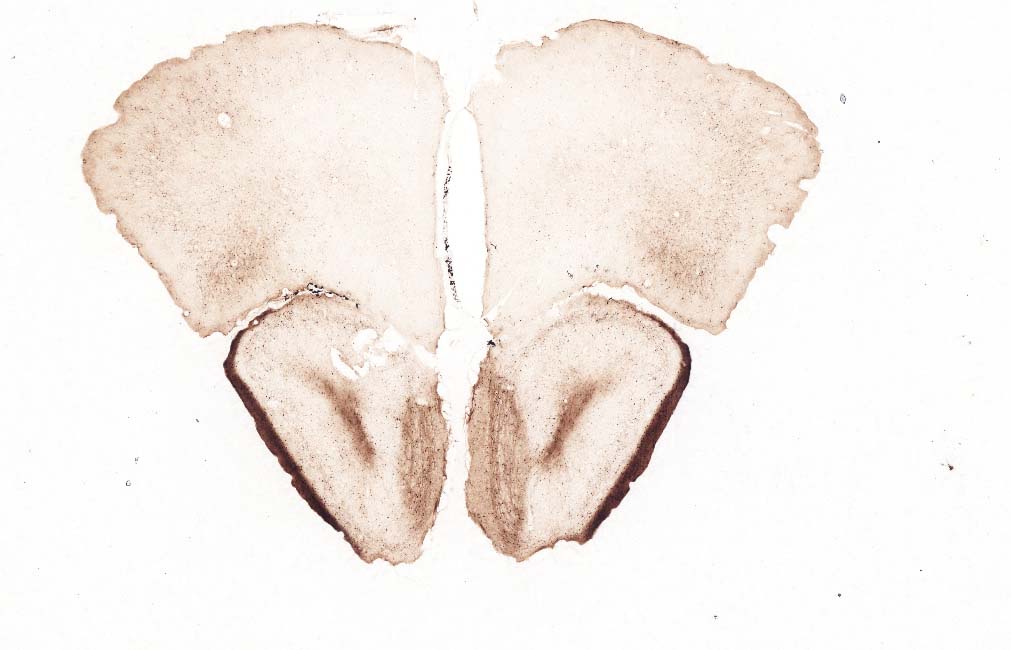

Supplement: File S2 — DAB-enhanced Perls' iron staining of coronal sections of Irp2−/− (6A-3) brains. Images (10–66) are from rostral to caudal. High resolution files are available from the corresponding author. (ZIP) [file pone.0098072.s013.zip › PerlsIronStain_IRP2KO/6A3-15.jpg]

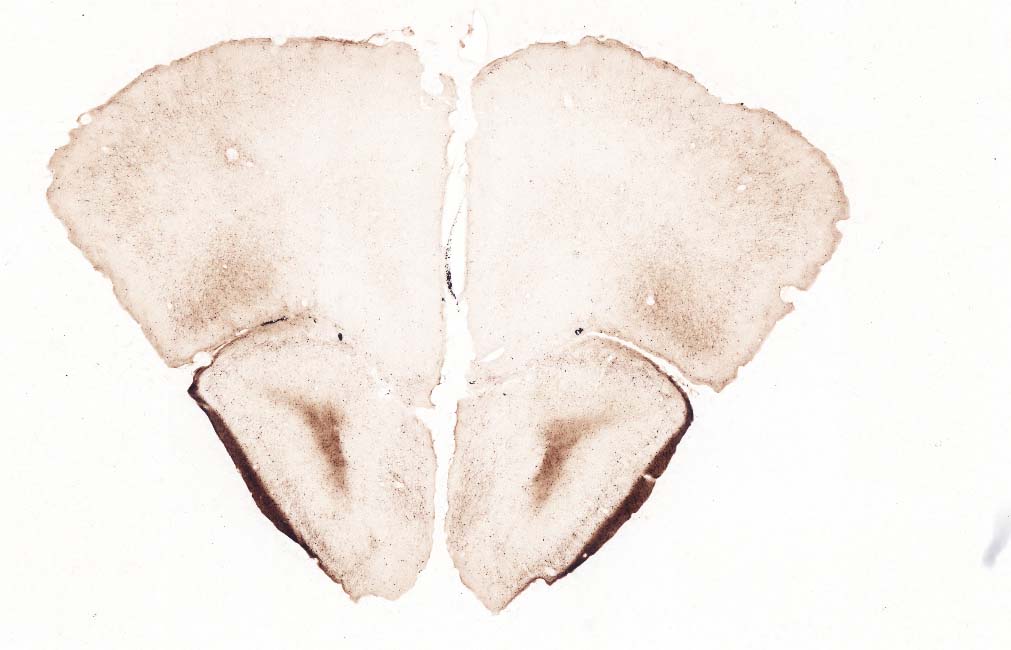

Supplement: File S2 — DAB-enhanced Perls' iron staining of coronal sections of Irp2−/− (6A-3) brains. Images (10–66) are from rostral to caudal. High resolution files are available from the corresponding author. (ZIP) [file pone.0098072.s013.zip › PerlsIronStain_IRP2KO/6A3-16.jpg]

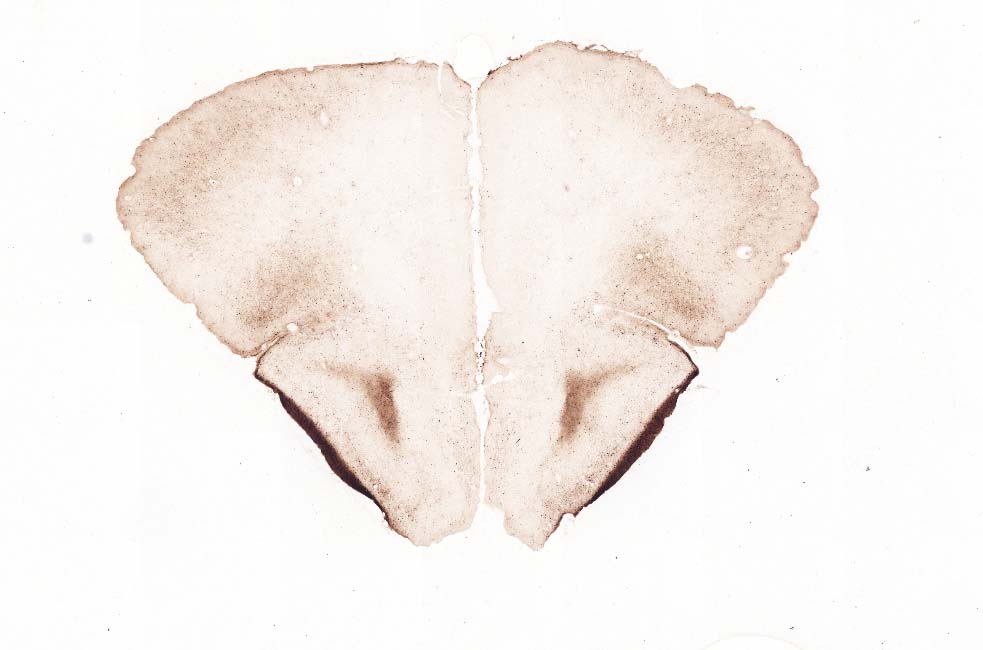

Supplement: File S2 — DAB-enhanced Perls' iron staining of coronal sections of Irp2−/− (6A-3) brains. Images (10–66) are from rostral to caudal. High resolution files are available from the corresponding author. (ZIP) [file pone.0098072.s013.zip › PerlsIronStain_IRP2KO/6A3-17.jpg]

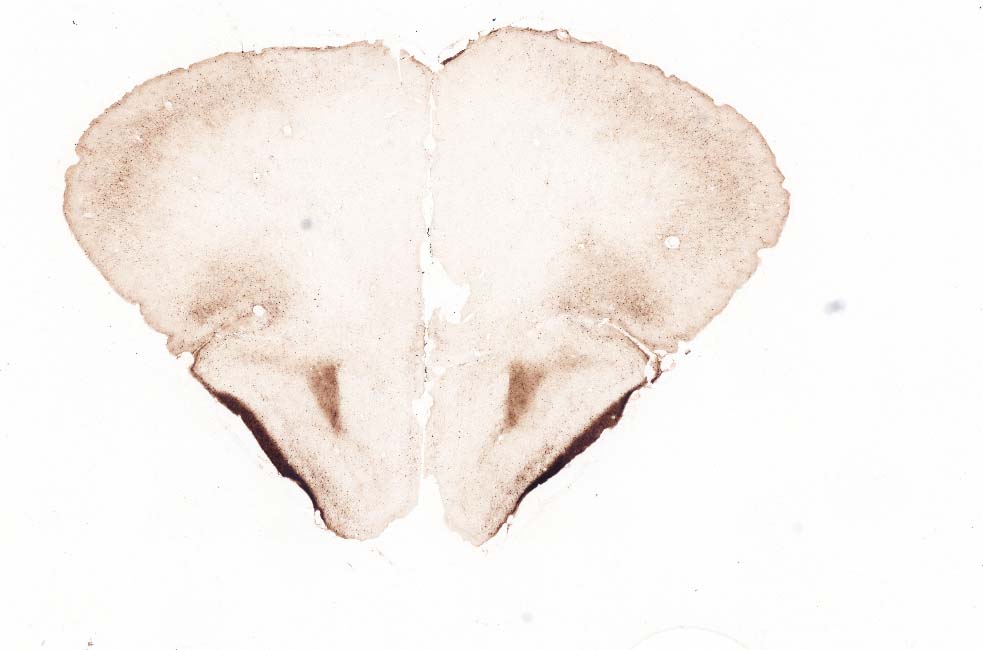

Supplement: File S2 — DAB-enhanced Perls' iron staining of coronal sections of Irp2−/− (6A-3) brains. Images (10–66) are from rostral to caudal. High resolution files are available from the corresponding author. (ZIP) [file pone.0098072.s013.zip › PerlsIronStain_IRP2KO/6A3-18.jpg]

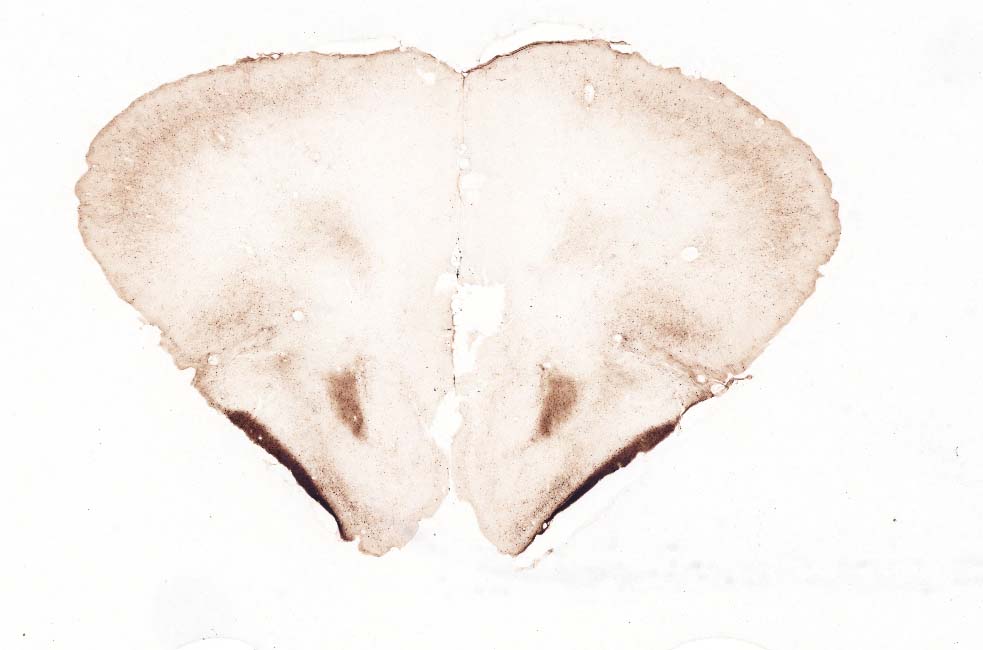

Supplement: File S2 — DAB-enhanced Perls' iron staining of coronal sections of Irp2−/− (6A-3) brains. Images (10–66) are from rostral to caudal. High resolution files are available from the corresponding author. (ZIP) [file pone.0098072.s013.zip › PerlsIronStain_IRP2KO/6A3-19.jpg]

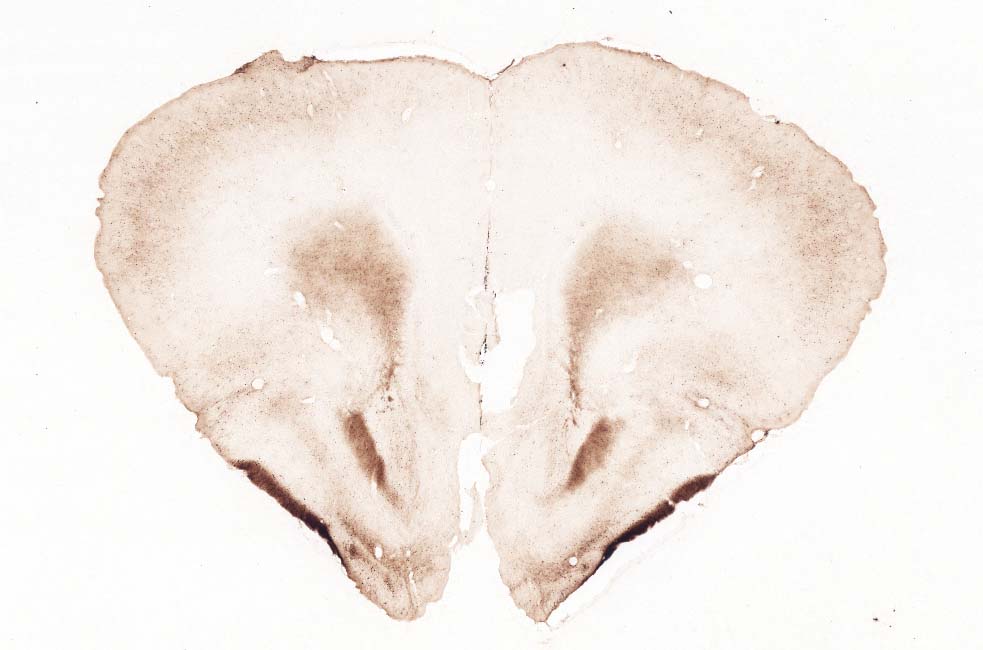

Supplement: File S2 — DAB-enhanced Perls' iron staining of coronal sections of Irp2−/− (6A-3) brains. Images (10–66) are from rostral to caudal. High resolution files are available from the corresponding author. (ZIP) [file pone.0098072.s013.zip › PerlsIronStain_IRP2KO/6A3-20.jpg]

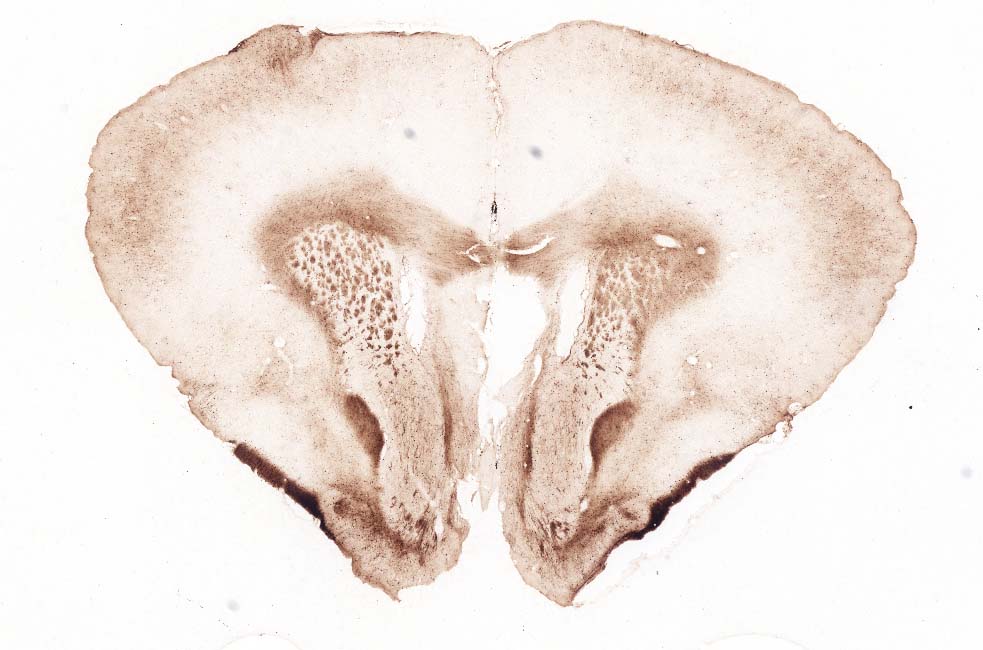

Supplement: File S2 — DAB-enhanced Perls' iron staining of coronal sections of Irp2−/− (6A-3) brains. Images (10–66) are from rostral to caudal. High resolution files are available from the corresponding author. (ZIP) [file pone.0098072.s013.zip › PerlsIronStain_IRP2KO/6A3-21.jpg]

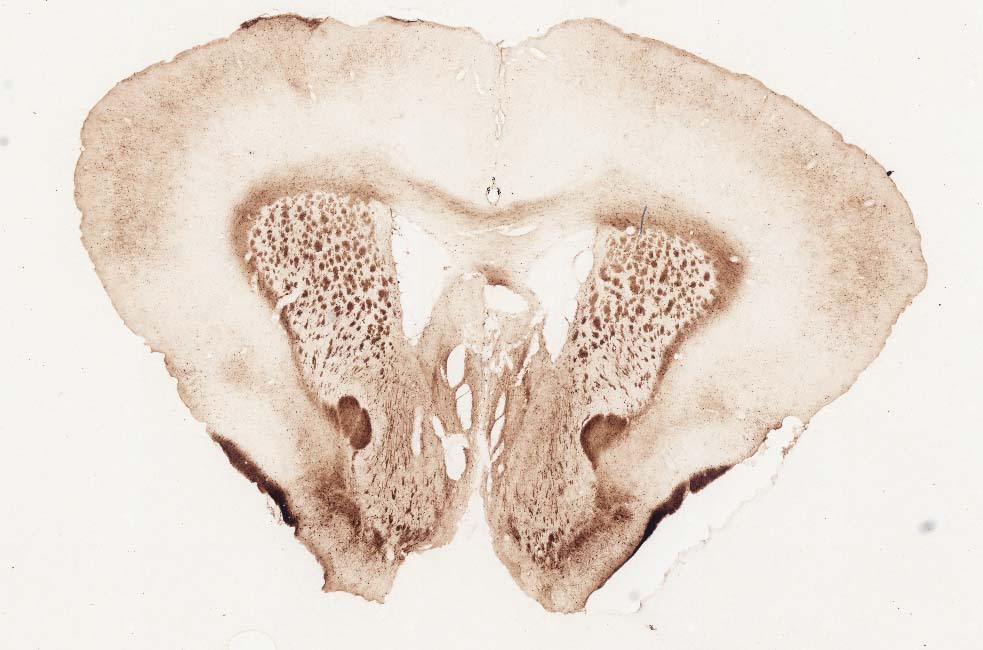

Supplement: File S2 — DAB-enhanced Perls' iron staining of coronal sections of Irp2−/− (6A-3) brains. Images (10–66) are from rostral to caudal. High resolution files are available from the corresponding author. (ZIP) [file pone.0098072.s013.zip › PerlsIronStain_IRP2KO/6A3-22.jpg]

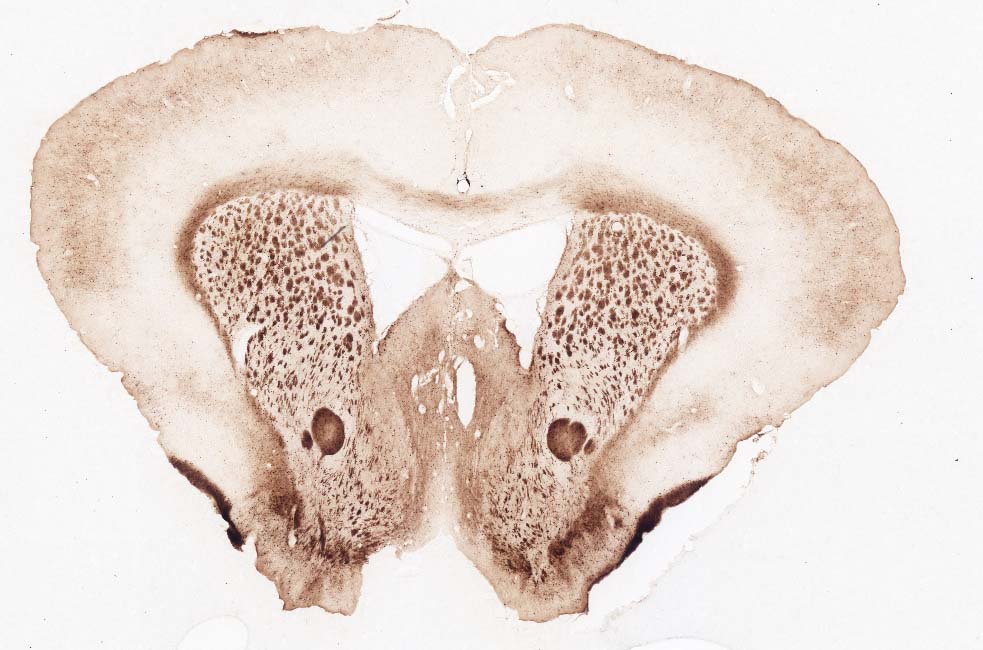

Supplement: File S2 — DAB-enhanced Perls' iron staining of coronal sections of Irp2−/− (6A-3) brains. Images (10–66) are from rostral to caudal. High resolution files are available from the corresponding author. (ZIP) [file pone.0098072.s013.zip › PerlsIronStain_IRP2KO/6A3-23.jpg]

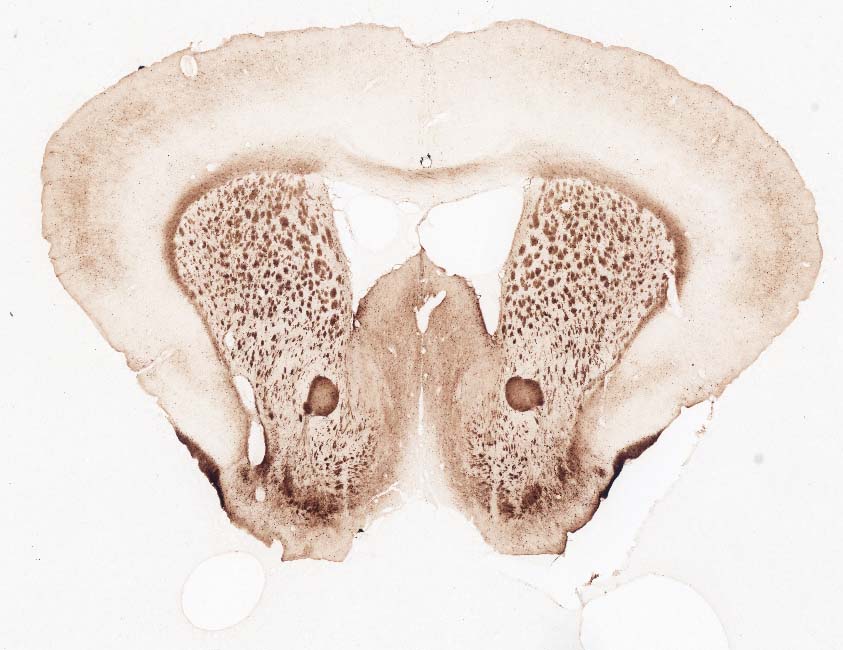

Supplement: File S2 — DAB-enhanced Perls' iron staining of coronal sections of Irp2−/− (6A-3) brains. Images (10–66) are from rostral to caudal. High resolution files are available from the corresponding author. (ZIP) [file pone.0098072.s013.zip › PerlsIronStain_IRP2KO/6A3-24.jpg]

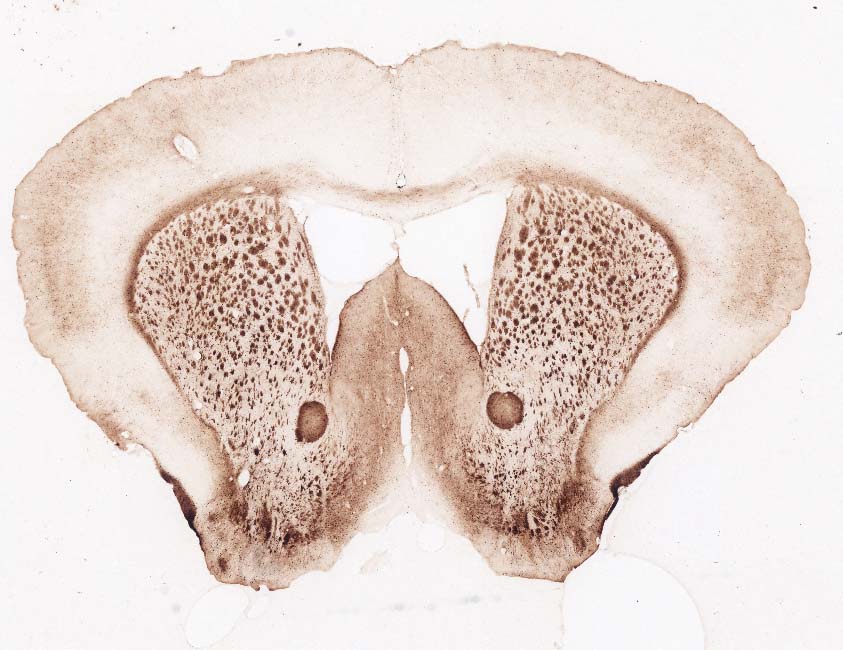

Supplement: File S2 — DAB-enhanced Perls' iron staining of coronal sections of Irp2−/− (6A-3) brains. Images (10–66) are from rostral to caudal. High resolution files are available from the corresponding author. (ZIP) [file pone.0098072.s013.zip › PerlsIronStain_IRP2KO/6A3-25.jpg]

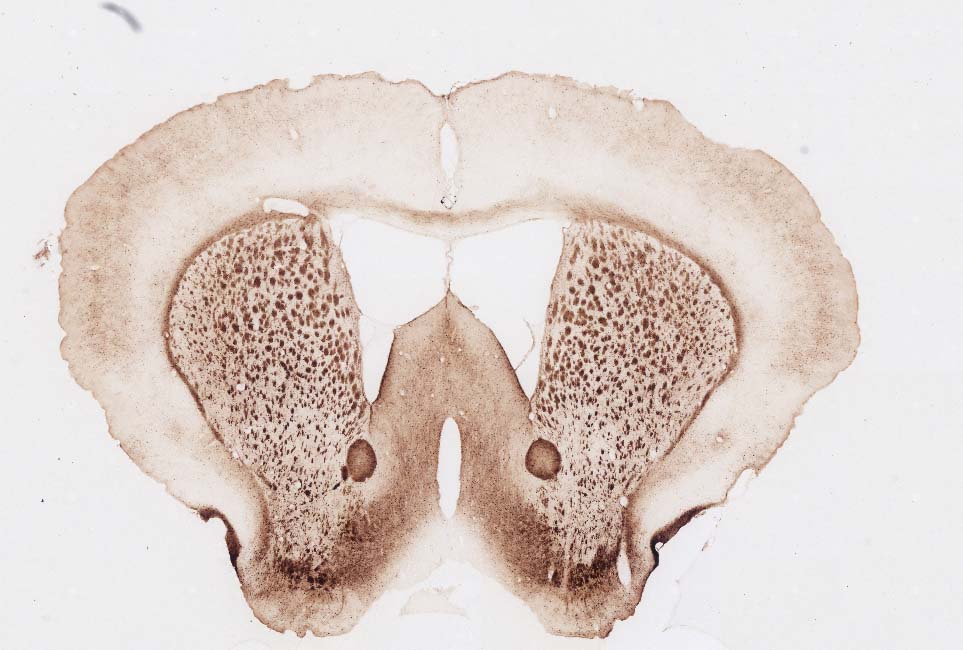

Supplement: File S2 — DAB-enhanced Perls' iron staining of coronal sections of Irp2−/− (6A-3) brains. Images (10–66) are from rostral to caudal. High resolution files are available from the corresponding author. (ZIP) [file pone.0098072.s013.zip › PerlsIronStain_IRP2KO/6A3-26.jpg]

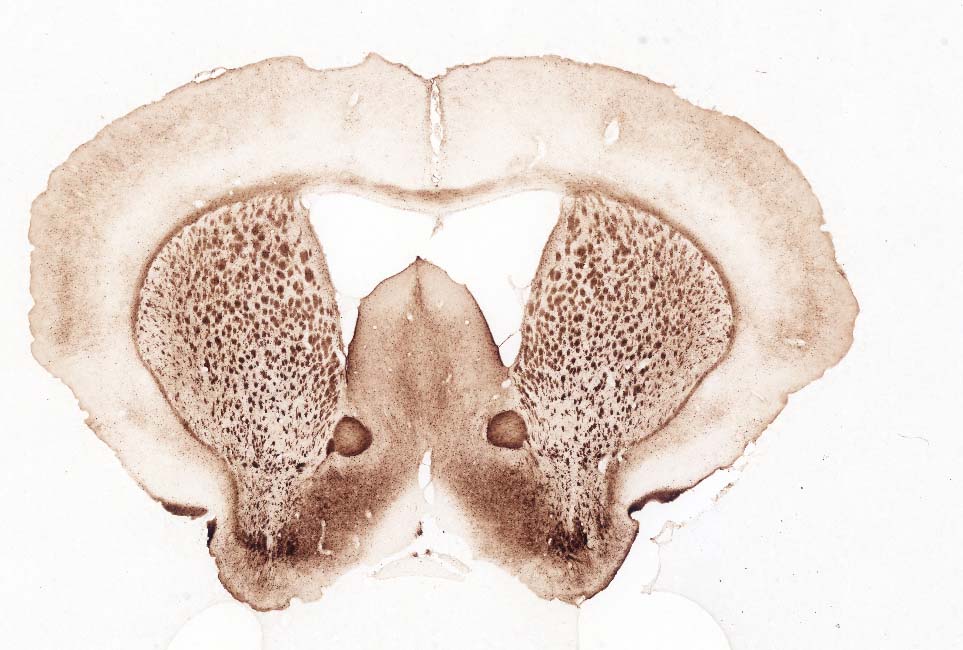

Supplement: File S2 — DAB-enhanced Perls' iron staining of coronal sections of Irp2−/− (6A-3) brains. Images (10–66) are from rostral to caudal. High resolution files are available from the corresponding author. (ZIP) [file pone.0098072.s013.zip › PerlsIronStain_IRP2KO/6A3-27.jpg]

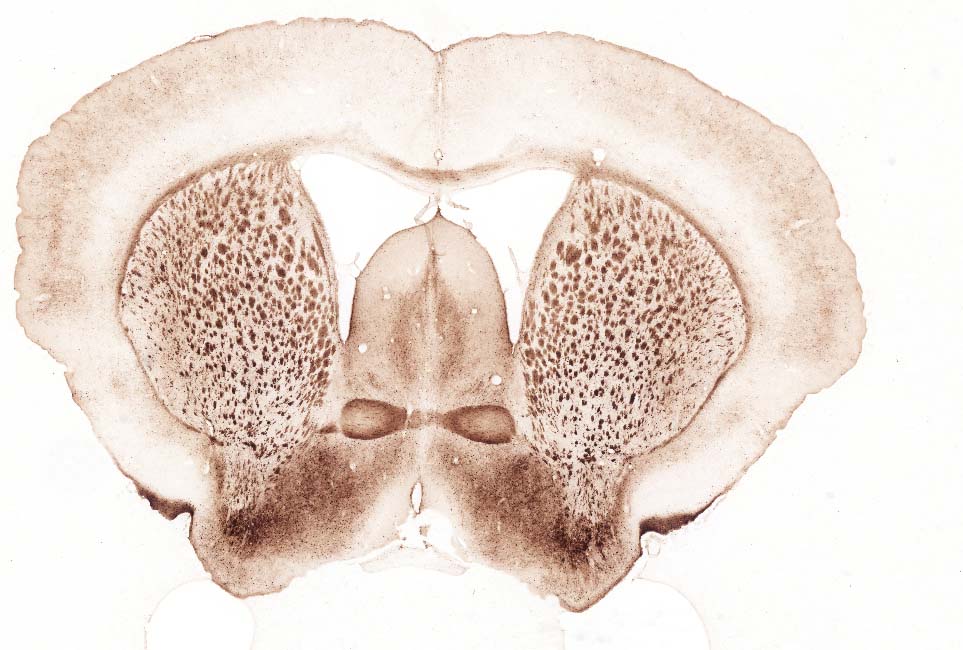

Supplement: File S2 — DAB-enhanced Perls' iron staining of coronal sections of Irp2−/− (6A-3) brains. Images (10–66) are from rostral to caudal. High resolution files are available from the corresponding author. (ZIP) [file pone.0098072.s013.zip › PerlsIronStain_IRP2KO/6A3-28.jpg]

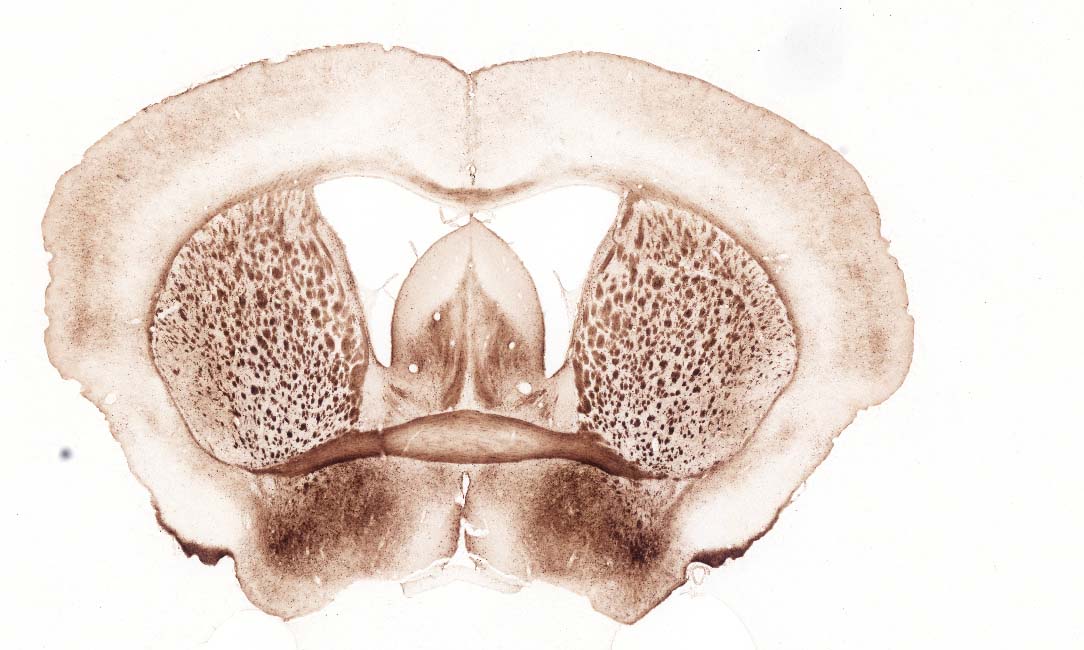

Supplement: File S2 — DAB-enhanced Perls' iron staining of coronal sections of Irp2−/− (6A-3) brains. Images (10–66) are from rostral to caudal. High resolution files are available from the corresponding author. (ZIP) [file pone.0098072.s013.zip › PerlsIronStain_IRP2KO/6A3-29.jpg]

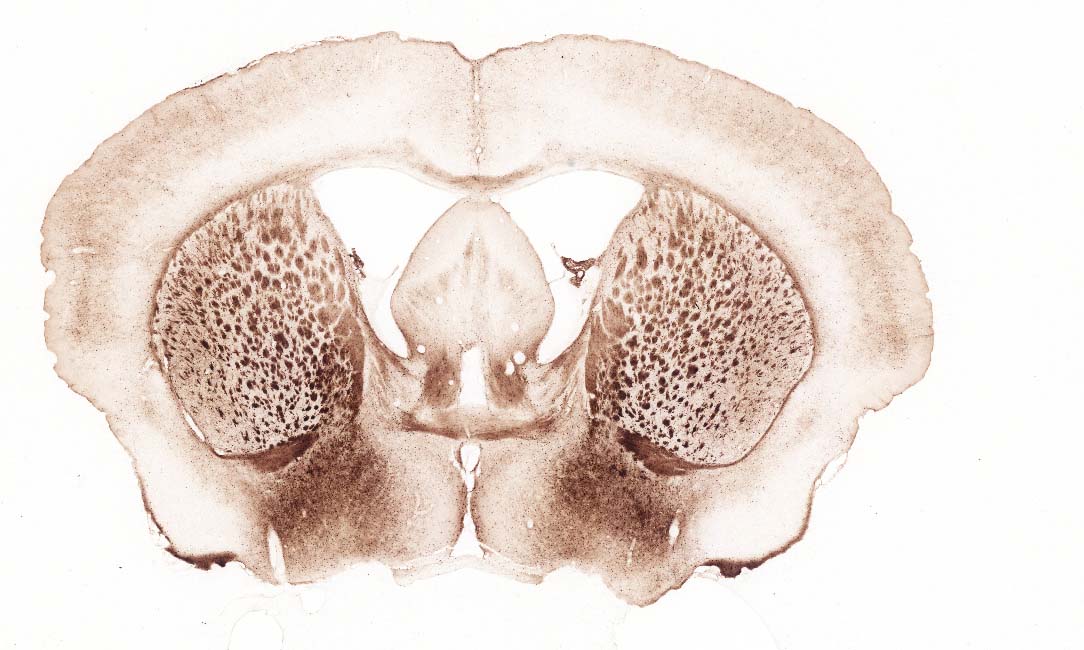

Supplement: File S2 — DAB-enhanced Perls' iron staining of coronal sections of Irp2−/− (6A-3) brains. Images (10–66) are from rostral to caudal. High resolution files are available from the corresponding author. (ZIP) [file pone.0098072.s013.zip › PerlsIronStain_IRP2KO/6A3-30.jpg]

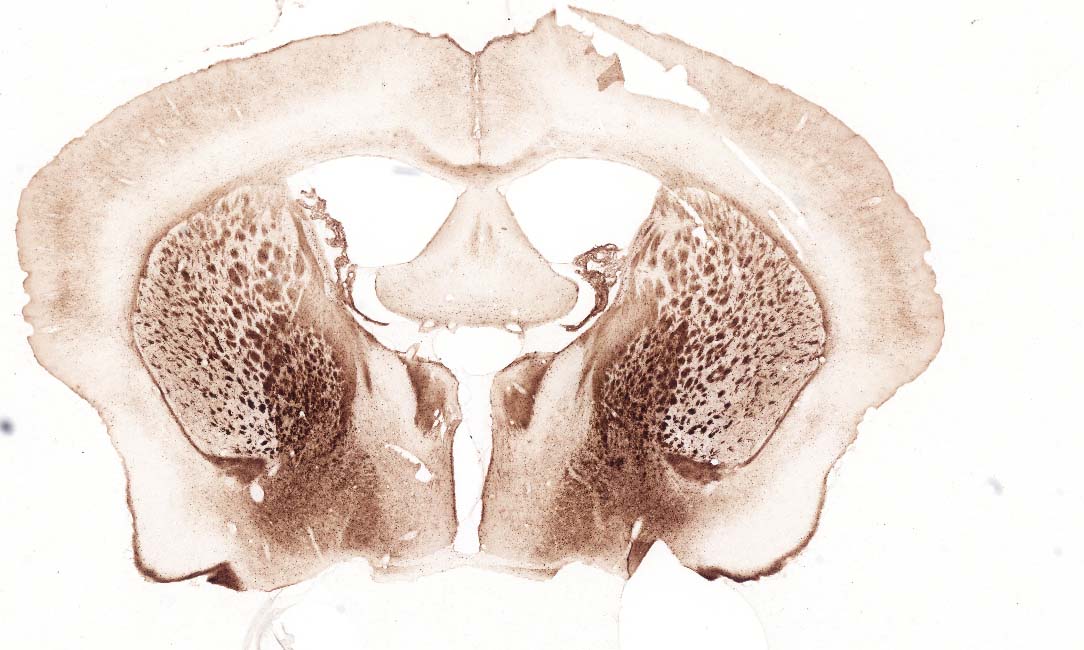

Supplement: File S2 — DAB-enhanced Perls' iron staining of coronal sections of Irp2−/− (6A-3) brains. Images (10–66) are from rostral to caudal. High resolution files are available from the corresponding author. (ZIP) [file pone.0098072.s013.zip › PerlsIronStain_IRP2KO/6A3-31.jpg]

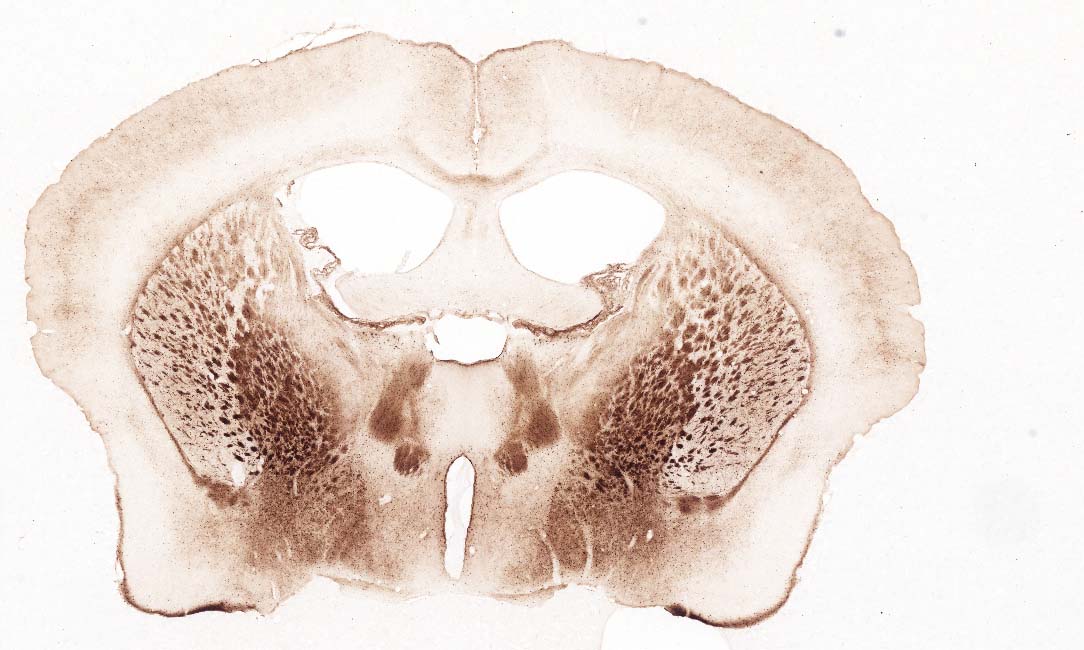

Supplement: File S2 — DAB-enhanced Perls' iron staining of coronal sections of Irp2−/− (6A-3) brains. Images (10–66) are from rostral to caudal. High resolution files are available from the corresponding author. (ZIP) [file pone.0098072.s013.zip › PerlsIronStain_IRP2KO/6A3-32.jpg]

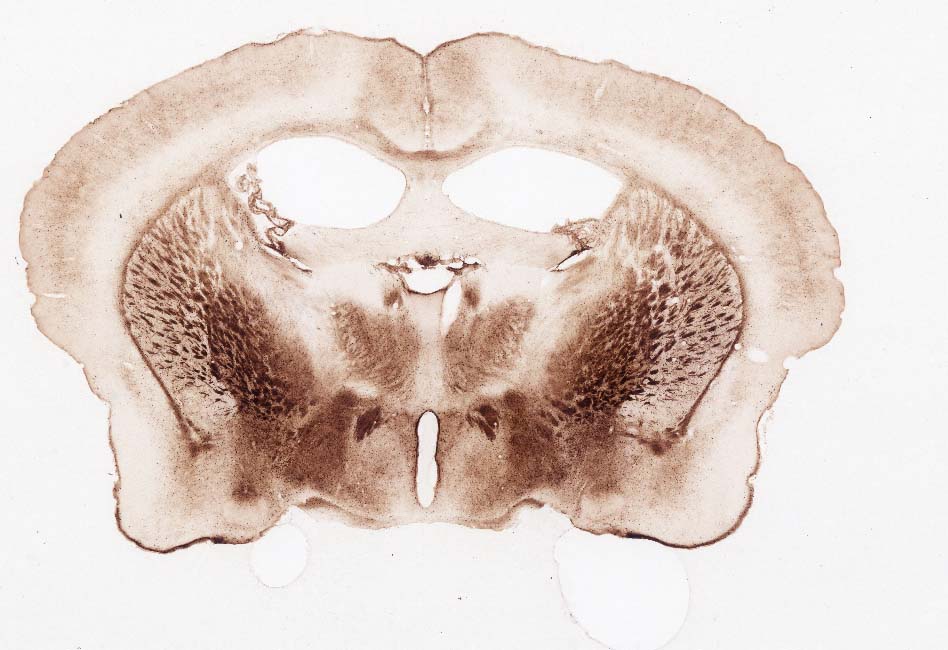

Supplement: File S2 — DAB-enhanced Perls' iron staining of coronal sections of Irp2−/− (6A-3) brains. Images (10–66) are from rostral to caudal. High resolution files are available from the corresponding author. (ZIP) [file pone.0098072.s013.zip › PerlsIronStain_IRP2KO/6A3-33.jpg]

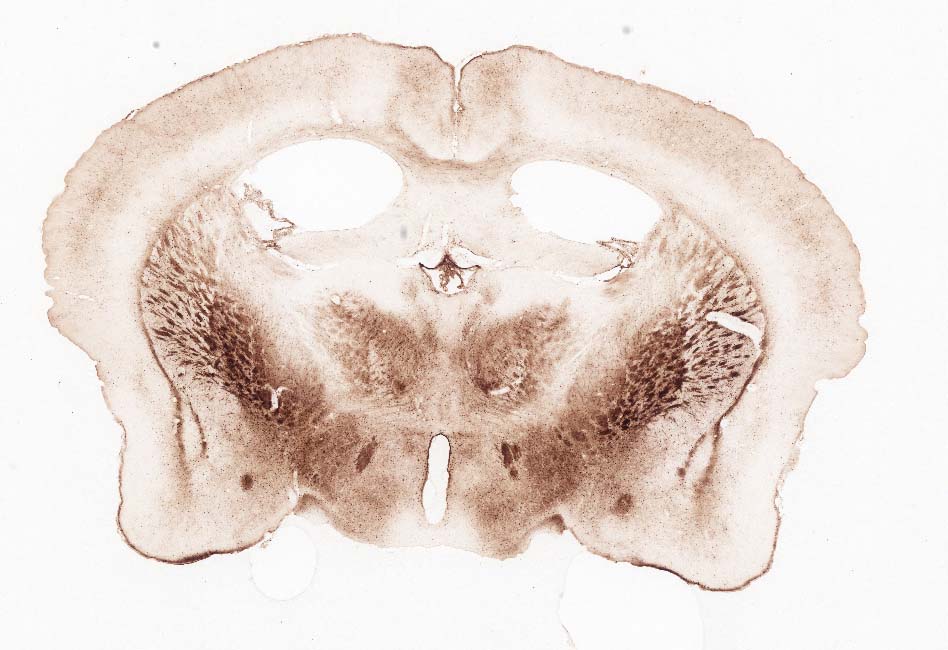

Supplement: File S2 — DAB-enhanced Perls' iron staining of coronal sections of Irp2−/− (6A-3) brains. Images (10–66) are from rostral to caudal. High resolution files are available from the corresponding author. (ZIP) [file pone.0098072.s013.zip › PerlsIronStain_IRP2KO/6A3-34.jpg]

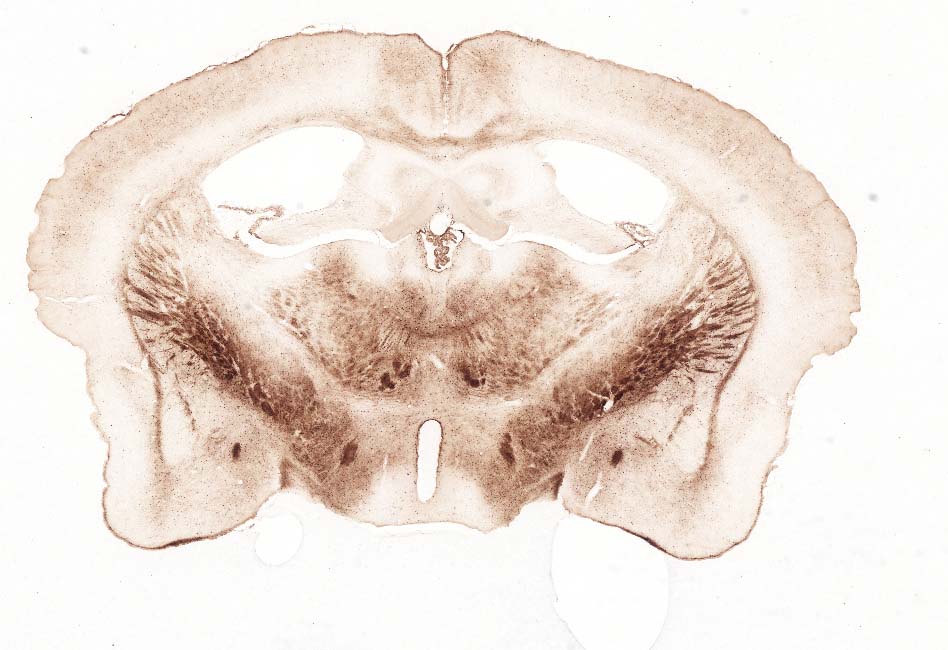

Supplement: File S2 — DAB-enhanced Perls' iron staining of coronal sections of Irp2−/− (6A-3) brains. Images (10–66) are from rostral to caudal. High resolution files are available from the corresponding author. (ZIP) [file pone.0098072.s013.zip › PerlsIronStain_IRP2KO/6A3-35.jpg]

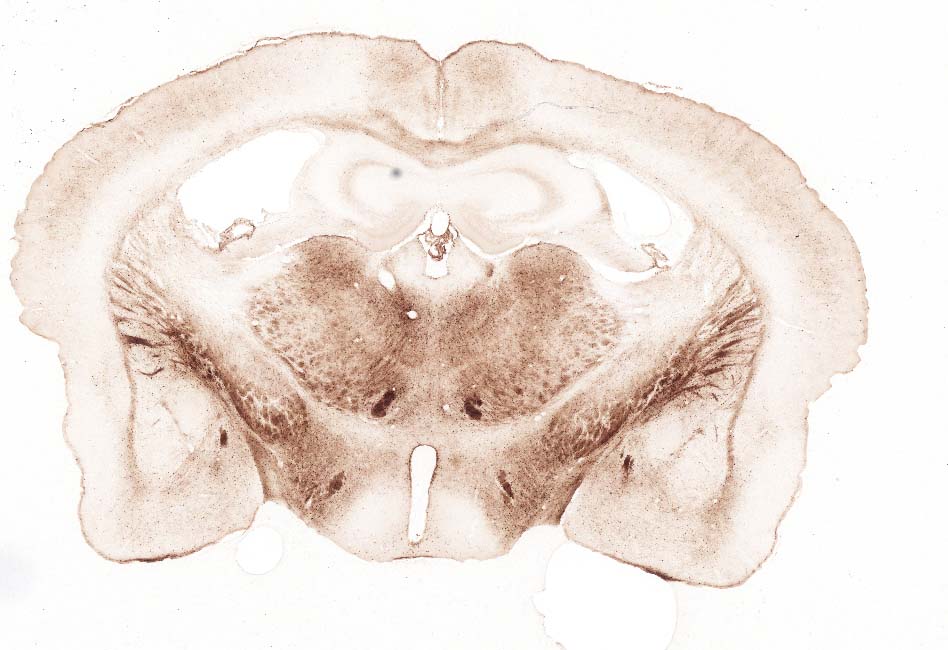

Supplement: File S2 — DAB-enhanced Perls' iron staining of coronal sections of Irp2−/− (6A-3) brains. Images (10–66) are from rostral to caudal. High resolution files are available from the corresponding author. (ZIP) [file pone.0098072.s013.zip › PerlsIronStain_IRP2KO/6A3-36.jpg]

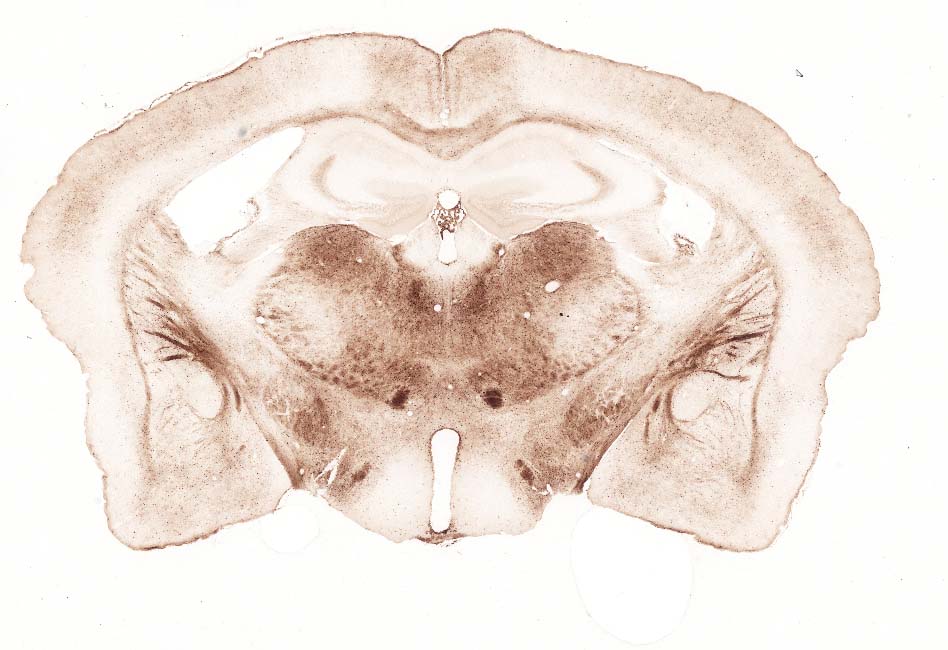

Supplement: File S2 — DAB-enhanced Perls' iron staining of coronal sections of Irp2−/− (6A-3) brains. Images (10–66) are from rostral to caudal. High resolution files are available from the corresponding author. (ZIP) [file pone.0098072.s013.zip › PerlsIronStain_IRP2KO/6A3-37.jpg]

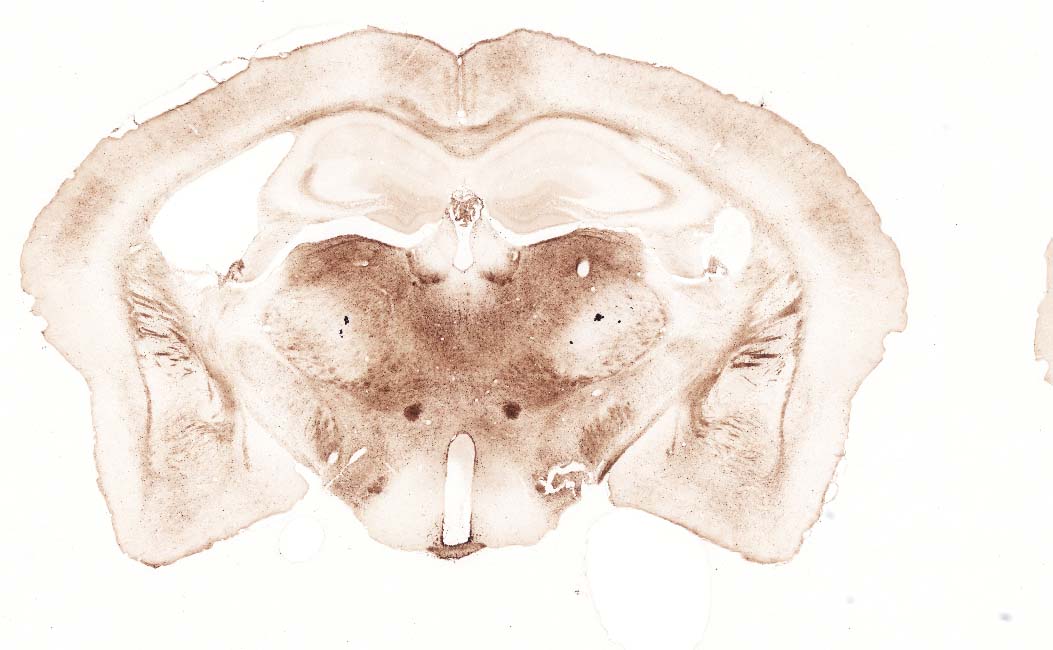

Supplement: File S2 — DAB-enhanced Perls' iron staining of coronal sections of Irp2−/− (6A-3) brains. Images (10–66) are from rostral to caudal. High resolution files are available from the corresponding author. (ZIP) [file pone.0098072.s013.zip › PerlsIronStain_IRP2KO/6A3-38.jpg]

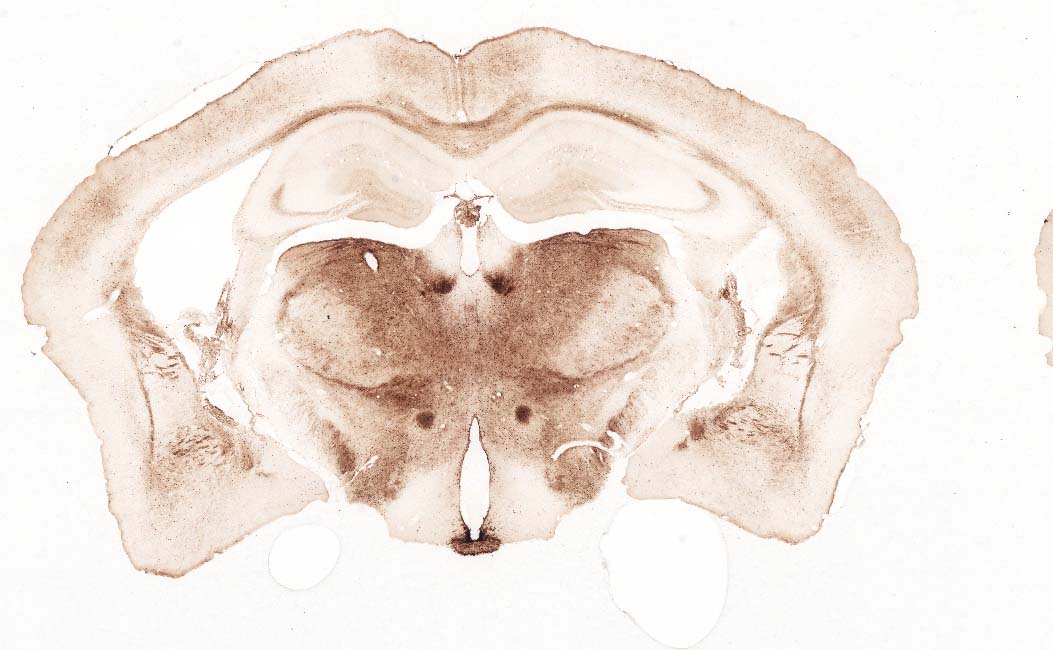

Supplement: File S2 — DAB-enhanced Perls' iron staining of coronal sections of Irp2−/− (6A-3) brains. Images (10–66) are from rostral to caudal. High resolution files are available from the corresponding author. (ZIP) [file pone.0098072.s013.zip › PerlsIronStain_IRP2KO/6A3-39.jpg]

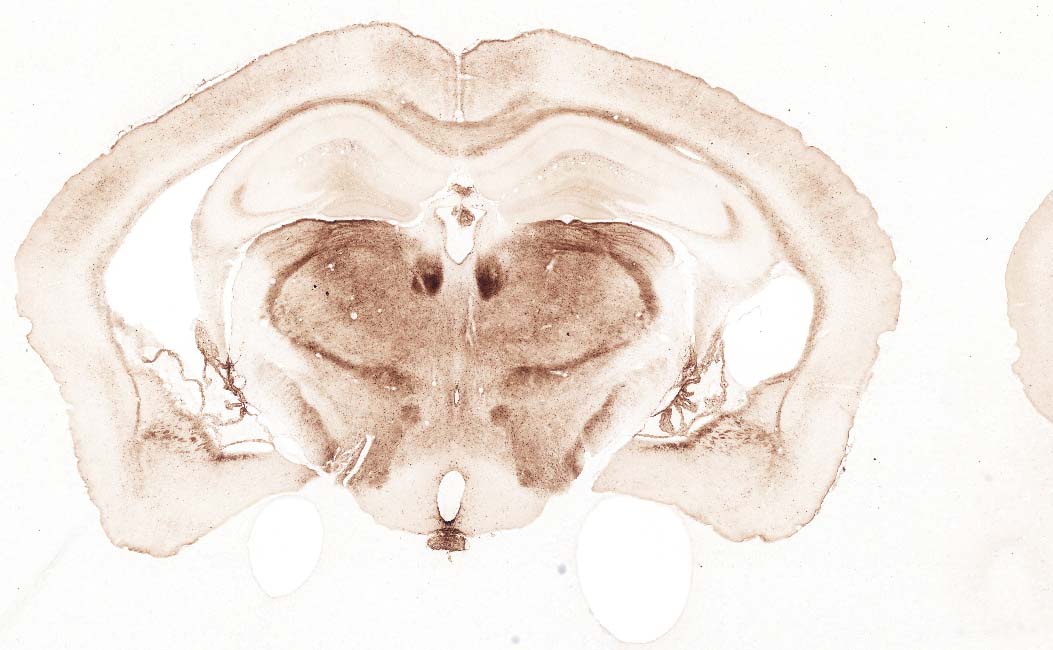

Supplement: File S2 — DAB-enhanced Perls' iron staining of coronal sections of Irp2−/− (6A-3) brains. Images (10–66) are from rostral to caudal. High resolution files are available from the corresponding author. (ZIP) [file pone.0098072.s013.zip › PerlsIronStain_IRP2KO/6A3-40.jpg]

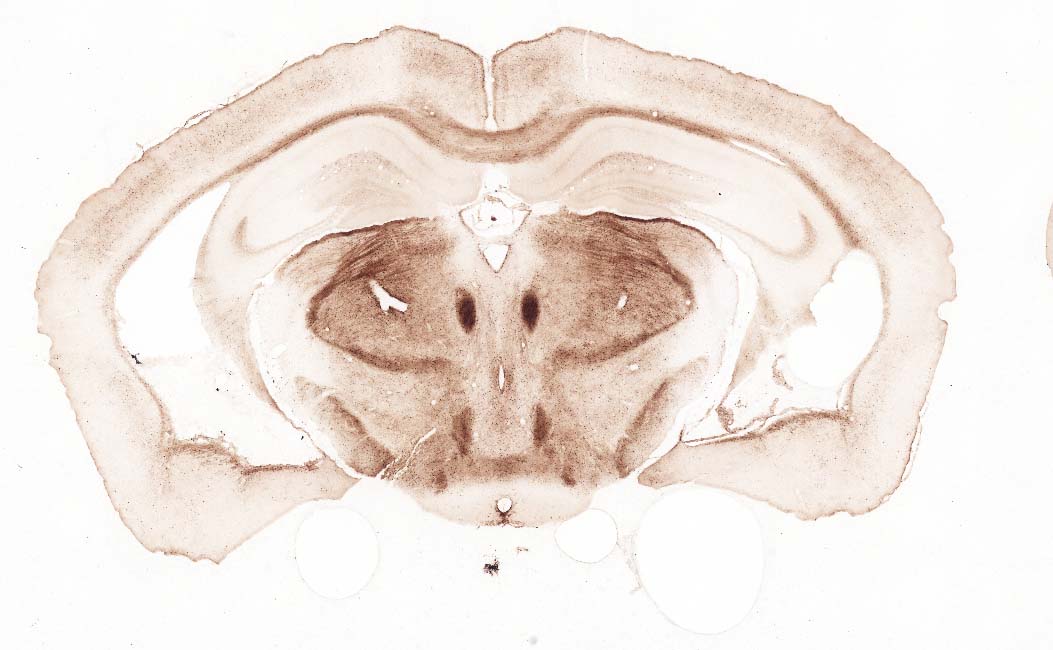

Supplement: File S2 — DAB-enhanced Perls' iron staining of coronal sections of Irp2−/− (6A-3) brains. Images (10–66) are from rostral to caudal. High resolution files are available from the corresponding author. (ZIP) [file pone.0098072.s013.zip › PerlsIronStain_IRP2KO/6A3-41.jpg]

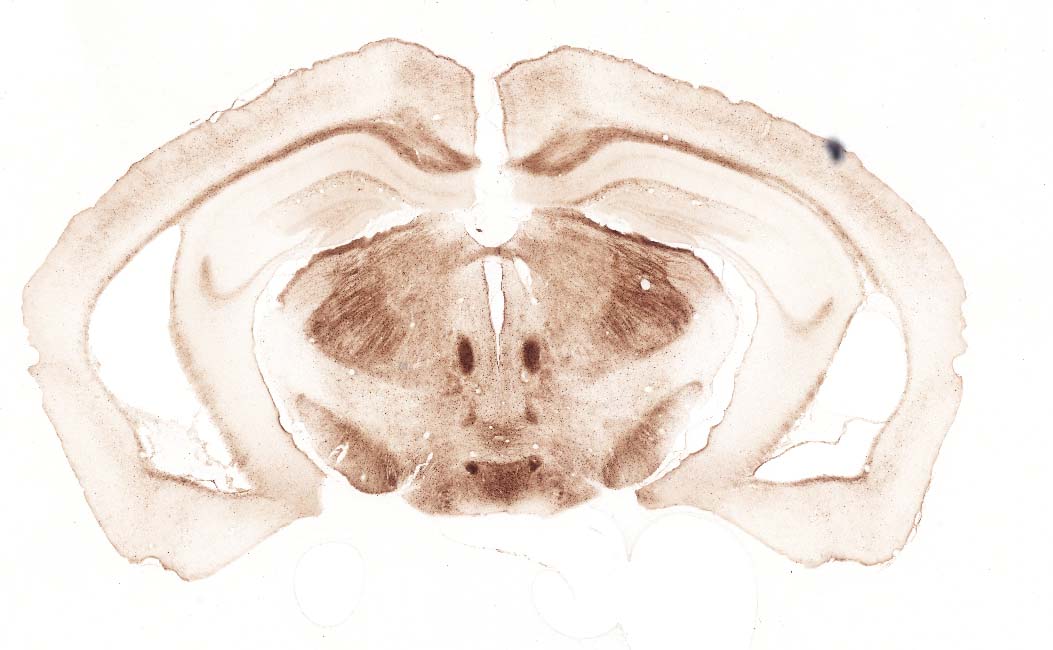

Supplement: File S2 — DAB-enhanced Perls' iron staining of coronal sections of Irp2−/− (6A-3) brains. Images (10–66) are from rostral to caudal. High resolution files are available from the corresponding author. (ZIP) [file pone.0098072.s013.zip › PerlsIronStain_IRP2KO/6A3-42.jpg]

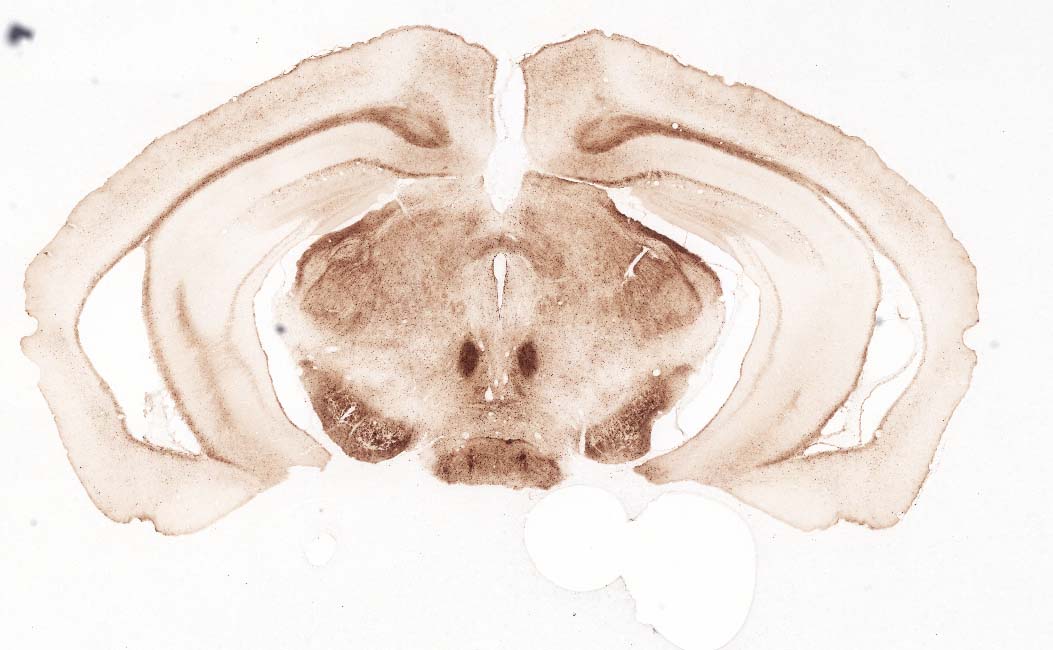

Supplement: File S2 — DAB-enhanced Perls' iron staining of coronal sections of Irp2−/− (6A-3) brains. Images (10–66) are from rostral to caudal. High resolution files are available from the corresponding author. (ZIP) [file pone.0098072.s013.zip › PerlsIronStain_IRP2KO/6A3-43.jpg]

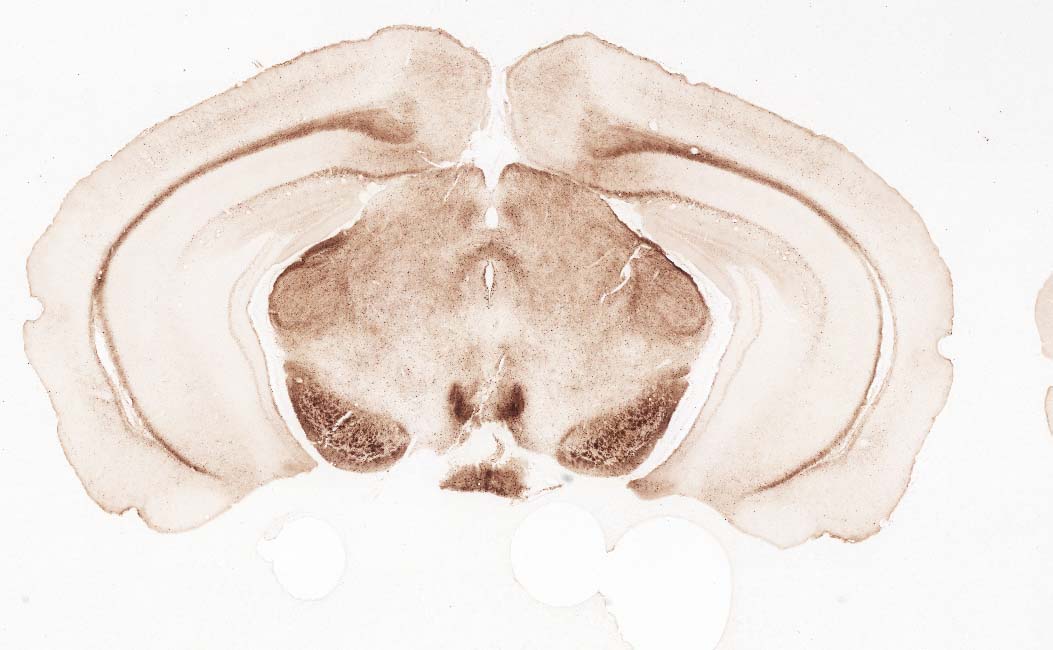

Supplement: File S2 — DAB-enhanced Perls' iron staining of coronal sections of Irp2−/− (6A-3) brains. Images (10–66) are from rostral to caudal. High resolution files are available from the corresponding author. (ZIP) [file pone.0098072.s013.zip › PerlsIronStain_IRP2KO/6A3-44.jpg]

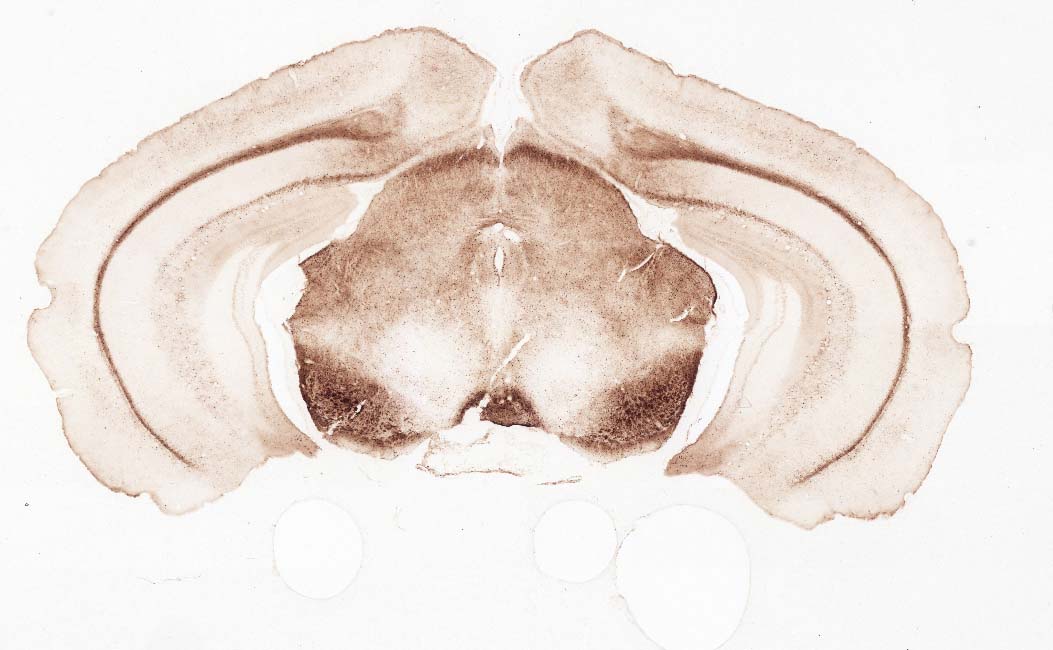

Supplement: File S2 — DAB-enhanced Perls' iron staining of coronal sections of Irp2−/− (6A-3) brains. Images (10–66) are from rostral to caudal. High resolution files are available from the corresponding author. (ZIP) [file pone.0098072.s013.zip › PerlsIronStain_IRP2KO/6A3-45.jpg]

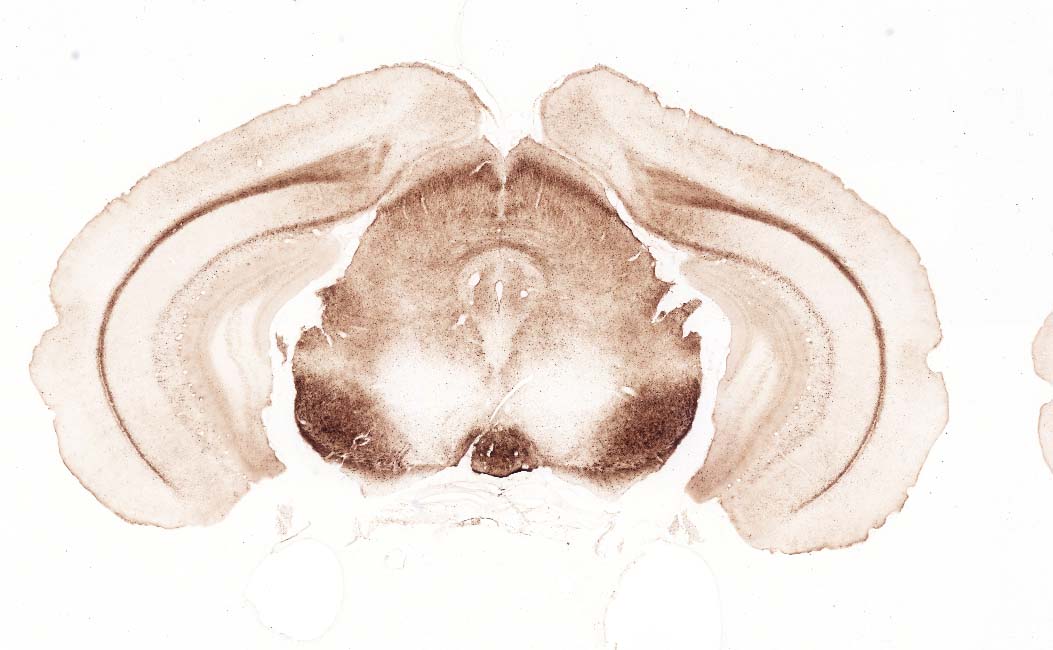

Supplement: File S2 — DAB-enhanced Perls' iron staining of coronal sections of Irp2−/− (6A-3) brains. Images (10–66) are from rostral to caudal. High resolution files are available from the corresponding author. (ZIP) [file pone.0098072.s013.zip › PerlsIronStain_IRP2KO/6A3-46.jpg]

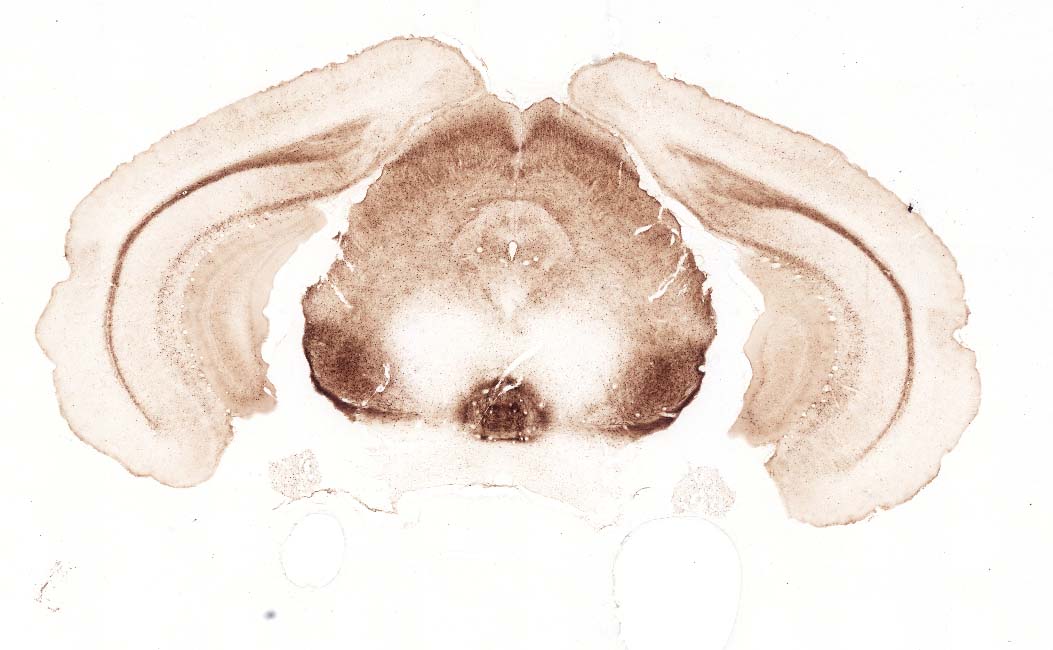

Supplement: File S2 — DAB-enhanced Perls' iron staining of coronal sections of Irp2−/− (6A-3) brains. Images (10–66) are from rostral to caudal. High resolution files are available from the corresponding author. (ZIP) [file pone.0098072.s013.zip › PerlsIronStain_IRP2KO/6A3-47.jpg]
