# Supplementary material for: Abnormal Brain Iron Metabolism in Irp2 Deficient Mice Is Associated with Mild Neurological and Behavioral Impairments
Source: PLoS One. 2014 Jun 4;9(6):e98072. doi: 10.1371/journal.pone.0098072 (PMC4045679; doi:10.1371/journal.pone.0098072)
Supplement: Table S1 — Hematological parameters of aged male WT and Irp2−/− mice. (DOCX) [file pone.0098072.s006.docx]

*Table S1. Hematological parameters of aged male WT and Irp2^-/-^ mice*

|  | ***WT***  (n= 9) | ***Irp2^-/-^***  (n=10) |
| --- | --- | --- |
| WBC (10^3^/ul) | 7.8 ± 0.7 | 7.3 ± 0.4 |
| RBC (10^6^/ul) | 9.6 ± 0.2 | 9.5 ± 0.2 |
| PLT (10^3^/ul) | 1311 ± 36 | 1072 ± 32** |
| Hemoglobin (g/dl) | 14.3 ± 0.2 | 12.3 ± 0.2** |
| Hematocrit (%) | 44.2 ± 0.6 | 38± 0.6** |
| MCV(fl) | 46.3 ± 0.4 | 40.9 ± 0.2** |
| MCH (pg) | 14.9 ± 0.2 | 12.9 ± 0.1** |
| MCHC (g/dl) | 32.4 ± 0.2 | 31.5 ± 0.1** |
| RDW (% of MCV) | 15.4 ± 0.2 | 16 ± 0.1* |
| MPV (fl) | 5.3 ± 0.1 | 5.0 ± 0.1* |

Statistical analysis was performed by paired Student’s *t*-test (**p* < 0.05, ***p* < 0.01, mean ± SEM). MCH, mean corpuscular hemoglobin; MCV, mean cell volume; MCHC, mean corpuscular hemoglobin concentration; RDW, red blood cell distribution width; MPV, mean platelet volume. Ages of mice: WT, 64-75 weeks; *Irp2^-/-^*, 49-71 weeks.
