# Supplementary material for: Abnormal Brain Iron Metabolism in Irp2 Deficient Mice Is Associated with Mild Neurological and Behavioral Impairments
Source: PLoS One. 2014 Jun 4;9(6):e98072. doi: 10.1371/journal.pone.0098072 (PMC4045679; doi:10.1371/journal.pone.0098072)
Supplement: Table S2 — Serum iron parameters of aged male WT and Irp2−/− mice. (DOCX) [file pone.0098072.s007.docx]

*Table S2. Serum iron parameters of aged male WT and Irp2^-/-^ mice*

|  | ***WT***  (n=9) | ***Irp2^-/-^***  (n=10) |
| --- | --- | --- |
| Ferritin [ng/ml] | 58.1 ± 12.5 | 218.6 ± 7.3 *** |
| Transferrin [mg/dl] | 167.5 ± 5.8 | 159.1 ± 2.64 |
| UIBC ug/dl] | 286.8 ± 13.59 | 257.5 ± 5.52 |
| Transferrin sat. [%] | 32.7 ± 0.96 | 35 ± 1.39 |
| Iron [ug/dl] | 139.6 ± 6.79 | 139.4 ± 7.76 |

Statistical analysis was performed using the paired Student’s *t*-test (****p* < 0.001, mean ± SEM). UIBC, unbound iron binding capacity. Transferrin saturation was calculated from iron and UIBC values as percentage of iron level on total iron binding capacity (TIBC = iron+UIBC). Ages mice: WT 64-75 weeks; *Irp2^-/-^*, 49-71 weeks.
