# Supplementary material for: Abnormal Brain Iron Metabolism in Irp2 Deficient Mice Is Associated with Mild Neurological and Behavioral Impairments
Source: PLoS One. 2014 Jun 4;9(6):e98072. doi: 10.1371/journal.pone.0098072 (PMC4045679; doi:10.1371/journal.pone.0098072)
Supplement: Table S3 — Protoporphyrin IX (PPIX) levels of 12-month old male WT and Irp2−/− mice. (DOCX) [file pone.0098072.s008.docx]

*Table S3. Protoporphyrin IX (PPIX) levels of 12-month old male WT and Irp2^-/-^ mice*

|  | ***WT***  (n=7-9) | ***Irp2^-/-^***  (n=8-9) |
| --- | --- | --- |
| Serum PPIX (uM) | 0.009 ± 0.001 | 0.794 ± 0.235 * |
| Liver PPIX (pmol/mg) | 0.0048 ± 0.0007 | 0.026 ± 0.004 ** |
| Bile duct PPIX (pmol/mg) | 0.016 ± 0.0069 | 2.31 ± 0.202 *** |

Statistical analysis performed using the paired Student’s t-test (**p* < 0.05, ***p* < 0.01, ****p* < 0.001, mean ± SEM).
