# Supplementary material for: Abnormal Brain Iron Metabolism in Irp2 Deficient Mice Is Associated with Mild Neurological and Behavioral Impairments
Source: PLoS One. 2014 Jun 4;9(6):e98072. doi: 10.1371/journal.pone.0098072 (PMC4045679; doi:10.1371/journal.pone.0098072)
Supplement: Table S4 — Results of behavioral observation in the modified-Hole Board test. (DOCX) [file pone.0098072.s009.docx]

*Table S4. Results of behavioral observation in the modified-Hole Board test*

| **Parameter** | | ***WT***  (n=9) | ***Irp2^-/-^***  (n=10) |  | |
| --- | --- | --- | --- | --- | --- |
|  |  |  |  | *p*-value |  |
| Line crossing [frequency] | | 73.3 ± 7.8 | 48.9 ± 6.5 | <0.05 |  |
| Line crossing [latency] | | 5.1 ± 1.4 | 6.5 ± 1.4 | n.s. |  |
| Rearings in box [frequency] | | 11.7 ± 1.1 | 2.9 ± 1.2 | <0.0001 |  |
| Rearings in box [latency] | | 96 ± 14.4 | 225.5 ± 30.5 | <0.01 |  |
| Hole exploration [frequency] | | 29.6 ± 3.7 | 29.1 ± 4 | n.s. |  |
| Hole exploration [latency] | | 18.7 ± 7.3 | 12.6 ± 4.4 | n.s. |  |
| Board entry [frequency] | | 6.7 ± 1.6 | 4.6 ± 1 | n.s. |  |
| Board entry [latency] | | 40.6 ± 14.6 | 63.3 ± 27.6 | n.s. |  |
| Board entry [total duration %] | | 12.6 ± 2.3 | 17 ± 3.8 | n.s. |  |
| Rearing on board [frequency] | | 0 ± 0 | 0.1 ± 0.1 | n.s. |  |
| Rearing on board [latency] | | 300 ± 0 | 279.8 ± 20.3 | n.s. |  |
| Risk assessment [frequency] | | 0 ± 0 | 0 ± 0 | n.s. |  |
| Risk assessment [latency] | | 300 ± 0 | 300 ± 0 | n.s. |  |
| Grooming [frequency] | 2.2 ± 0.5 | 2 ± 0.5 | n.s. |  | |
| Grooming [latency] | 132.7 ± 23.4 | 166.1 ± 24.9 | n.s. |  |  |
| Grooming [total duration %] | 2.82 ± 0.5 | 4.1 ± 0.9 | n.s. |  |  |
| Defecation [frequency] | 0.3 ± 0.2 | 1.2 ± 0.6 | n.s. |  |  |
| Defecation [latency] | 229.6 ± 38.2 | 242.7 ± 25.5 | n.s. |  |  |
| Unfamiliar object exploration [frequency] | 4.8 ± 0.7 | 3.8 ± 0.9 | n.s. |  |  |
| Familiar object exploration [frequency] | 5.3 ± 0.3 | 3.9 ± 0.9 | n.s. |  |  |
| Unfamiliar object exploration [latency] | 73.2 ± 13.7 | 103.8 ± 19.3 | n.s. |  |  |
| Familiar object exploration [latency] | 62.1 ± 9.1 | 104.4 ± 28.3 | n.s. |  |  |
| Unfamiliar object exploration [total duration %] | 0.6 ± 0.1 | 1.5 ± 0.5 | n.s. |  |  |
| Familiar object exploration [total duration %] | 0.6 ± 0.1 | 0.8 ± 0.2 | n.s. |  |  |
| Object Index | 0.48 ± 0.04 | 0.59 ± 0.07 | n.s. |  |  |

Statistical analysis performed using the paired Student’s t-test (two-tailed). Data are presented as mean ± SEM. n.s., not significant. Ages of male mice: WT, 57-63 weeks; *Irp2^-/-^*, 38-45 weeks.
