# Supplementary material for: Abnormal Brain Iron Metabolism in Irp2 Deficient Mice Is Associated with Mild Neurological and Behavioral Impairments
Source: PLoS One. 2014 Jun 4;9(6):e98072. doi: 10.1371/journal.pone.0098072 (PMC4045679; doi:10.1371/journal.pone.0098072)
Supplement: Table S5 — Video-tracking results regarding locomotor behavior. (DOCX) [file pone.0098072.s010.docx]

| *Table S5. Video-tracking results regarding locomotor behavior* | | |  |  |  |  |  |  |
| --- | --- | --- | --- | --- | --- | --- | --- | --- |
| **Parameter** | ***WT***  (n=9) | ***Irp2^-/-^***  (n=10) | |  | | | |  |
|  |  |  |  | *p*-value | |  | |  |
| Total distance moved [cm] | 2079 ± 163.99 | 1419.54 ± 168.54 | | | <0.05 | |  | |
| Mean velocity [cm/sec] | 13.25 ± 0.63 | 10.79 ± 0.76 | | | <0.05 | |  | |
| Maximum velocity [cm/sec] | 35.24 ± 1.24 | 33.11 ± 3.3 | | | n.s. | |  | |
| Turns [frequency] | 1395.78 ± 84.71 | 997.5 ± 106.88 | | | <0.05 | |  | |
| Mean turn angle degrees] | 28.68 ± 1.32 | 27.13 ± 1.01 | | | n.s. | |  | |
| Angular velocity [degrees/sec] | 170.03 ± 2.74 | 140.73 ± 6.05 | | | <0.001 | |  | |
| Absolute meander [degrees/sec] | 22.24 ± 1.2 | 21.93 ± 0.98 | | | n.s. | |  | |
| Board entry [maximum duration/sec] | 21.37 ± 8.35 | 30.85 ± 9.6 | | | n.s. | |  | |
| Mean distance to wall [cm] | 7.8 ± 0.28 | 8.75 ± 0.52 | | | n.s. | |  | |
| Mean distance to board [cm] | 8.31 ± 0.12 | 7.7 ± 0.28 | | | *p*=0.07 | |  | |

Statistical analysis performed using the paired Student’s t-test. Data are presented as mean ± SEM. n.s., not significant. Ages of male mice: WT, 57-63 weeks; *Irp2^-/-^*, 38-45 weeks.
