# Supplementary material for: Abnormal Brain Iron Metabolism in Irp2 Deficient Mice Is Associated with Mild Neurological and Behavioral Impairments
Source: PLoS One. 2014 Jun 4;9(6):e98072. doi: 10.1371/journal.pone.0098072 (PMC4045679; doi:10.1371/journal.pone.0098072)
Supplement: Table S6 — List of primers and TaqMan assays used for genotyping and qRT-PCR. (DOCX) [file pone.0098072.s011.docx]

*Table S6.* *List of primers and TaqMan assays used for genotyping or qRT-PCR analysis*

| **Experiment** | **Primer Name or**  **Gene** | **Primer Sequence (5’ - 3’) or TaqMan Assay Number** | |
| --- | --- | --- | --- |
| Genotyping | Irp2_int.for | | gagactaactgggacaatagggttg |
|  | Irp2_fus.rev | | caagatcagcagggcagacaggg |
|  | Irp2_IE.rev | | gctgtcactcacgtgaaatcttgaag |
| qRT-PCR | Irp2 (exon 1-2) | | Mm01179594.m1 |
|  | Irp2 (exon 3-4) | | Mm01179596.m1 |
|  | Irp1 | | Hs00924500.m1 |
|  | Actb | | Mm00607939_s1 |
|  |  | |  |

Genotyping primers are listed in the 5’ to 3’ direction. For qRT-PCR analysis, Taqman assay numbers are indicated with Mm or Hs designating mouse or human, respectively.
