# Supplementary figures and images for: Abnormal Brain Iron Metabolism in Irp2 Deficient Mice Is Associated with Mild Neurological and Behavioral Impairments (part 2 of 2)
Source: PLoS One. 2014 Jun 4;9(6):e98072. doi: 10.1371/journal.pone.0098072 (PMC4045679; doi:10.1371/journal.pone.0098072)

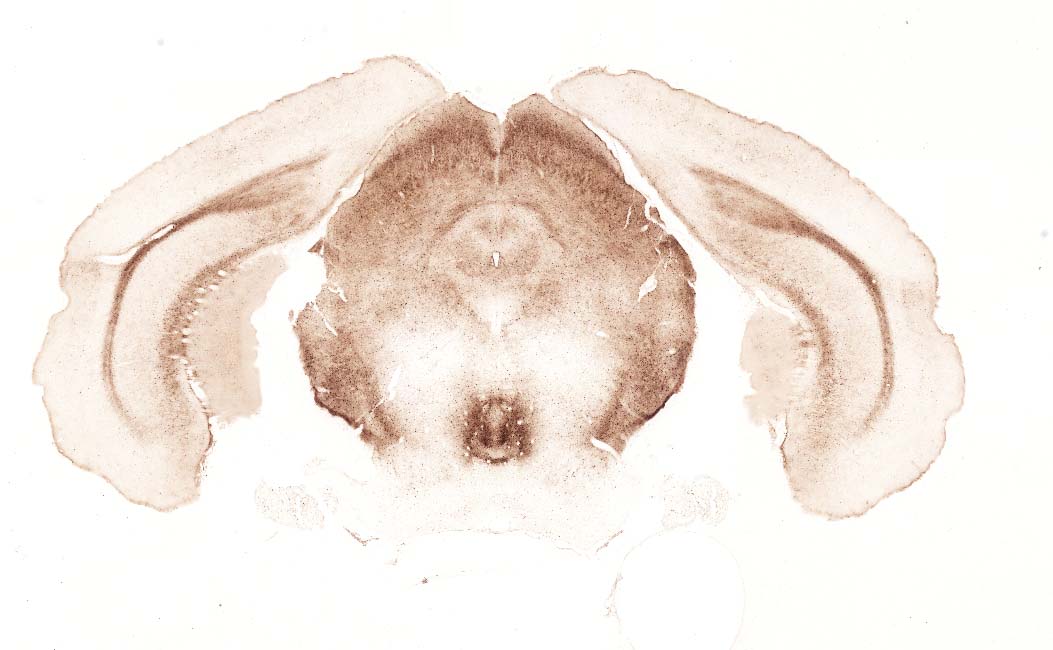

Supplement: File S2 — DAB-enhanced Perls' iron staining of coronal sections of Irp2−/− (6A-3) brains. Images (10–66) are from rostral to caudal. High resolution files are available from the corresponding author. (ZIP) [file pone.0098072.s013.zip › PerlsIronStain_IRP2KO/6A3-48.jpg]

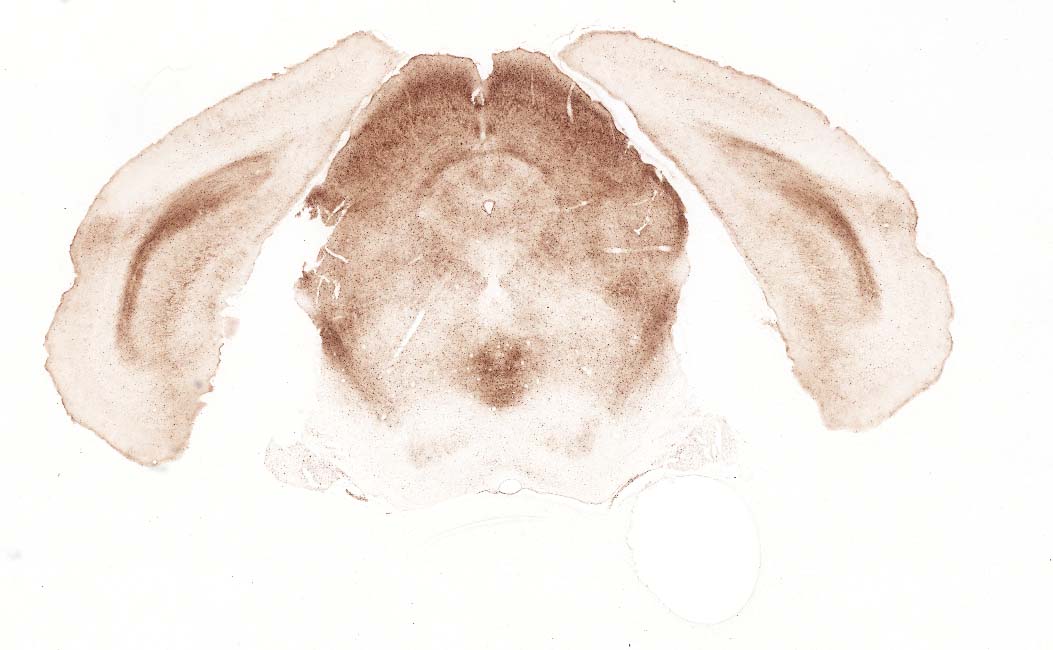

Supplement: File S2 — DAB-enhanced Perls' iron staining of coronal sections of Irp2−/− (6A-3) brains. Images (10–66) are from rostral to caudal. High resolution files are available from the corresponding author. (ZIP) [file pone.0098072.s013.zip › PerlsIronStain_IRP2KO/6A3-49.jpg]

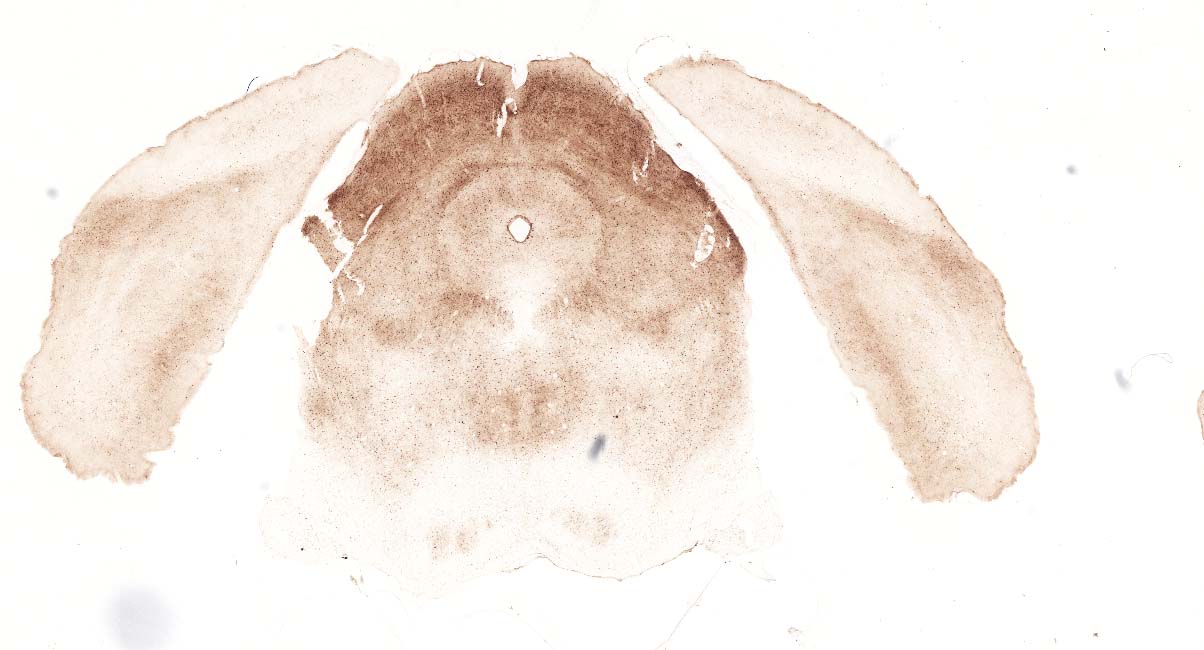

Supplement: File S2 — DAB-enhanced Perls' iron staining of coronal sections of Irp2−/− (6A-3) brains. Images (10–66) are from rostral to caudal. High resolution files are available from the corresponding author. (ZIP) [file pone.0098072.s013.zip › PerlsIronStain_IRP2KO/6A3-50.jpg]

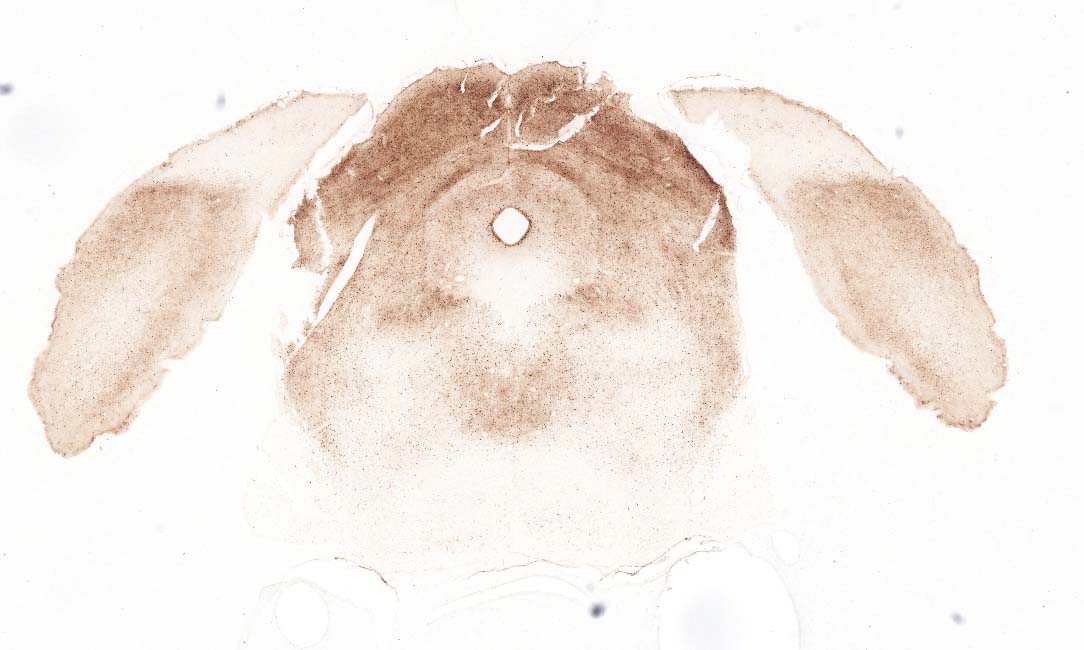

Supplement: File S2 — DAB-enhanced Perls' iron staining of coronal sections of Irp2−/− (6A-3) brains. Images (10–66) are from rostral to caudal. High resolution files are available from the corresponding author. (ZIP) [file pone.0098072.s013.zip › PerlsIronStain_IRP2KO/6A3-51.jpg]

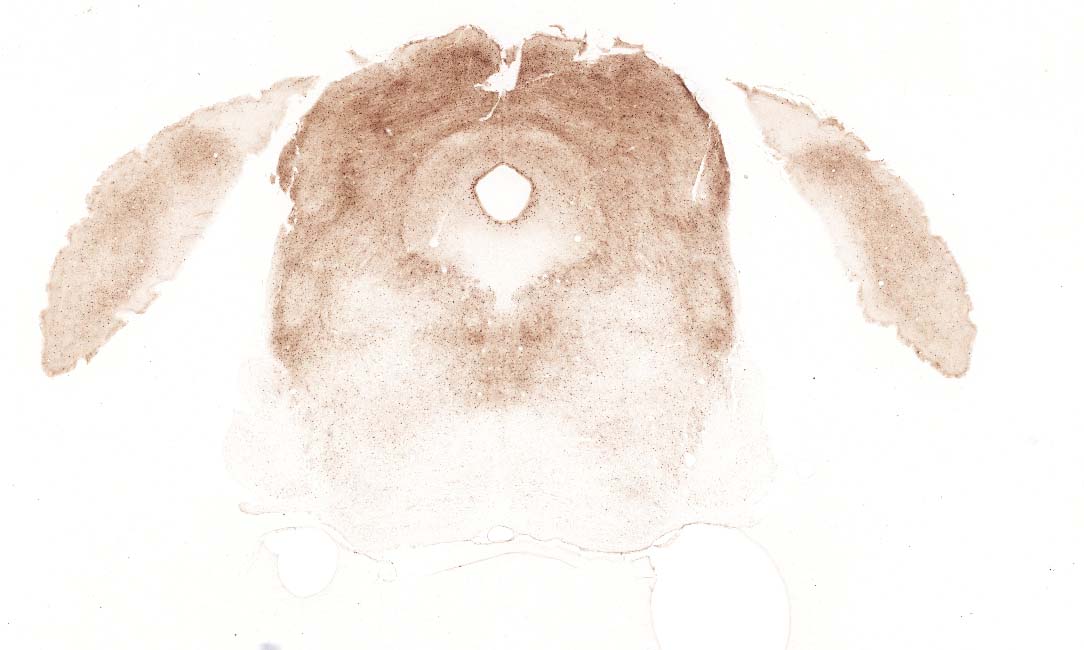

Supplement: File S2 — DAB-enhanced Perls' iron staining of coronal sections of Irp2−/− (6A-3) brains. Images (10–66) are from rostral to caudal. High resolution files are available from the corresponding author. (ZIP) [file pone.0098072.s013.zip › PerlsIronStain_IRP2KO/6A3-52.jpg]

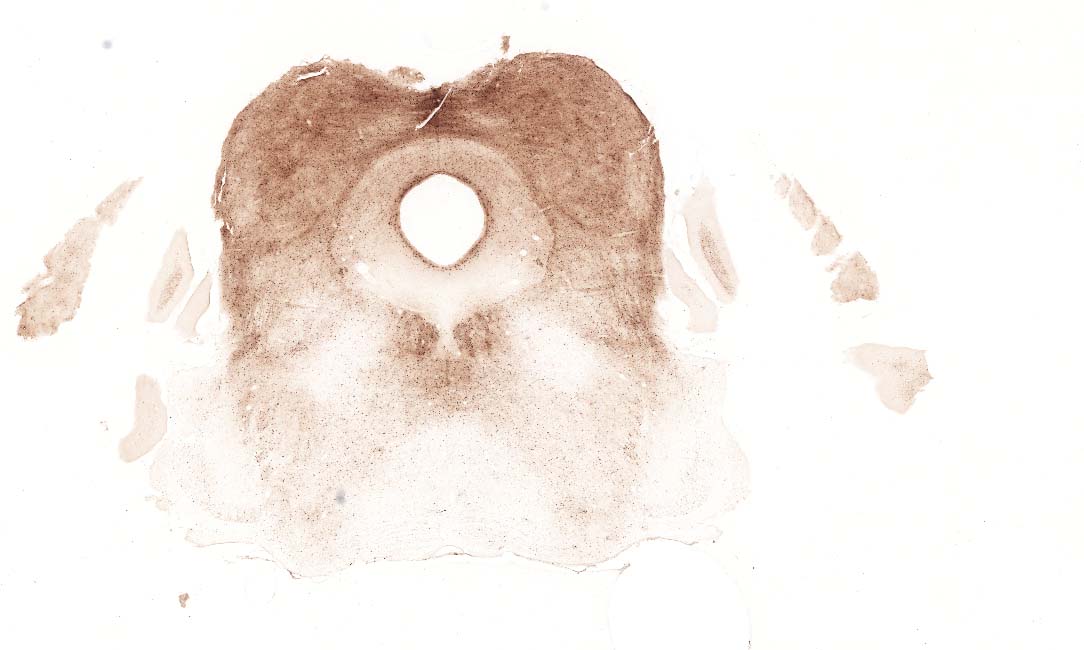

Supplement: File S2 — DAB-enhanced Perls' iron staining of coronal sections of Irp2−/− (6A-3) brains. Images (10–66) are from rostral to caudal. High resolution files are available from the corresponding author. (ZIP) [file pone.0098072.s013.zip › PerlsIronStain_IRP2KO/6A3-53.jpg]

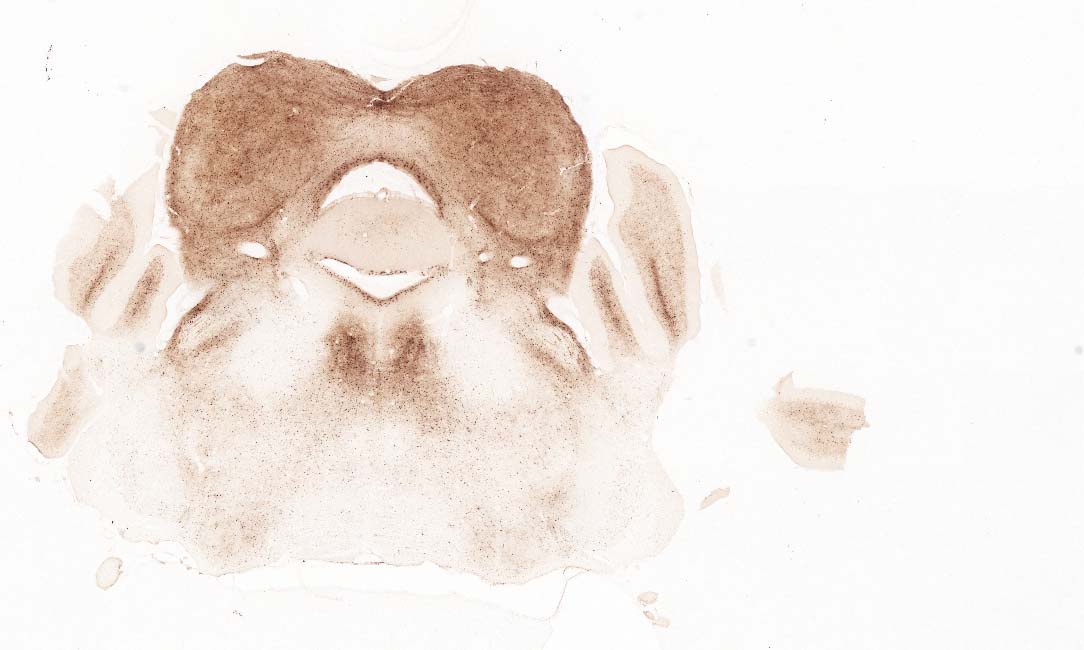

Supplement: File S2 — DAB-enhanced Perls' iron staining of coronal sections of Irp2−/− (6A-3) brains. Images (10–66) are from rostral to caudal. High resolution files are available from the corresponding author. (ZIP) [file pone.0098072.s013.zip › PerlsIronStain_IRP2KO/6A3-54.jpg]

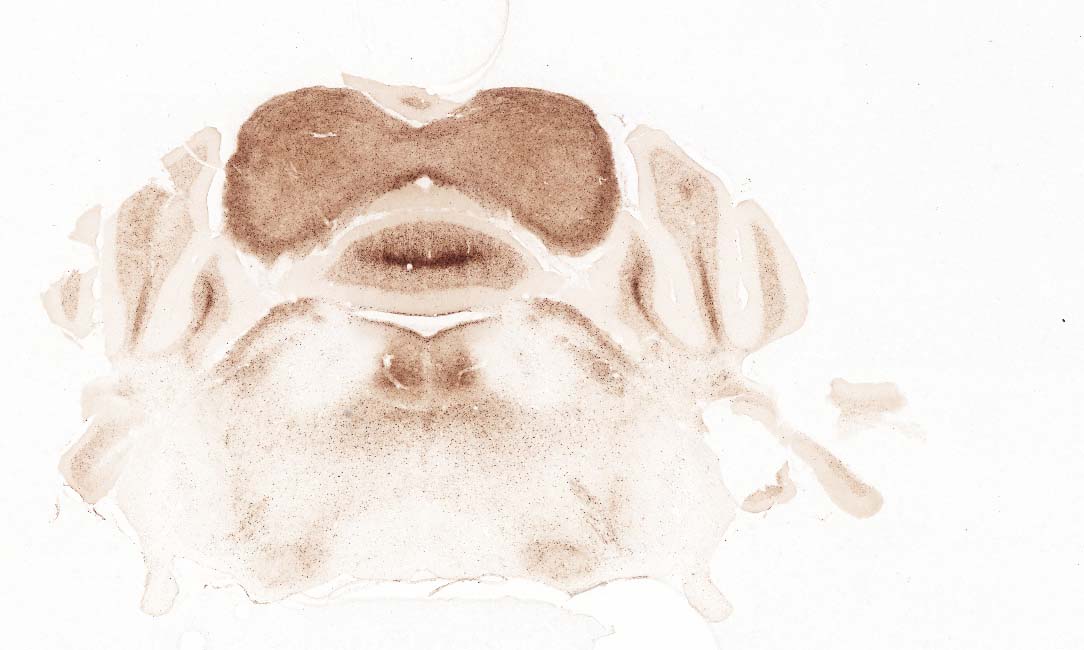

Supplement: File S2 — DAB-enhanced Perls' iron staining of coronal sections of Irp2−/− (6A-3) brains. Images (10–66) are from rostral to caudal. High resolution files are available from the corresponding author. (ZIP) [file pone.0098072.s013.zip › PerlsIronStain_IRP2KO/6A3-55.jpg]

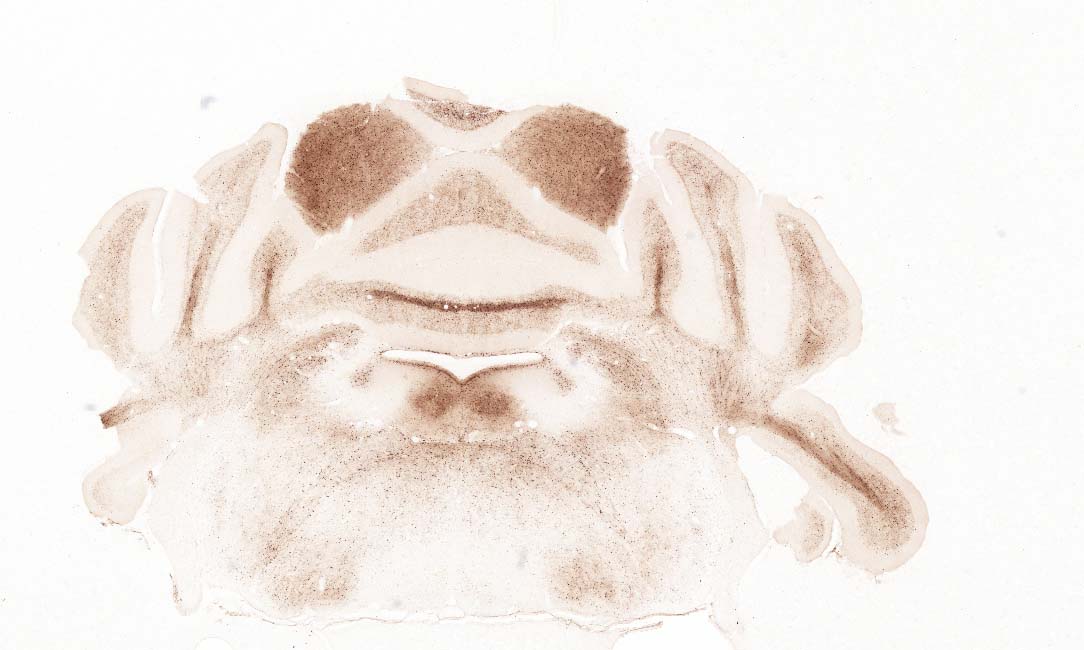

Supplement: File S2 — DAB-enhanced Perls' iron staining of coronal sections of Irp2−/− (6A-3) brains. Images (10–66) are from rostral to caudal. High resolution files are available from the corresponding author. (ZIP) [file pone.0098072.s013.zip › PerlsIronStain_IRP2KO/6A3-56.jpg]

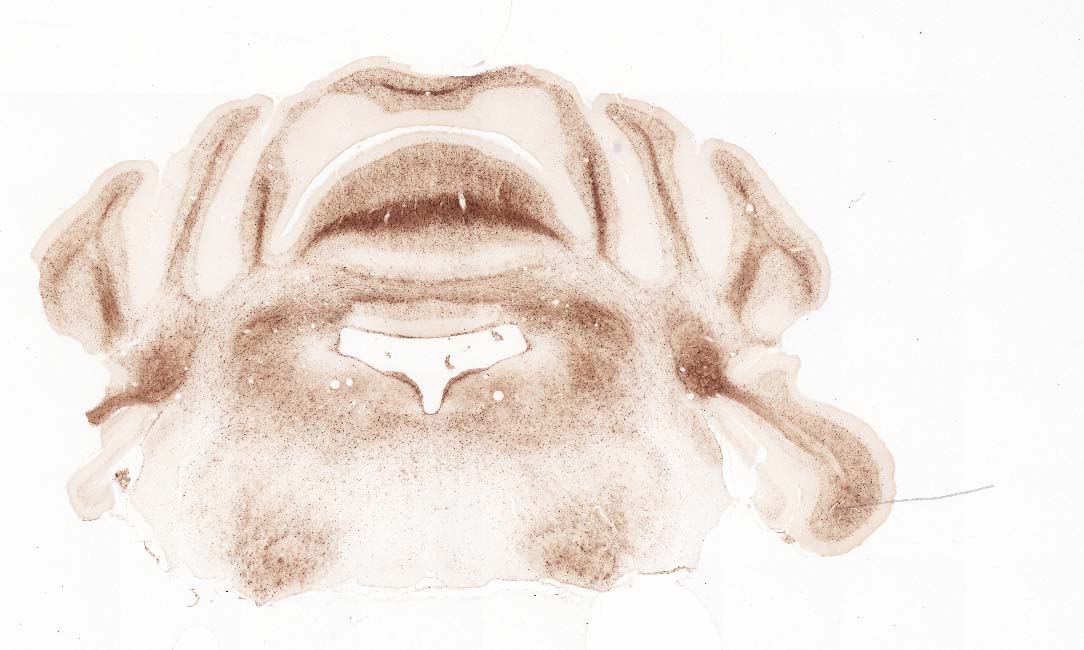

Supplement: File S2 — DAB-enhanced Perls' iron staining of coronal sections of Irp2−/− (6A-3) brains. Images (10–66) are from rostral to caudal. High resolution files are available from the corresponding author. (ZIP) [file pone.0098072.s013.zip › PerlsIronStain_IRP2KO/6A3-57.jpg]

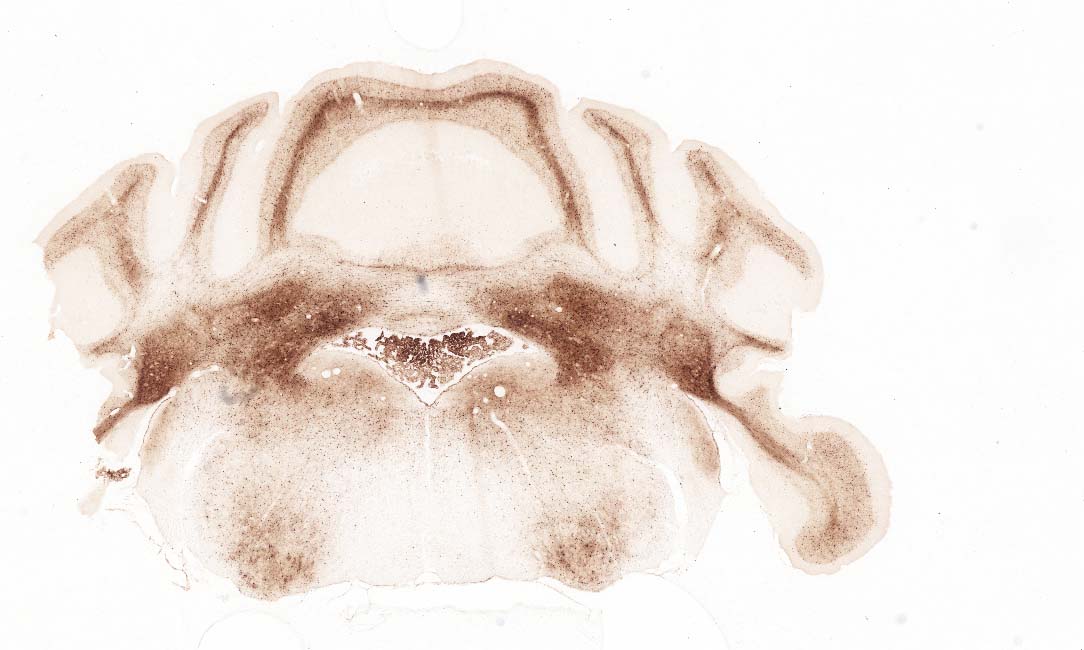

Supplement: File S2 — DAB-enhanced Perls' iron staining of coronal sections of Irp2−/− (6A-3) brains. Images (10–66) are from rostral to caudal. High resolution files are available from the corresponding author. (ZIP) [file pone.0098072.s013.zip › PerlsIronStain_IRP2KO/6A3-58.jpg]

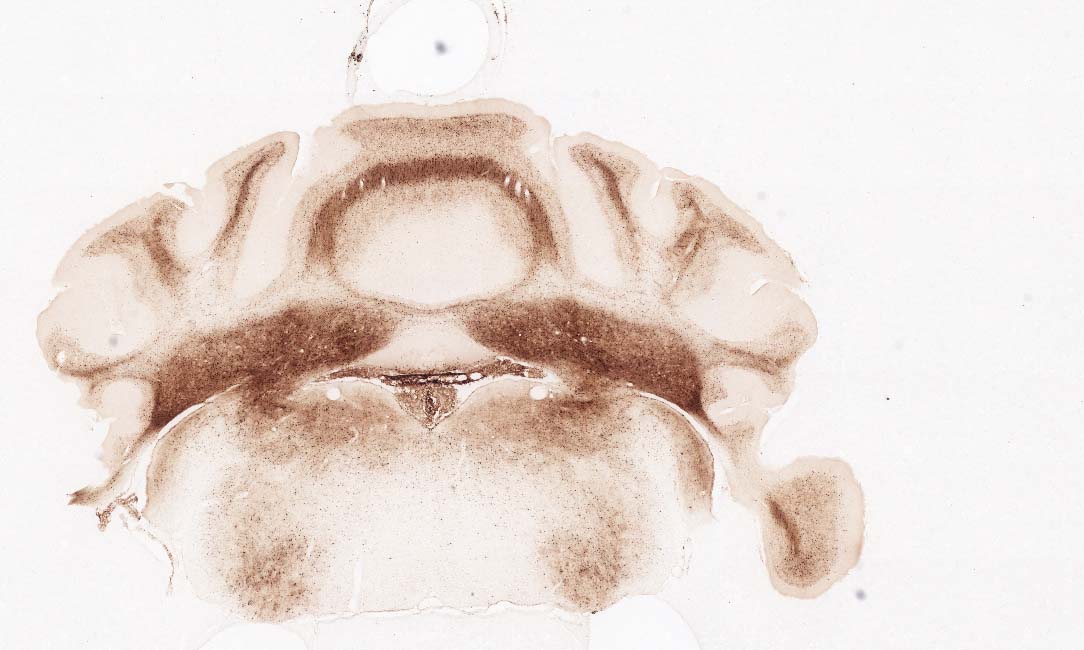

Supplement: File S2 — DAB-enhanced Perls' iron staining of coronal sections of Irp2−/− (6A-3) brains. Images (10–66) are from rostral to caudal. High resolution files are available from the corresponding author. (ZIP) [file pone.0098072.s013.zip › PerlsIronStain_IRP2KO/6A3-59.jpg]

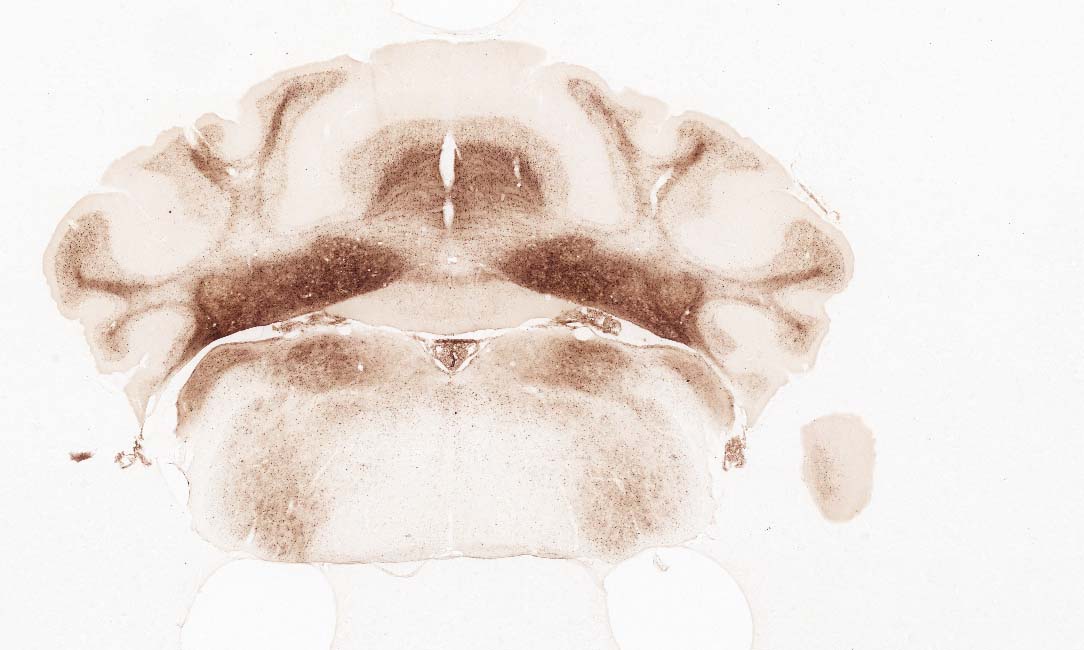

Supplement: File S2 — DAB-enhanced Perls' iron staining of coronal sections of Irp2−/− (6A-3) brains. Images (10–66) are from rostral to caudal. High resolution files are available from the corresponding author. (ZIP) [file pone.0098072.s013.zip › PerlsIronStain_IRP2KO/6A3-60.jpg]

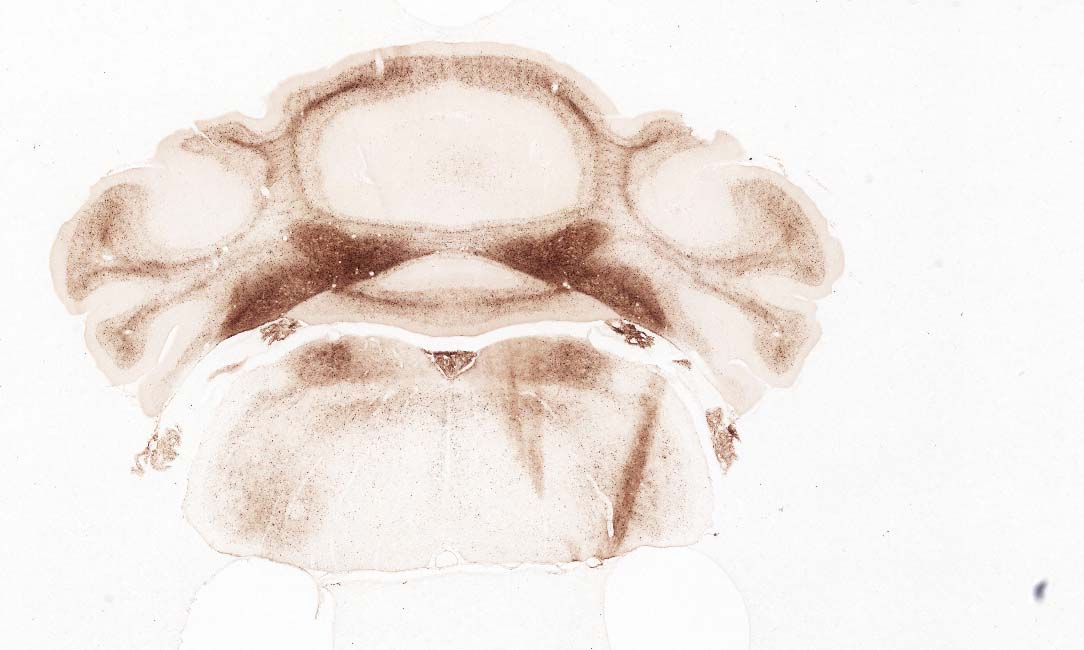

Supplement: File S2 — DAB-enhanced Perls' iron staining of coronal sections of Irp2−/− (6A-3) brains. Images (10–66) are from rostral to caudal. High resolution files are available from the corresponding author. (ZIP) [file pone.0098072.s013.zip › PerlsIronStain_IRP2KO/6A3-61.jpg]

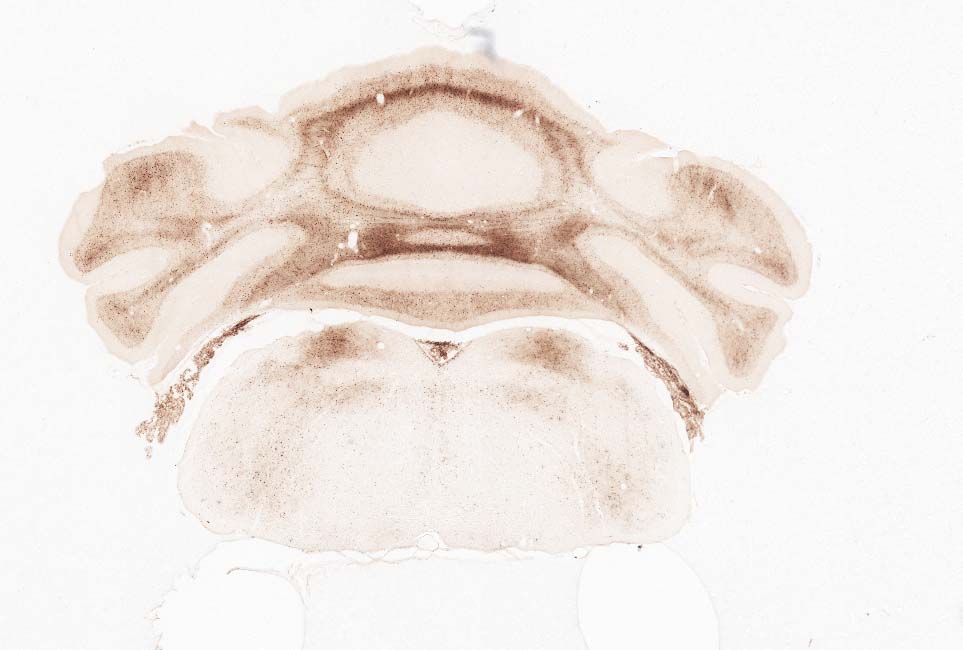

Supplement: File S2 — DAB-enhanced Perls' iron staining of coronal sections of Irp2−/− (6A-3) brains. Images (10–66) are from rostral to caudal. High resolution files are available from the corresponding author. (ZIP) [file pone.0098072.s013.zip › PerlsIronStain_IRP2KO/6A3-62.jpg]

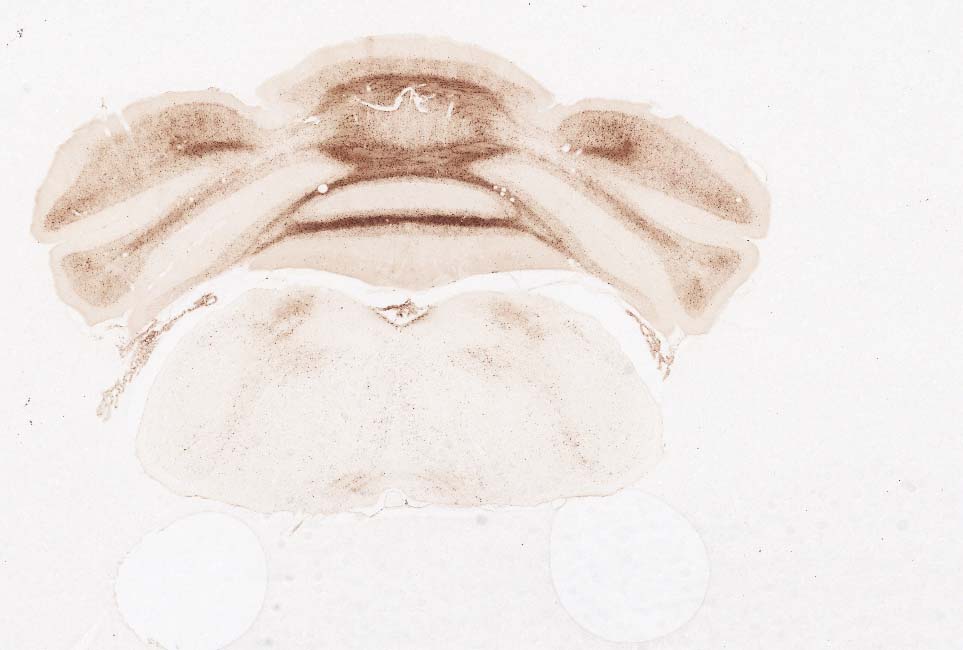

Supplement: File S2 — DAB-enhanced Perls' iron staining of coronal sections of Irp2−/− (6A-3) brains. Images (10–66) are from rostral to caudal. High resolution files are available from the corresponding author. (ZIP) [file pone.0098072.s013.zip › PerlsIronStain_IRP2KO/6A3-63.jpg]

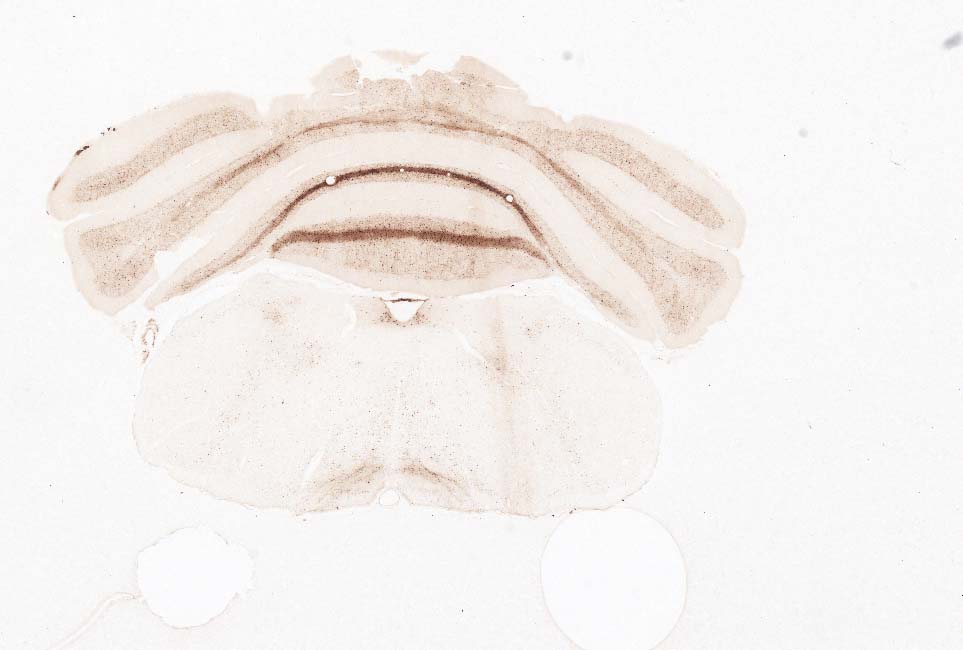

Supplement: File S2 — DAB-enhanced Perls' iron staining of coronal sections of Irp2−/− (6A-3) brains. Images (10–66) are from rostral to caudal. High resolution files are available from the corresponding author. (ZIP) [file pone.0098072.s013.zip › PerlsIronStain_IRP2KO/6A3-64.jpg]

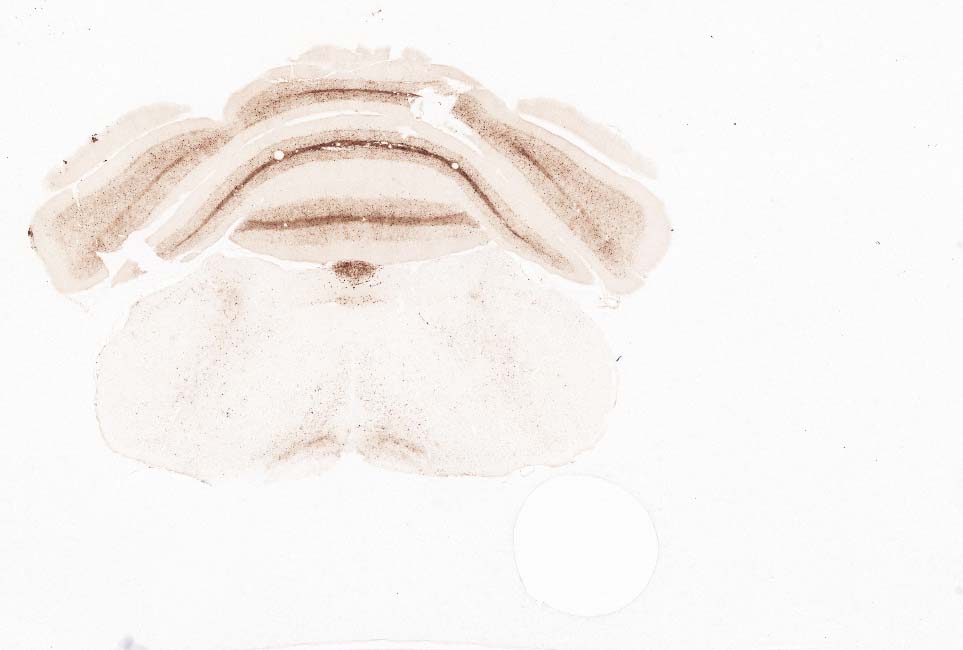

Supplement: File S2 — DAB-enhanced Perls' iron staining of coronal sections of Irp2−/− (6A-3) brains. Images (10–66) are from rostral to caudal. High resolution files are available from the corresponding author. (ZIP) [file pone.0098072.s013.zip › PerlsIronStain_IRP2KO/6A3-65.jpg]

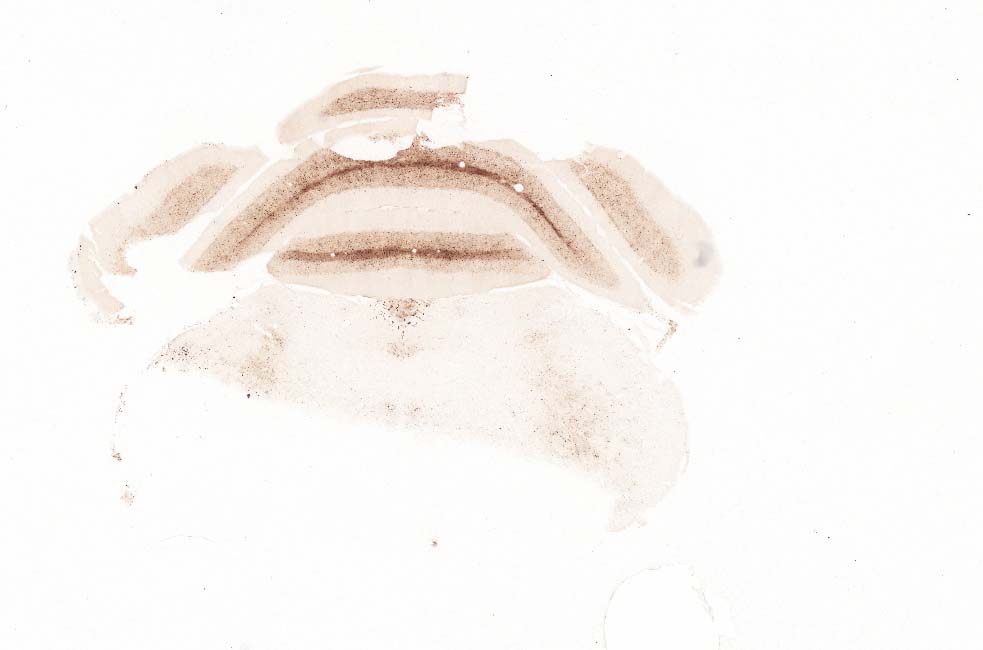

Supplement: File S2 — DAB-enhanced Perls' iron staining of coronal sections of Irp2−/− (6A-3) brains. Images (10–66) are from rostral to caudal. High resolution files are available from the corresponding author. (ZIP) [file pone.0098072.s013.zip › PerlsIronStain_IRP2KO/6A3-66.jpg]
